# Ebola virus nucleoprotein interaction with host protein phosphatase-1 regulates its dimerization and capsid formation

**DOI:** 10.1016/j.jbc.2025.108541

**Published:** 2025-04-25

**Authors:** Asrar Ahmad, Bersabeh Tigabu, Andrey Ivanov, Marina Jerebtsova, Tatiana Ammosova, Palaniappan Ramanathan, Namita Kumari, Christine A. Brantner, Colette A. Pietzsch, Jyothirmai Simhadri, Ghadeer Abdullah, Vladmir N. Uversky, Victor Paromov, Anastas Popratiloff, Steve Widen, Alexander Bukreyev, Sergei Nekhai

**Affiliations:** 1 Center for Sickle Cell Disease, Howard University, Washington, District of Columbia, USA; 2 Department of Pathology, University of Texas Medical Branch at Galveston, Galveston, Texas, USA; 3 Department of Microbiology, College of Medicine, Howard University, Washington, District of Columbia, USA; 4 Department of Medicine, College of Medicine, Howard University, Washington, District of Columbia, USA; 5 Galveston National Laboratory, University of Texas Medical Branch at Galveston, Galveston, Texas, USA; 6 GW Nanofabrication and Imaging Center, The George Washington University, Washington, District of Columbia, USA; 7 Department of Biology, College of Art and Science, Howard University, Washington, District of Columbia, USA; 8 Department of Molecular Medicine and USF Health Byrd Alzheimer's Research Institute, Morsani College of Medicine, University of South Florida, Tampa, Florida, USA; 9 Meharry Proteomics Core, RCMI Research Capacity Core, School of Medicine, Meharry Medical College, Nashville, Tennessee, USA; 10 Department of Biochemistry and Molecular Biology, The University of Texas Medical Branch at Galveston, Galveston, Texas, USA; 11 Department Microbiology & Immunology, University of Texas Medical Branch at Galveston, Galveston, Texas, USA

**Keywords:** ebola virus, nucleocapsid, nuclear protein, protein phosphatase-1, 1E7-03, small molecule EBOV inhibitor, split NanoBiT, transmission electron microscopy

## Abstract

Ebola virus (EBOV) replication is regulated by the host protein phosphatases, PP1 and PP2A, which dephosphorylate the transcriptional cofactor of EBOV polymerase VP30. The PP1-targeting compound 1E7-03 induces VP30 phosphorylation and inhibits EBOV infection. Here, we investigate the broader role of PP1 in EBOV replication and transcription, including its interaction with nucleoprotein (NP). When EBOV-infected cells were continuously treated with 1E7-03, the NP E619K mutation was found and selected for further analysis. The NP E619K mutation moderately reduced the EBOV minigenome transcription, which was restored by the treatment with 1E7-03. Proteomics, immunoprecipitation, dimerization, split NanoBit, and colocalization analyses indicated that NP interacts with PP1 and that NP E619K mutations enhanced this binding. Treatment with 1E7-03 dissociated PP1–NP complex, but enhanced NP dimerization, which was more pronounced for NP E619K mutant. Mutation and deletion analyses pointed to several potential PP1-binding sites in NP that were located in the moderately disordered NP regions. When NP was co-expressed with VP24 and VP35, formation of EBOV capsids was impaired with NP E619K mutation. Treatment with 1E7-03 restored the capsid formation by the NP E619K mutant but inhibited capsids formed by WT NP. Our findings suggest that PP1 binds to NP and that this binding might regulate NP dimerization and capsid formation. Collectively, our results point to a new role for PP1 in EBOV replication, in which NP binding to PP1 may facilitate viral transcription by delaying capsid formation and EBOV replication.

Ebola virus (EBOV) causes a deadly human disease (1); the 2013 to 2016 epidemic in West Africa was the most severe on record, with 28,000 cases and 11,000 deaths (2). The major outbreak in the Democratic Republic of the Congo in 2018 to 2020 resulted in over 3400 confirmed cases and 2280 deaths (3), followed by additional outbreaks and cases (https://www.cdc.gov/vhf/ebola/history/chronology.html). Consequently, it is essential to create new treatments and drugs against EBOV.

The nonsegmented EBOV genome of negative polarity comprises seven genes encoding eight proteins: nucleoprotein (NP), polymerase cofactor VP35, matrix protein VP40, glycoprotein GP and secreted glycoprotein sGP, transcriptional activator VP30, membrane-associated protein VP24, and RNA-dependent RNA polymerase L (4). We previously found that inhibition of the host protein phosphatase-1 (PP1) induces VP30 phosphorylation, thereby shifting the balance of transcription-replication activity of the EBOV polymerase complex toward replication (5). Recently, NP was reported to recruit the host protein phosphatase PP2A-B56, which dephosphorylates the VP30 N-terminal serine cluster, resulting in the upregulation of viral transcription (6). A subsequent study showed that serine-arginine protein kinase 1 interacts with the R_26_xxS_29_ motif of VP30 and phosphorylates VP30 Ser-29 (7).

PP1 and PP2A belong to the phosphoprotein phosphatase superfamily. The PP1 holoenzyme is composed of a catalytic subunit (PP1α, PP1β, or PP1γ) and a regulatory PP1-interacting protein that directs PP1 to a specific location in the cell and determines its activity and substrate specificity (8). Over 200 validated PP1 interactors bind to the PP1 catalytic subunits using multiple binding motifs, such as RVxF, SpiDoc, SILK, MyPhoNE, ΦΦ, and NIPP1-helix (9, 10). Small molecules that can compete with these docking motifs can be used to block the binding of the interactors to PP1, thus functionally disrupting distinct PP1 holoenzymes and inhibiting PP1-mediated processes. Thus, we envisioned that a PP1-targeting small molecule can inhibit viruses that engage PP1 by changing the phosphorylation of viral or host proteins involved in viral replication. Given the prevalence of the RVxF-type docking motif among 95% of PP1 interactors (11), we developed a series of small molecules to target the RVxF-motif binding pocket *in silico* and identified compounds 1H4 (12) and 1E7-03 (13). *In vivo*, 1E7-03 was effective against HIV-1 (14) and reduced LPS-induced lung inflammation in HIV-1 transgenic mice mediated by HIV-1 gene expression in macrophages (15, 16). We tested 1E7-03 against EBOV and showed that it induces VP30 phosphorylation and effectively suppresses EBOV infection in cell cultures (5). Moreover, we found that 1E7-03 inhibits Marburg virus (17). In addition, 1E7-03 inhibited Rift Valley fever virus (18, 19) and Venezuelan equine encephalitis virus (20). Treatment with 1E7-03 shifted the transcription/replication balance of the EBOV polymerase complex towards replication (5). However, 1E7-03 is quickly degraded in mice (14), and its degradation products had no suppressive effects against EBOV (21). We identified various analogs of 1E7-03, such as C31 and 1E7-07, that were stable in mice or mouse serum but were about 10 times less effective as EBOV inhibitors (21, 22). Thus, 1E7-03 remains the best PP1-targeting inhibitor of EBOV to date.

In the current study, we analyzed the effects of 1E7-03 on EBOV replication during a continuous long-term virus culture. We detected a mutation, NP E619K, after four passages of the virus in cells in the presence of 1E7-03. To explore the involvement of PP1 in EBOV replication, we examined the effects of the NP E619K mutation on viral transcription and replication, interaction of NP with PP1 and PP2A, NP dimerization, and viral capsid formation. Quantitative proteomics analysis was used to analyze PP1 binding to WT NP and NP E619K mutant. The split NanoBiT system was utilized to assess the dimerization of NP and its binding to PP1, PP2A, and VP30. Dithiobis[succinimidylpropionate] (DSP)-crosslinking experiments were utilized to investigate whether NP E619K may oligomerize/polymerize and the effect of 1E7-03 on this process. Fluorescent microscopy was employed to determine colocalization of PP1 with NP in live cells using NP-mCherry and PP1-eGFP fusions. Transmission electron microscope (TEM) analysis was utilized to determine the effect of NP E619K mutation on capsids formation in the cells expressing NP, VP35, and VP24. We also utilized AlphaFold to build and visualize NP, NP-PP1, and NP-NP-PP1 structures. Collectively, our study suggests that the NP E619K mutation enhances PP1 binding to NP. The mutation reduces NP polymerization and disrupts capsid formation. Our findings suggest that the host PP1 has a unique role that involves interacting with NP and controlling its dimerization and EBOV capsid formation.

## Results

### EBOV adaptation to 1E7-03 treatment

**Figure 1:**
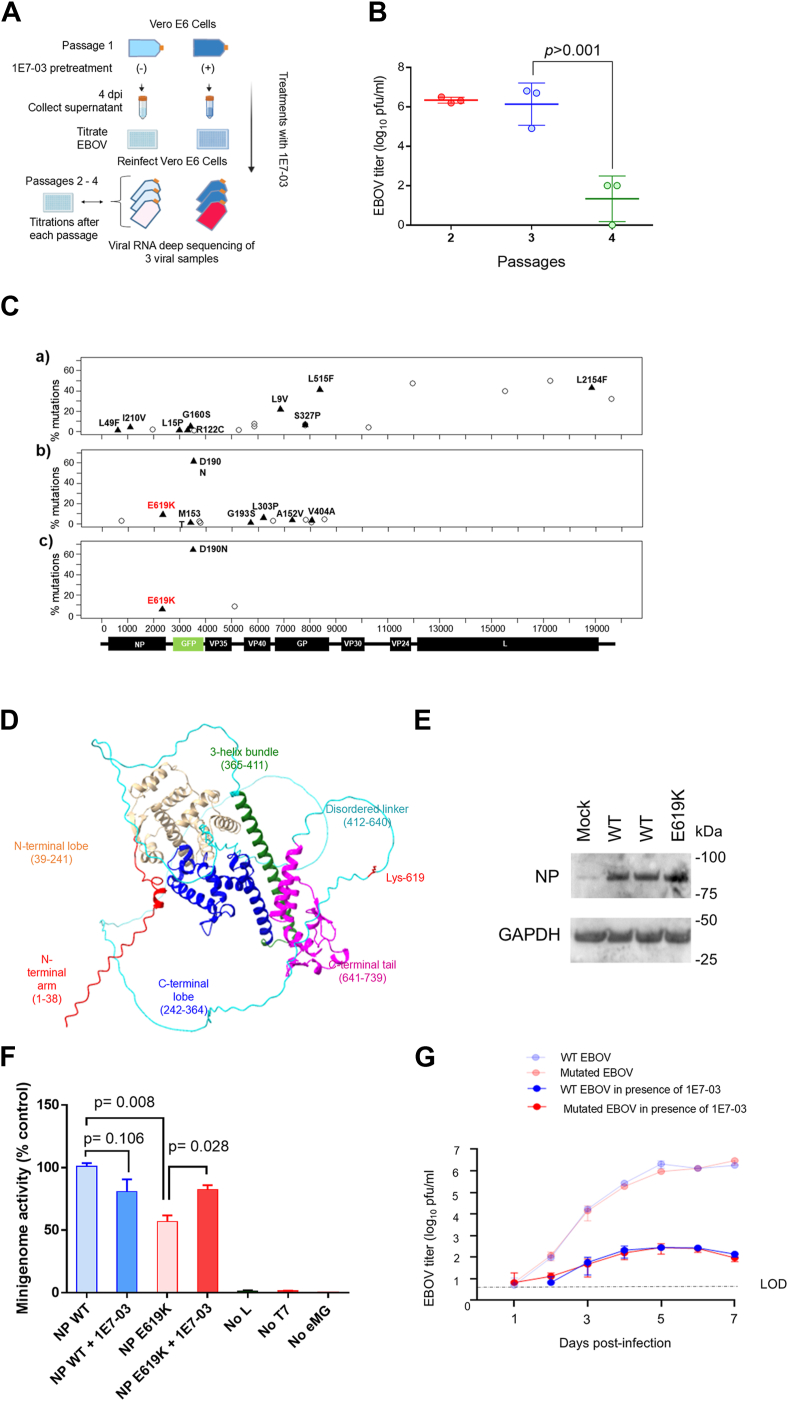
**EBOV adaptation to 1E7-03.***A*, workflow showing long-term 1E7-03 treatment strategy of EBOV-infected Vero E6 cells. Vero-E6 cell monolayers were infected with EBOV-eGFP, cultured with or without 1E7-03, and supernatants were collected at 10 to 11 days post infection and used for reinfection. *B*, EBOV replication from the virus collected after the indicated passages. Mean ± SD viral titers based on triplicate samples. Statistical comparison was done with Student's *t* test. *C*, triplicate viral RNA samples from passage four were deep sequenced. The frequencies of the identified mutations across the EBOV genome are shown (a,b,c). Missense mutations are shown as *shaded* triangles, and silent mutations are shown as *open* circles. Schematics of the EBOV-eGFP genome shows the ORFs of individual viral genes and eGFP. The *E619K* mutation in the *NP* gene detected in two of the three replicates is highlighted in *red*. *D*, the NP protein structure built by AlphaFold 3 and visualized in Chimera 1.3 showing NP structured domains as well as NP E619K mutation. *E*, NP WT and NP E619K subcloned into the pCEZ vector were expressed in Vero-E6 cells. NP protein expression was analyzed by Western blot with anti-NP antibodies and a loading control GAPDH antibodies. *F*, EBOV transcription activity of NP E619K mutant determined by minigenome assay with either WT NP or NP E619K mutant. The cells were additionally treated with 3 μM 1E7-03. At 48 h post-transfection, luciferase activity was measured. Data are the mean ± SD of triplicates. *G*, kinetics of EBOV replication with *NP E619K* mutation. EBOV titers were measured for the virus collected at 1-, 3-, 5-, and 7-days post infection. The data are the triplicates' mean ± SD.

We have previously demonstrated that PP1-targeting 1E7-03 and its derivatives are effective in EBOV inhibition (5, 21, 22). To further investigate the mechanism of 1E7-03–mediated inhibition of EBOV replication, we conducted a continuous viral culture study (workflow shown in Fig. 1*A*). First, Vero-E6 cells were pretreated with 3 μM 1E7-03 for 24 h and then infected with a recombinant EBOV that expresses eGFP (EBOV-eGFP) (23) at a multiplicity of infection of 0.01 PFU/cell. The cells were treated with 3 μM 1E7-03 every 24 h for 4 days (Fig. S1). After 4 days, the supernatants were collected; virus titer was determined and used in the following round of infection. The maximum available amount (15 PFU) was used to infect the cells in the next passage. Passage 2 was performed in the same way, except that the treatment was extended to 11 days (Fig. S2). On day 11, the supernatants were collected, titrated, and used to infect the monolayers for passage 3. The third passage began with a MOI 0.01 PFU/cell and was continued for 11 days with daily 3 μM 1E7-03 treatments (Fig. S3). The supernatant from day 11 was used to infect the monolayers for passage 4, which continued for 11 days with daily 3 μM 1E7-03 treatment. Viral titers from passages 2 and 3 were equal, while the titer from passage 4 was lower (4.8 log_10_ reduction, Fig. 1*B*). Three viral samples were isolated from both 1E7-03 treated and untreated cultures from day 11 supernatants of passage 4. Deep sequencing of the viral RNA was performed to identify mutations present in the genome of viruses isolated from untreated and the 1E7-03–treated cells. Several mutations in *NP*, *VP40*, *GP*, *VP24*, *VP30*, and *L* were found in viruses isolated from 1E7-03–treated cells (Table S1). Samples obtained from the untreated cells only exhibited mutations in the *VP24* which were the same as in the samples from 1E7-03–treated cells (Table S2). Comparison of the samples obtained from the treated cells identified the mutations *GP* *S327P* and *NP E619K* present in two out of three samples (Table S1). However, only the *NP E619K* mutation had a significant frequency in more than one sample (Fig. 1*C*). Moreover, the *NP E619K* mutation had the most significant *p*-value in sample 2 and was the only single mutation observed in sample 3 (Table S1). Therefore, we focused our subsequent analysis on the *NP E619K* mutation.

In NP protein structure built by AlphaFold 3 (24), the NP E619K mutation is located within the unstructured linker that connects the N-terminal structured lobes and the C-terminal tail of NP (25) (Fig. 1*D*). Comparison of NP E619K structure to the NP structure also built by AlphaFold 3 showed changes in the folding of the C-terminus of the NP mutant, while the N-terminus (residues 1–411) remained similarly folded (Fig. S4, panels A and B). To investigate whether the NP E619K mutation affects NP expression, it was introduced in the NP expression vector, and the expression was analyzed in Vero-E6 cells. An immunoblotting analysis showed similar expression levels for both WT NP and the NP E619K mutant (Fig. 1*E*). We then evaluated the impact of the NP E619K mutation on EBOV replication/transcription using an EBOV minigenome system (Fig. 1*F*). Vero-E6 cells were cultured in 24-well plates and transfected with the EBOV minigenome and plasmids expressing components of the EBOV polymerase complex (L, VP35, and VP30) under the control of T7 polymerase and a T7 polymerase-expressing vector (5). Additionally, WT NP or the NP E619K mutant were expressed from cotransfected plasmids with the cytomegalovirus (CMV) promoter controlling the expression. Renilla luciferase activity was measured 48 h after the transfection. When compared with the WT NP, the mutation resulted in a moderate, approximately two-fold decrease in the minigenome reporter gene signal (Fig. 1*F*), indicating that NP E619K mutant was still functional during EBOV minigenome transcription. Treatment with 3 μM 1E7-03 for 18 h inhibited minigenome in which WT NP was expressed (Fig. 1*F*). In contrast, the 1E7-03 treatment increased minigenome expression when NP E619K was co-expressed (Fig. 1*F*), suggesting that mutation facilitates EBOV transcription in the presence of 1E7-03. The reintroduction of the *NP E619K* mutation to EBOV-eGFP caused a delay in replication kinetics at day 5 post infection compared to WT EBOV-eGFP (Fig. 1*G*). However, treatment with 3 μM 1E7-03 still effectively inhibited both viruses (Figs. 1*G* and S5), indicating that the adaptation was not sufficient to fully overcome the inhibition.

### NP E619K mutation enhances PP1 binding

**Figure 2:**
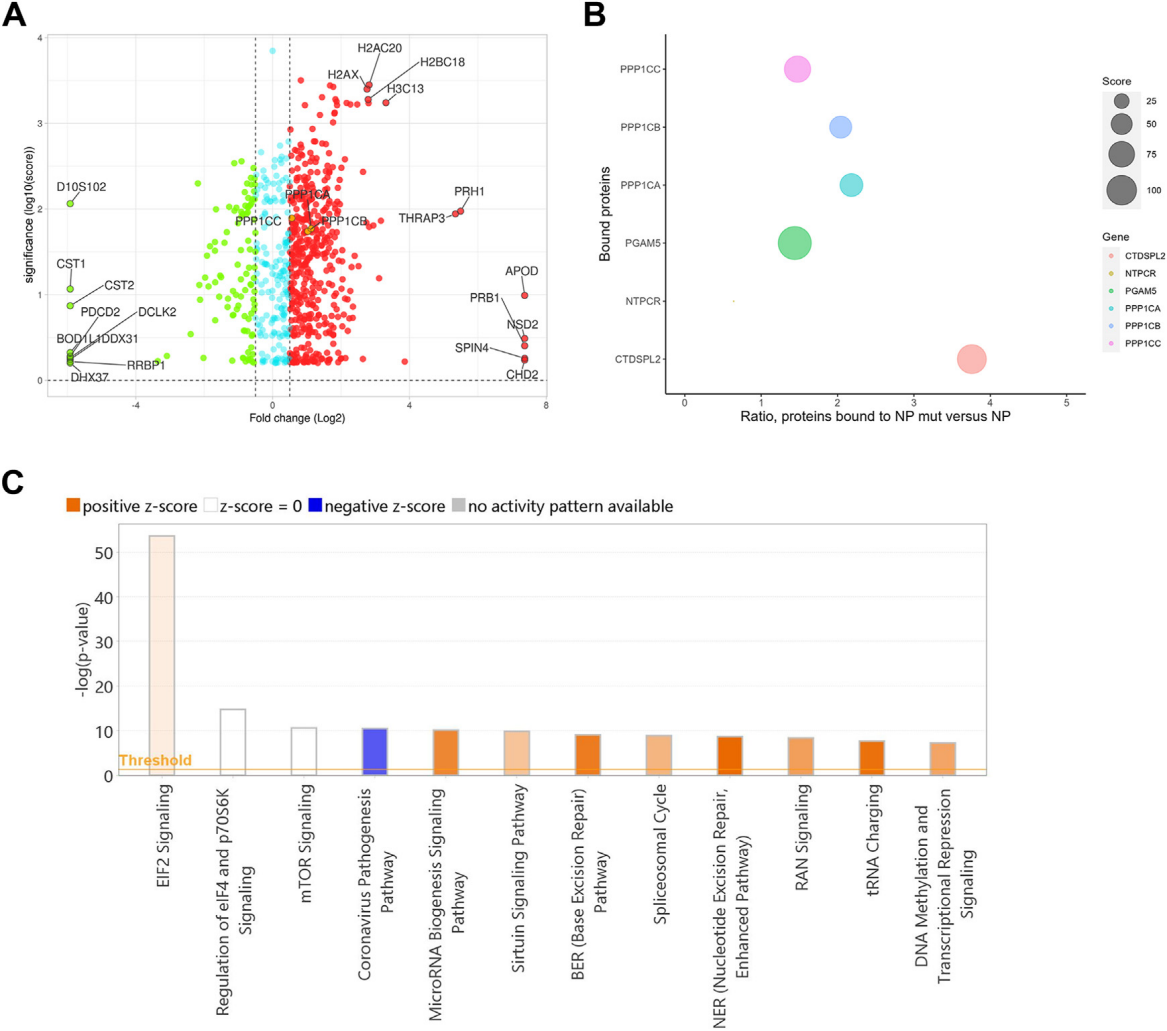
**Proteomic analysis of NP-interacting proteins.***A*, volcano plot shows host proteins bound to NP E619K *versus* WT NP with cutoff >1.5-fold (*red* color - upregulation; *blue* color - downregulation). X axis indicates fold change (Log2) and y axis indicates significance (log10 (score)). *B*, bubble plot showing NP interaction with all detected protein phosphatases. *C*, differentially bound proteins were analyzed by ingenuity pathway analysis (IPA) for canonical pathways. Colors indicate pathway activation with positive z-score (*orange*); no activation with z-score = 0 (*white*); pathway downregulation with negative z-score (*blue*); and no activity pattern (*gray*).

To obtain further insight into the NP E619K mutation, we analyzed host cells proteins bound to NP E619K in comparison to the proteins bound to WT NP. FLAG-tagged WT NP and NP E619K were expressed in HEK293T cells for 48 h and immunoprecipitated with a monoclonal anti-FLAG antibody. NP-associated proteins were resolved on 10% SDS PAGE and in-gel digested. Peptides were extracted and analyzed by tandem liquid chromatography Fourier transform mass spectrometry (LC-FT/MS) followed by label-free quantitative analysis using Proteome Discover 2.5. We detected peptides from 690 proteins bound to NP E619K *versus* WT NP including 150 proteins that were bound at higher levels to NP E619K (Fig. 2*A* and Table S3). Catalytic subunits of PP1 were present among these proteins (Fig. 2*A*). PP1 was bound at a ∼two-fold higher ratio to NP E619K than WT NP (Fig. 2*B*). PP1α was bound at the highest ratio among PP1 catalytic isoforms (Fig. 2*B*). Interestingly, no regulatory PP1 subunit was detected (Table S3). Proteins differentially bound to NP E619K *versus* WT NP were further analyzed by Ingenuity pathway analysis (IPA) software. NP E619K-interacting proteins positively correlated with EIF2, micro-RNA biogenesis, sirtuin and Ran signaling, base and nucleoside excision repair, tRNA charging, and DNA methylation and transcription signaling (Fig. 2*C*). Also, the bound proteins negatively correlated with the coronavirus pathogenesis pathway (Fig. 2*C*). Thus, NP E619K seems to be binding more efficiently to PP1. The bound proteins might affect signaling, DNA repair, and suppress transcription and negatively affect viral pathogenesis.

### The NP E619K mutant interaction with PP1

**Figure 3:**
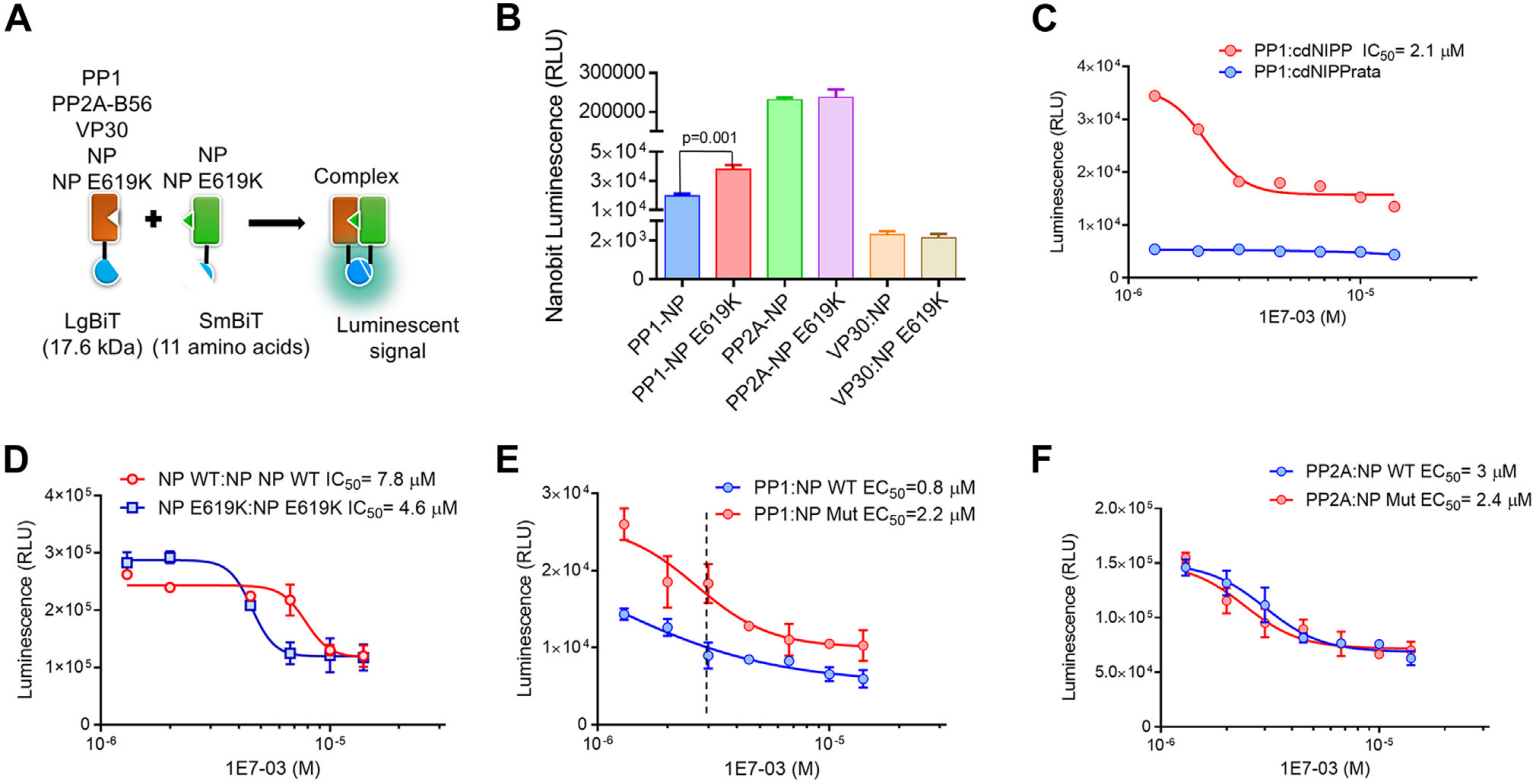
**EBOV NP E619K mutation downregulates NP-NP interaction and increases PP1 binding.***A*, a schematic representation of the split NanoBiT system, as well as the PP1 and NP constructions that were utilized. PP1, PP2A-B56, VP30, NP, and NP E619K were fused at the C-terminus of large Bit (LgBiT). NP or NP E619K was tagged at the N-terminus with small Bit (SmBit). Reconstitution of LgBit and SmBit produced luminescent signal in the presence of cell-permeable substrate furimazine. *B*, NP E619K mutation increases NP-PP1 interaction but had no effect on NP-PP2A-B56 or NP-VP30 interactions. NanoBit complementation assay was carried out as in (*B*) and data are shown as the mean ± SD from three independent experiments. *C*, 1E7-03 inhibits PP1:cdNIPP1 interaction. HEK293T cells were cotransfected by NanoBiT-tagged genes for PP1 and cdNIPP1 or PP1 and cdNIPP1 RATA for 24 h. The cells were then treated with different concentrations (1.3–14 μM) of 1E7-03 for an additional 6 h and NanoBiT luminescence activity was measured. Data are the mean ± SD of triplicates. *D*, 1E7-03 treatment reduces NP–NP interaction. Cells were cotransfected with NP:NP and NP E619K:NP E619K and the experiment was carried out as described in (A). Data are shown as mean ± SD. *E*-*F*, effect of 1E7-03 on PP1-NP and PP2A-B56-NP interactions. Cells were cotransfected with the indicated NanoBiT vectors and treated with 1E7-03. NanoBiT luminescence activity was measured and plotted as mean ± SD of triplicates.

Recently we have utilized a split NanoBiT-based system for studying the interaction of PP1 with its regulatory partners (22). By fusing PP1 to the large bit (LgBit) and NP to the small bit (SmBit), we were able to assess the effect of the NP E619K mutation on PP1 binding (see Experimental procedures for vectors details) (Fig. 3*A*). Co-expression of NP E619K with PP1 resulted in three-fold stronger NanoBiT signal comparing to when WT NP was co-expressed with PP1 (Fig. 3*B*). In contrast, NanoBiT signals were similar for WT NP or NP E619K mutant co-expressed with PP2A B56 subunit (Fig. 3*B*). Also, NanoBiT signal was similar for WT NP and NP E619K mutant co-expressed with VP30 (Fig. 3*B*). Taken together, these results suggest that the NP E619K mutant might have increased binding to PP1, whereas its binding to PP2A B56 subunit is likely to be similar to WT NP. Also, the E619K mutation does not seem to affect the interaction with VP30.

Next, we tested the effect of 1E7-03 on NP–NP and NP–PP1 interaction. We first assayed the effect of 1E7-03 on PP1 binding to its known interactor, cdNIPP1, and compared it to cdNIPP1 RVxF mutant (cdNIPP1rata, V201A/F203A mutation) that was deficient in PP1 binding as indicated by the reduced NanoBiT signal (70-fold reduction, Fig. 3*C*). For this analysis, HEK293T cells were cotransfected with the indicated NanoBiT plasmids for 24 h and then treated for 6 h with serial dilutions of 1E7-03 (1.3–14 μM). Treatment with 1E7-03 reduced the PP1-cdNIPP1 NanoBiT signal (IC_50_ = 2.1 μM) (Fig. 3*C*). The 1E7-03 also affected the NP-NP NanoBiT signal, but to a lesser extent (IC_50_ = 7.8 μM) (Fig. 3*D*). The 1E7-03 treatment also reduced NP E619K-NP E619 mutation NanoBiT signal (IC_50_ = 4.6 μM) (Fig. 3*D*). 1E7-03 had a much more pronounced effect on PP1-NP NanoBiT signal (IC_50_ = 0.8 μM) and less pronounced effect on PP1-NP E619K NanoBiT signal (IC_50_ = 2.2 μM) (Fig. 3*E*). Assuming that the NanoBiT signal reflects PP1-NP binding, the results indicate that the quantity of PP1 bound to the NP E619K mutant in the cells treated with 3 μM 1E7-03 would be equal to the quantity of PP1 bound to WT NP in the absence of 1E7-03 (Fig. 3*E*, dashed line). This assumption further suggests that the NP E619K mutation facilitates retention of PP1 in the presence of low micromolar concentrations of 1E7-03. Treatment with E7-03 had an equal suppressing effect on the NanoBiT signal for WT NP or NP E619K co-expressed with PP2A B56 subunit (Fig. 3*F*), further indicating that the mutation is not likely to affect PP2A binding to NP. Both PP1 and NP were co-expressed equally well in split NanoBit system (Fig. S6*A*). 1E7-03 treatment had no effects on PP1 expression, and only slightly reduced NP expression (Fig. S6, *B* and *C*). Thus, the effect of 1E7-03 was due to the reduction of PP1-NP binding and not their protein expression levels.

**Figure 4:**
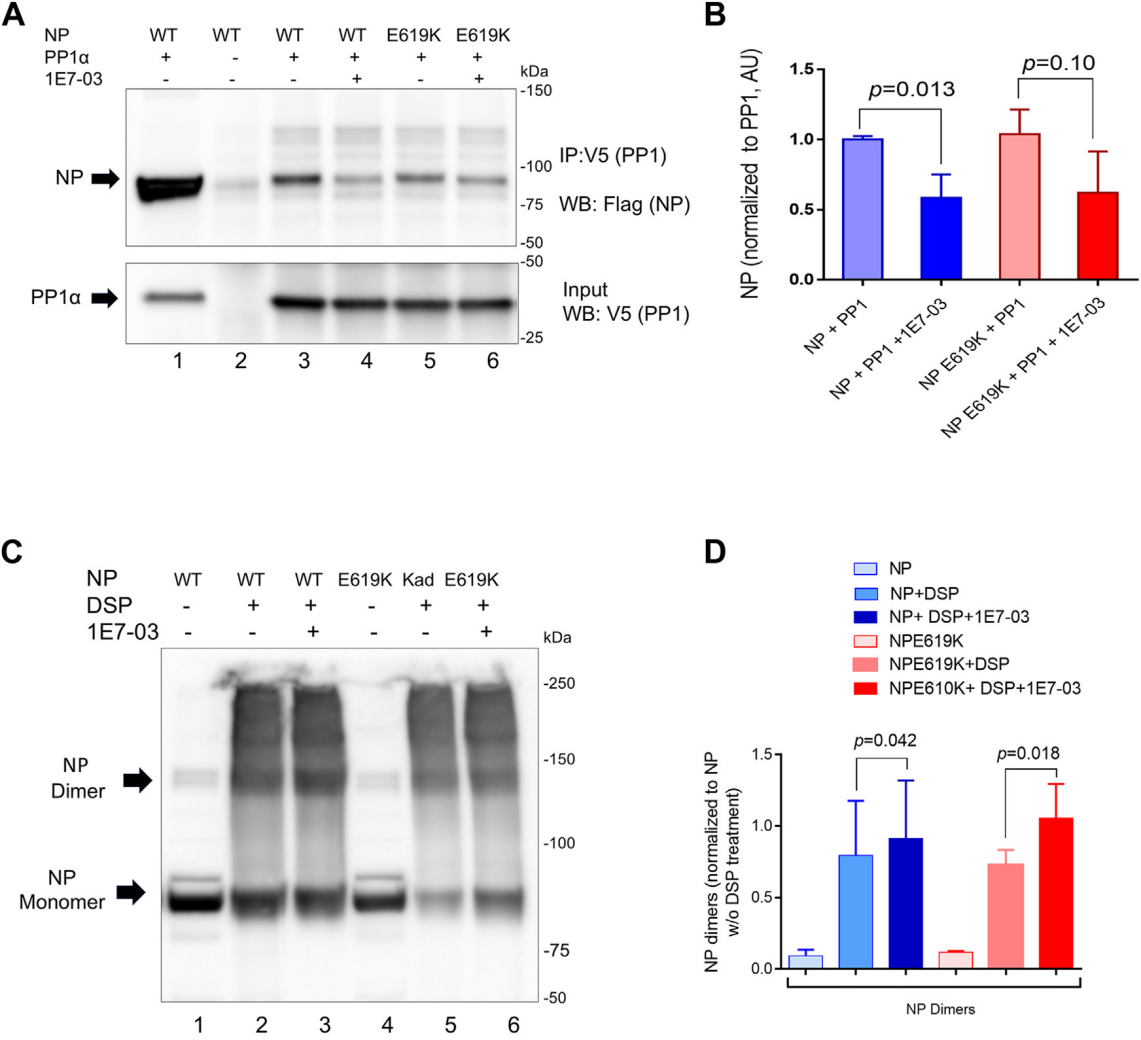
**Effect of 1E7-03 on the interaction of NP with PP1 and NP dimerization.***A*, NP binding to PP1 analyzed by immunoprecipitation. Flag-tagged NP (WT and E619K) and V5-tagged PP1α were expressed in 293T cells that were either treated with DMSO vehicle or with 10 μM 1E7-03 in DMSO for 24 h. At 48 h post transfection, the cells were harvested, lysed, and PP1 was immunoprecipitated with anti-V5 antibodies. Immunoprecipitated proteins were resolved on SDS PAGE, transferred to polyvinylidene difluoride membrane, and probed with anti-V5 and anti-Flag antibodies. Lane1, input cell lysate (only WT NP is shown). Lane 2, negative control with IgG used for immunoprecipitation. Lanes 3 to 6, PP1 was precipitated with anti-V5 antibodies. *B*, quantification of three independent experiments. *C*, NP dimerization analysis. 293T cells were transfected with vectors expressing NP WT or NP E619K and then either *left* untreated or treated with 3 μM 1E7-03 for 24 h. At 48 h post transfection, the cells were treated with 2 mM DSP for 45 min. The cell lysates were resolved on SDS PAGE under nonreducing conditions. Monomers and dimers are indicated by arrows. Lanes 1 and 4, cell lysate without DSP. Lane 2 and 5, NP expressing cells treated with DSP. Lanes 3 and 6, NP expressing cells treated with 1E7-03 and DSP. *D*, quantification of five independent experiments from (*C*). The pairwise statistical comparison was done with Student's *t* test.

To further investigate the effect of 1E7-03 on NP–PP1 interaction, we analyzed coprecipitation of NP with PP1. HEK293T cells were cotransfected with vectors expressing V5-tagged PP1α and FLAG-tagged NP. The cells were treated with either vehicle (dimethyl sulfoxide, DMSO) or 10 μM 1E7-03 for 18 h. PP1α and the associated proteins were immunoprecipitated with anti-V5 antibodies, resolved on 10% SDS polyacrylamide gel, and immunoblotted with anti-FLAG antibodies to detect the NP protein and anti-V5 antibodies to detect PP1 (Fig. 4*A*). Both WT NP and NP E619K bound PP1 (Fig. 4*A*, lanes 3 and 5). Treatment with 1E7-03 reduced PP1 binding to WT NP (Fig. 4*A*, lane 4 and quantification in Fig. 4*B*). In contrast, 1E7-03 had a trend towards reducing PP1 binding to NP E619K but no statistical significance was reached after three independent experiments (Fig. 4*A*, lane 6 and quantification in Fig. 4*B*).

We next assessed the effect of 1E7-03 on the dimerization of WT NP and NP E619K mutant using DSP crosslinking followed by nonreducing SDS gel electrophoresis (26). Crosslinking with DSP allowed the detection of both NP monomers and dimers (Fig. 4*C*). Treatment with 3 μM 1E7-03 increased the amount of WT NP dimers (Fig. 4*C*, lane 3 and quantification Fig. 4*D*). The effect was more pronounced for NP E619K mutant (Fig. 4*C*, lane 6), suggesting that NP E619K mutant more efficiently formed dimers in the 1E7-03–treated cells (Fig. 4*D*). These observations suggest that NP E619K was more efficient in forming dimers in the cells treated with 1E7-03 in comparison to WT NP.

**Figure 5:**
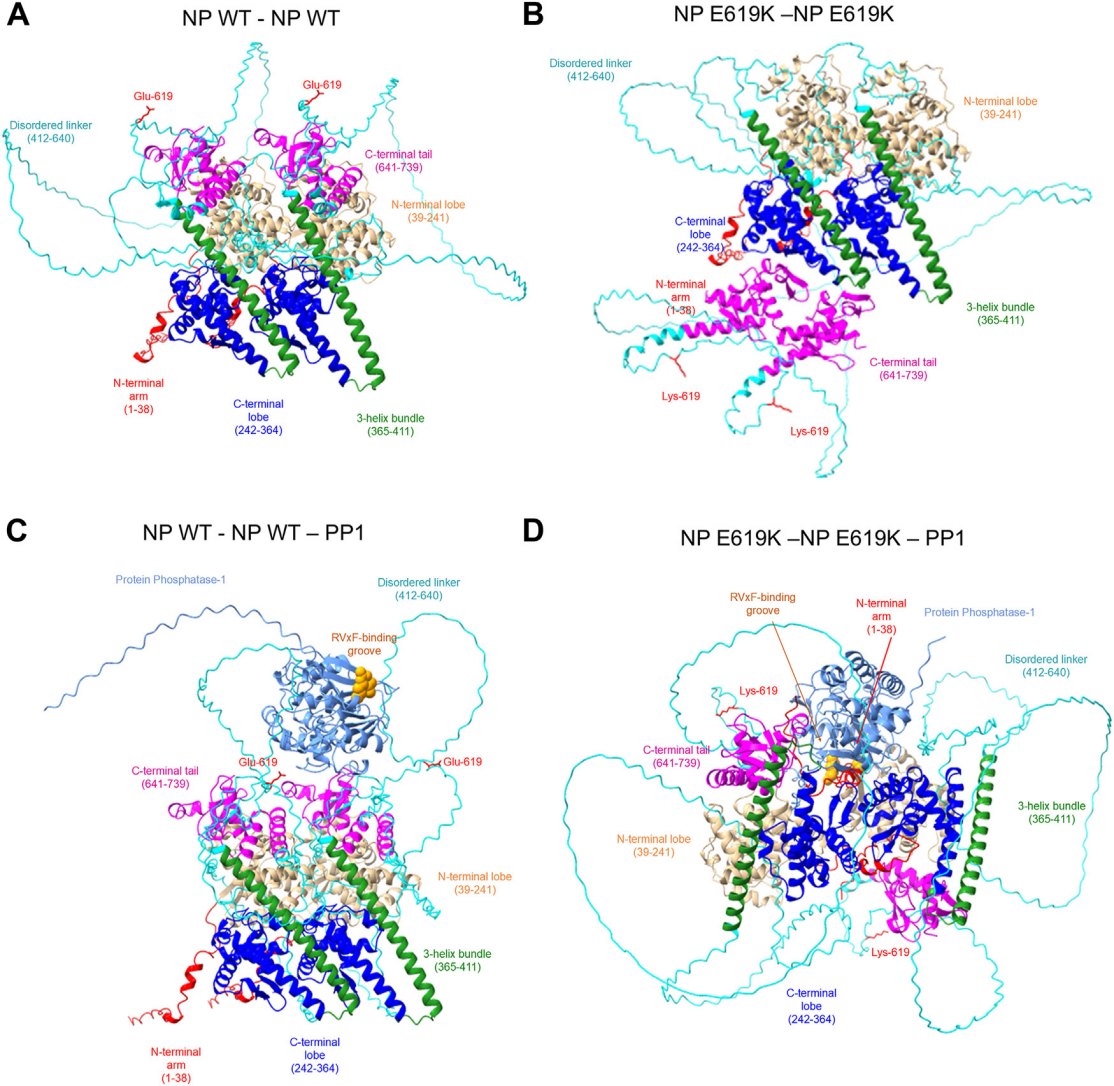
**Structures of NP dimers and NP-NP-PP1 trimers.** AlphaFold 3 was used to build structures of NP-NP dimer (*A*), NP E619K dimer (*B*), NP-NP-PP1 trimer (*C*), and NP E619K-NP E619K- PP1 trimer (*D*). The following NP's regions were shown in color: N-terminal arm (aa 1–38) in *red*; N-terminal lobe (aa 39–241) in *gold*; C-terminal lobe (aa 242–364) in *blue*; 3 helix bundle (aa 365–411) in *green*; disordered inker (aa 412–640) in *cyan* and C-terminal tail (aa 641–739) in *magenta*. PP1 in (*C*) and (*D*) is shown in cornflower *blue*. PP1's residues Arg-262 and Cys-291 are shown in *orange* as spheres to indicate the position of the RVxF-accommodating groove. NP's positions of Glu-619 (*A* and *C*) and Lys-619 (*B* and *D*) are shown in *red* with the side chains.

To visualize NP dimerization and the interaction with PP1, we generated NP dimers and NP–NP–PP1 trimers structures using AlphaFold 3. WT NP formed a dimer, primarily due to the interaction of the N-terminal lobe (Fig. 5*A*). NP E619K formed dimers but the C-terminal tails were folded differently and exposed to the solution (Fig. 5*B*). PP1–NP–NP trimers had similar NP dimer structure that seems to interact with PP1 through the flexible linker and C-terminal tail (Fig. 5*C*). The PP1s RVxF-accommodating groove was exposed to the solution (Fig. 5*C*). In contrast, PP1–NP E619K–NP E619K trimer formed a tighter complex between the NP dimer and PP1, in which the NP's N-terminal arms were interfacing with PP1 and the PP1's RVxF-accommodating groove was turned toward NP indicating a potential interaction (Fig. 5*D*). This analysis indicates that NP and NP E619K mutant might form distinct dimeric complexes and that PP1 interaction with these complexes may also be different.

We also examined the colocalization of NP with PP1 by utilizing live-cell fluorescence imaging of co-expressed NP-mCherry and PP1-eGFP fusion proteins (Fig. S7). We observed colocalization of NP and NP E619K with PP1α and PP1β/δ but not PP1γ (Fig. S7). We calculated Mander's coefficient to quantify the colocalization of WT and mutant NP, which indicated a significant increase in the colocalization of the NP E619K mutant with PP1α, as well as a trend towards increased colocalization with PP1 β/δ, as compared to WT NP (Fig. S8).

Overall, these observations indicate that the NP might bind PP1 and that NP E619K mutant might form a tighter complex with PP1 compared to WT NP, as indicated by the NanoBit, coprecipitation, dimerization, and colocalization analyses.

### Analysis of PP1-binding sites on NP

**Figure 6:**
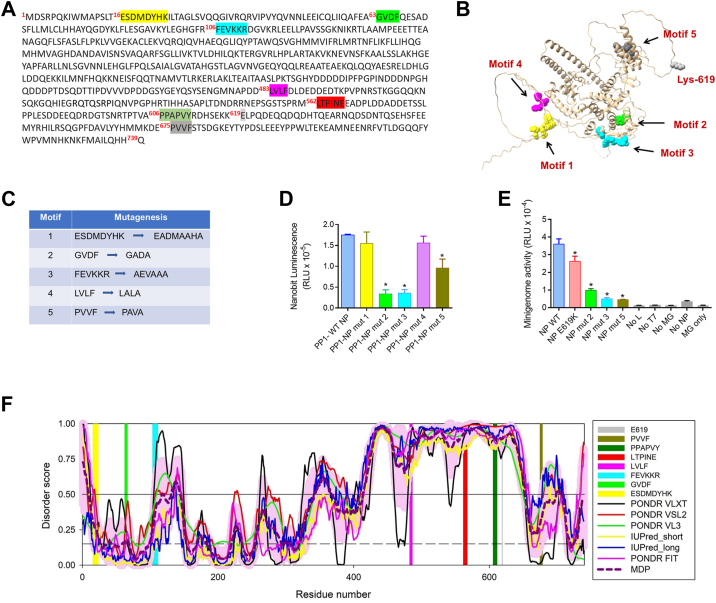
**Analysis of potential PP1-binding sites on NP and their effects on the interaction with PP1.***A*, potential PP1-binding motifs in the NP amino acid sequence are indicated in *yellow* (nonrelated, motif 1), *green* (RVxF, motif 2), *magenta* (RVxF-like, motif 3), *purple* (RVxF, motif 4), and khaki (RVxF, motif 5). Also shown are in *red* - PP2A binding site (LTPINE), in *forest green* - VP30 binding site (PPAPVY), in *gray* - Glu-619. *B*, NP structure built by AlphaFold 3 and colored in Chimera 1.3, showing location of potential PP1-binding motifs and position of E619K mutation. *C*, changes in the potential PP1-binding motifs introduced by site-directed mutagenesis. *D*, 293T cells were transfected with vectors expressing LgBiT-PP1 and indicated SmBit-NP–tagged mutants, and NanoBiT luminescence was measured at 24 h post transfection. Each value represents the mean ± SD from three independent experiments. ∗*p* < 0.01. *E*, effect of mutation in NP's PP1-binding sites on EBOV minigenome. 293T cells were transfected with the components of EBOV minigenome system except NP which was expressed from a plasmid under the control of CMV promoter. Luciferase activity was measured at 48 h post transfection. Data are the mean ± SD of triplicates. Statistical comparison was done with Student's *t* test. ∗*p* < 0.001. *F*, per-residue intrinsic disorder profile of the Ebola NP generated using the outputs of six commonly used per-residue disorder predictors PONDR VLXT (*black* line), PONDR VLS2 (*red* line), PONDR VL3 (*green* line), PONDR FIT (*pink* line), IUPred2_Short (*yellow* line), and IUPred2_Long (*blue* line), mean disorder profiles generated by averaging the outputs of all predictors (*bold dark pink dashed* lines), and the corresponding SDs (*light pink* shadow). The thresholds of intrinsic disorder (0.5) and structural flexibility (0.15) are shown as *black thin solid* and *dashed* lines, respectively. Position of PP1-binding sites as well as PP2A and VP30 binding sites are shown by differently colored vertical bars.

To identify PP1-binding sites on NP, we analyzed the presence of potential PP1-binding motifs within the NP sequence using multiple expectation-maximization (EM) for motif elicitation (MEME) program for the RVxF motif, KRPILG][V]x[FW] (27) and an alternative RVxF-like motif, [F]xx[KR]x[KR] (28). We identified three potential RVxF motifs, ^63^GVDF^66^, ^483^LVLF^486^, and ^675^PVVF^678^ (Table S4) and one noncanonical ^06^FEVKKR^111^ motif (Table S5) that were statistically significant according to MEME analysis (*p* < 0.0015, q value < 4). We also included one additional nonrelated sequence, ^15^ESDMDYHK^22^ (Fig. 6*A*). Motifs 1, 2, and 3 were located within the N-terminal NP core, whereas motifs 4 and 5 were located within the disorder linker and the C-terminal tail domain, respectively (Fig. 6*B*). These sites were mutated as indicated in Figure 6*C*, and their functionality was tested in NP-PP1 split NanoBit assay. As expected, the mutation of nonrelated motif 1 had no effect (Fig. 6*D*). Mutations of motifs 2 and 3 strongly reduced NP-PP1 NanoBit signal, indicating potential involvement of these sites in PP1–NP interaction (Fig. 6*D*). Mutation of motif 4 had no effect (Fig. 6*D*). Mutation of motif 5 showed a moderate reduction of NP-PP1 NanoBit signal (Fig. 6*D*). The functionality of motifs 2, 3, and 5 was further tested in EBOV minigenome system, which showed strong impact of mutations in motifs 2, 3, or 5 (Fig. 6*E*).

To next analyze the position of the potential PP1-binding sites in relation to the disordered domains within NP, disorder profiles for NP were obtained with the help of several disorder predictors (Fig. 6*F*). Interestingly, all the C-terminally located PP1-binding sites were found within a long MoRF/ANCHOR region (residues 394–679) predicted by IUPred2A (Fig. 6*F*). The PP1 motifs 2, 3, or 5 were located within the moderately disordered regions (green, blue, and khaki colors, disorder scores < 0.5) (Fig. 6*F*). In contrast, PP2A-binding site (LTPINE, shown in red, Fig. 6, *A* and *F*) and VP30-binding site (PPAPVY, shown in green, Fig. 6, *A* and *F*) are located in the highly disordered regions (disorder score > 0.75). The NP E619K mutation is also located in the highly disordered region (gray color, disorder score> 0.75, Fig. 6, *A* and *F*).

We visualized the location of the potential PP1-binding sites in the NP-PP1 structure using AlphaFold 3. Structure of NP-PP1 built by AlphaFold 3 showed that only motif 2 was located in the proximity of PP1, whereas other motifs were not likely to from direct interactions with PP1 (Fig. S9*A*). Structure of NP of the mutated motif 1 and PP1 showed minor changes in the NP-PP1 structure (Fig. S9*B*). Mutations of motif 2 and 3 significantly distorted NP–PP1 complex (Fig. S9, *C* and *D*). Mutation of motif 4 had little effect on the NP–PP1 complex (Fig. S9*E*), whereas mutation of motif 5 also distorted NP-PP1 structure (Fig. S9*F*). These computational visualizations further support the idea that motif 2 might be involved in interaction with PP1 and that the mutations in the motifs 2, 3, and 5 might distort NP–PP1 complex.

**Figure 7:**
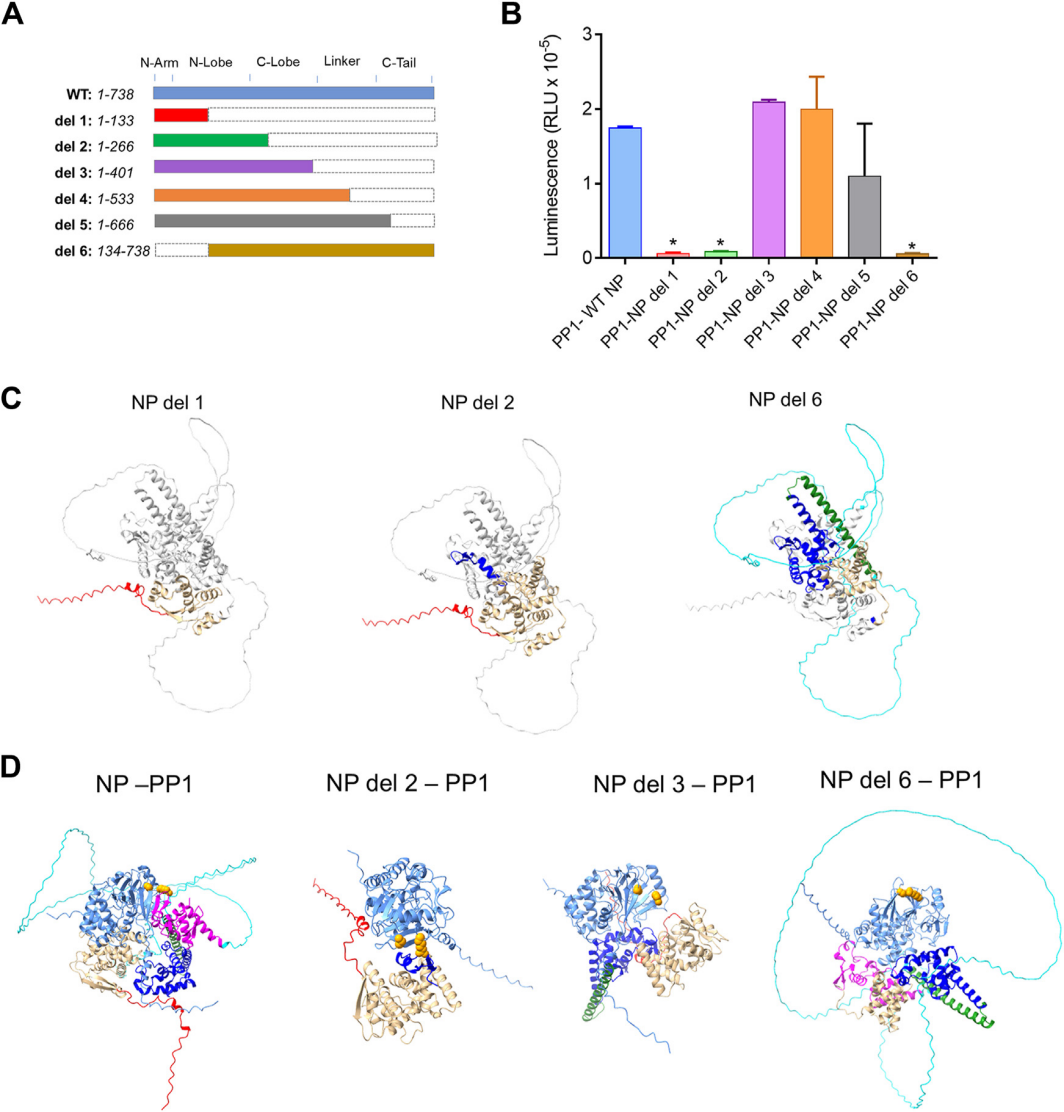
**Effects of NP deletion mutants on NP–PP1 interaction.***A*, NP deletion mutants generated to test NP binding to PP1. *B*, effect of NP deletions on PP1 binding. 293T cells were transfected with vectors expressing LgBiT-PP1 and indicated SmBit-NP deletion mutants. NanoBiT luminescence was measured at 24 h post transfection. Each value represents the mean ± SD from three independent experiments. Statistical comparison was done with Student's *t* test. ∗*p* < 0.01. *C*, NP structure built by AlphaFold 3 was used to visualize NP deletion mutants 1, 2, and 6. *D*, NP–PP1 complexes were built by AlphaFold 3 and further visualized in Chimera X 1.3. The following NP's regions are shown in color: N-terminal arm (aa 1–38) in *red*; N-terminal lobe (aa 39–241) in *gold*; C-terminal lobe (aa 242–364) in *blue*; 3 helix bundle (aa 365–411) in *green*; disordered inker (aa 412–640) in *cyan*, and C-terminal tail (aa 641–739) in *magenta*. PP1 is shown in cornflower *blue*. PP1's residues Arg-262 and Cys-291 are shown in *orange* as spheres to indicate the position of the RVxF-accommodating groove.

We next generated NP deletion mutants and tested their interaction with PP1 in split NanoBit system. Six NP deletion mutants spanned the entire NP sequence (Fig. 7*A*). The NP deletion mutants 1 and 2, which contained only parts of the N-terminal domain (residues 1–133 and 1–266, respectively) significantly reduced NanoBit signal indicating their reduced capacity to bind PP1 (Fig. 7*B*). The NP deletion mutants containing 1 to 401 amino acids or 1 to 533 amino acids had NanoBit signal similar to the level of WT NP–PP1 (Fig. 7*B*). The NP mutant containing 1 to 666 amino acids also showed strong NanoBit signal, suggesting that it retained the ability to bind PP1 (Fig. 7*B*). The NP lacking the first 133 amino acids had significantly reduced NanoBit signal (Fig. 7*B*). NP deletion mutants 1, 2, and 6 are illustrated in Figure 7*C*. The complexes PP1 with the deletion mutants 2, 3, and 6 were built by AlphaFold 3 (Fig. 7*D*). The deletion mutant 3 bound PP1 through the RVxF-binding site (Fig. 7*D*), whereas the deletion mutants 3 and 6 formed complexes with PP1 similar to WT NP (Fig. 7*D*). We further explored the deletion mutants 2, 3, and 6, by building complexes of their dimers and PP1 using AlphaFold 3 (Fig. S10). Compared to WT NP dimer in complex with PP1 (Fig. S10*A*), NP deletion 2 dimer had formed a different structure (Fig. S10*B*). NP deletion 3 dimer structure with PP1 was similar to the NP-NP-PP1 structure (Fig. S10*C*). However, the NP deletion six dimer formation was quite different from WT NP dimer with the C-terminus of NP being far apart (Fig. S10*D*), suggesting that this deletion might interfere with the NP–NP interaction. We also built structures of the deletion mutants with the 100 nt leader sequence of EBOV RNA (Fig. S11). Both WT NP and NP E619K dimers formed complexes with EBOV RNA (Fig. S11, *A* and *B*), although the E619K mutation affected the folding of the NP E619K dimers. Neither NP deletion 1 nor NP deletion 2 dimers formed a complex with EBOV RNA (Fig. S11, *C* and *D*). The NP deletion 6 dimer formed a complex with EBOV RNA but the dimer structure was distorted compared to the WT NP dimer structure with RNA (Fig. S11*E*), further suggesting that this deletion might interfere with the NP–NP interaction.

Based on these experiments, NP is likely to contain at least one PP1-binding site, although it is not likely to be an RVxF motif. The integrity of NP is also critical for PP1 binding as shorter NP fragments containing aa 1 to 266 or aa 1 to 133 are not likely to bind PP1. The minimal NP fragment that was able to bind PP1 contained aa 1 to 401, suggesting that PP1-binding site(s) are located within this fragment.

### The NP E619K mutation impairs EBOV capsid formation but facilitates capsid formation in the cells treated with 1E7-03

**Figure 8:**
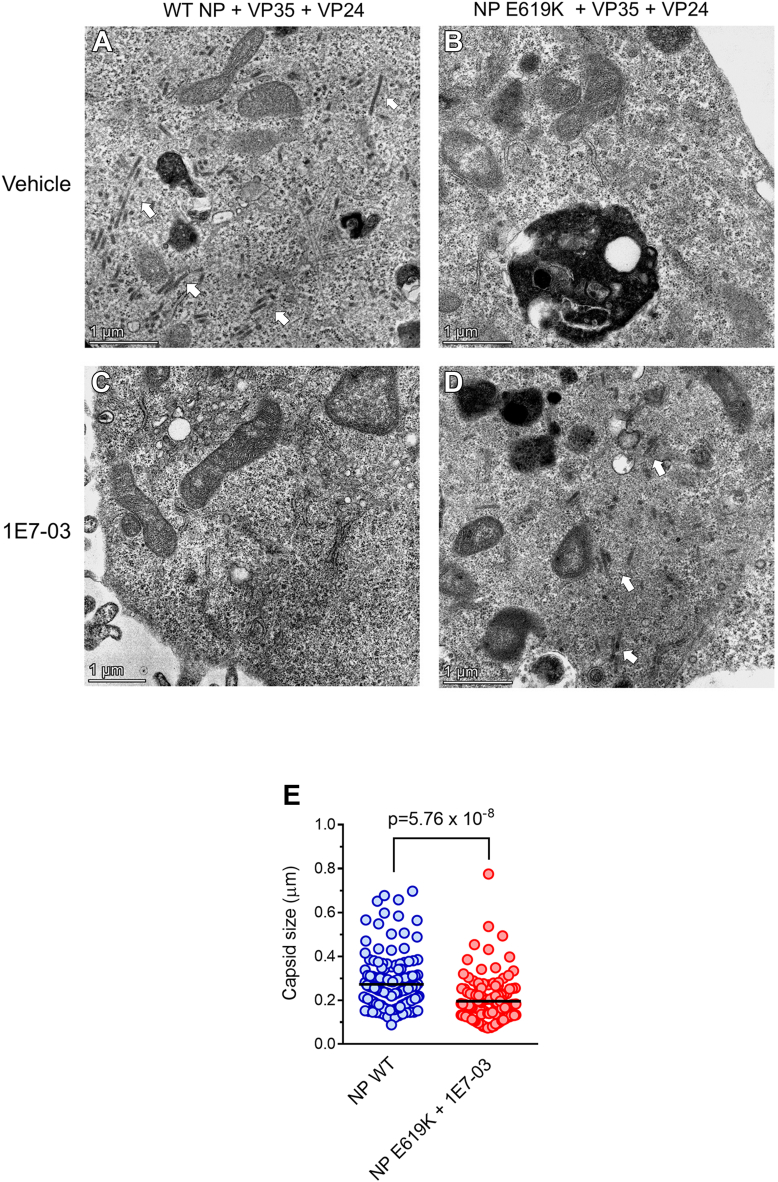
**EBOV capsid formation by NP E619K is facilitated by 1E7-03.** 293 cells were cotransfected with vectors expressing NP- mCherry, VP24, and VP35. Cells were fixed, stained, and sectioned as described in Experimental procedures. Imaging was performed on a Talos 200X transmission electron microscope. *A*-*D*, electron microscopy of 293 cells expressing WT NP, VP24, and VP35 (*A*) and treated with 1E7-03 (*C*), or NP E619K mutant, VP24, and VP35 (*B*) and treated with 1E7-03 (*D*). The bars indicate 1 μm scale. Arrows indicate capsid formation. *E*, linear size of capsids was measured in Image J. For WT NP, 14 images were used and for NP E619K + 1E7-03, six images were used. Statistical comparison was done with Student's *t* test.

Using TEM, we visualized the effect of NP mutation on EBOV capsid formation. We expressed either WT NP or the NP E619K mutant fused with mCherry in HEK293 cells along with VP35 and VP24 proteins. The WT NP was able to facilitate capsid structures formation (Fig. 8*A*). In contrast, no capsid formation was observed when the NP E619K mutant was used (Fig. 8*B*). Flow cytometry analysis revealed similar expression levels of WT NP and NP E619K (Fig. S12), indicating that the NP E619K mutant was well expressed and might be deficient in capsid formation. To assess the effect of 1E7-03 treatment on capsid formation, multiply TEM images of HEK293 cells expressing NP, VP35, and VP24 were analyzed after treatment with DMSO vehicle or 1E7-03. While capsids were absent in 1E7-03–treated cells expressing WT NP (Fig. 8*C*), capsids were observed in the cells expressing the NP E619K mutant and treated with 1E7-03 (Fig. 8*D*). We measured the linear size of EBOV capsids and found that they were 30% shorter in the cells expressing NP E619K mutant and treated with 1E7-03 compared to the cells expressing WT NP (Fig. 8*E*). These results suggest that the NP E619K mutation might facilitate the virus adaptation to 1E7-03 treatment and promotes capsid formation in the cells treated with 1E7-03.

Taken together, our data indicate that PP1 interacts with NP and that the E619K mutation in NP enhances this interaction, while affecting NP dimerization and compromising EBOV capsid formation. At the same time, the NP E619K mutation facilitates NP capsid formation in the presence of 1E7-03.

## Discussion

In this study, we extended our previous analysis of the role of PP1 in EBOV replication by investigating the long-term effects of 1E7-03 treatment in viral culture. We identified the NP E619K mutation developed during the long-term treatment of EBOV-infected Vero-E6 cells. This mutation had a moderate effect on EBOV transcription resulting in about 50% decrease in minigenome replication. However, this mutation facilitated EBOV capsid formation when cells expressing NP E619K were treated with 1E7-03. To obtain further insight into the effect of the NP E619K mutation, we analyzed NP E619K binding to PP1, PP2A B56, and VP30, using mass spectrometry, split NanoBiT system, and NP dimerization by crosslinking experiments. Quantitative mass spectrometry showed increased interaction of PP1 with NP E619K mutant. This observation was further supported by split NanoBit analysis showing increased split NanoBit signal for PP1-NP E619K interaction compared to PP1-WT-NP1. This observation suggests that NP E619K mutant might have enhanced PP1 binding while bound similarly to PP2 B56 or VP30. Analysis of the effect of 1E7-03 in the split NanoBiT system showed that at 3 μM 1E7-03 concentration, similar to what was used in viral passaging experiments, the NP E619K mutant retained PP1 binding comparable to untreated WT NP-PP1. Coprecipitation of PP1α with NP demonstrated that the NP interacted with PP1 and that this interaction was disrupted by 1E7-03, in contrast to the PP1–NP E619K interaction. Crosslinking with DSP showed that treatment with 1E7-03 increased dimerization of both WT NP and NP E619K, with having more pronounced effect on NP E619K. These observations suggested that PP1 might regulate NP dimerization. NP was found to colocalize with PP1α and PP1 β/δ, with the colocalization being particularly pronounced for NP E619K mutant. This observation agrees with the mass spectrometry and split NanoBiT results, which indicate enhanced interaction between NP E619K and PP1. Our analysis of PP1's interaction with NP revealed at least three potential PP1-binding sites. These sites were located in semidisordered regions of NP, in contrast to PP2A- and VP30-binding sites that were located in more disordered regions of NP. Also, NP E619K mutation is located in the highly disordered part of NP. Deletion analyses highlighted the importance of an intact N-terminal domain for the binding of PP1 to NP. Previously, NP was shown to undergo self-assembly through its N-terminal oligomerization arm, which facilitated RNA binding (29). Whether PP1 binds to NP directly or indirectly through a PP1 regulatory subunit capable of binding RNA remains to be investigated. As PP1-interacting regulatory proteins include more than 200 validated members (9, 10), it is possible that one of these subunits with an RNA-binding capability is involved in this interaction. Further analysis with the use of techniques such as proximity labeling is required to identify this subunit and to better understand PP1 binding.

Our analysis of viral capsid assembly indicated a striking difference between the WT NP and the NP E619K mutant. In the cells expressing WT NP, VP35, and VP24, we observed the formation of viral capsids, whereas no capsids were detected in the cells that express the NP E619K mutant, VP35, and VP24. Furthermore, no capsids were found in WT NP-, VP35-, and VP24-expressing cells after treatment with 1E7-03. In contrast, capsids were observed in NP E619K-, VP35-, and VP24-expressing cells treated with 1E7-03, although the capsids were reduced in length. This observation agrees with the results showing enhanced dimerization of the NP E619K mutant and further suggests that EBOV has adapted to 1E7-03 by being able to form capsids under the selective pressure of the compound.

**Figure 9:**
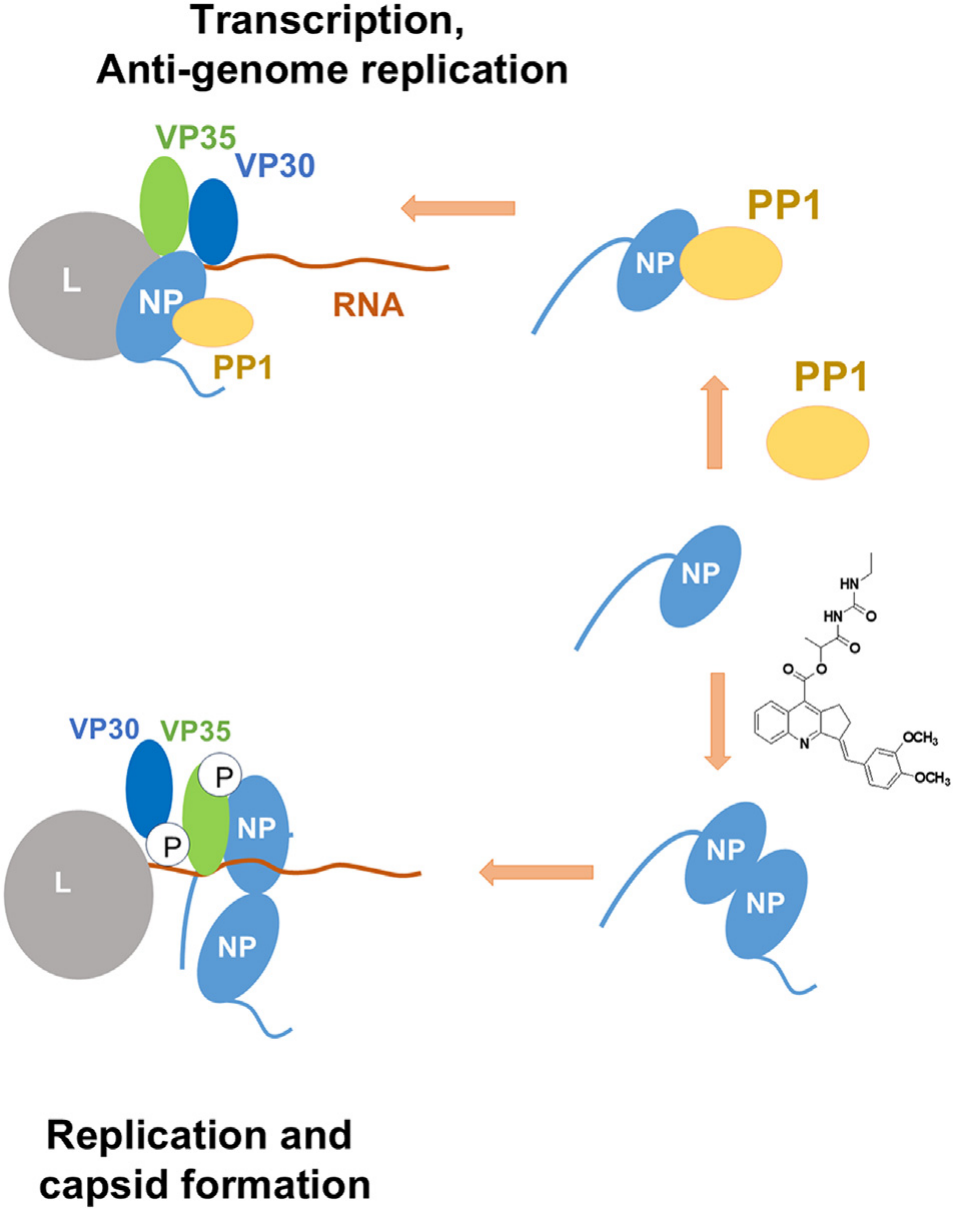
**Model of NP–PP1 interaction and the effect of 1E7-03 on capsid formation.** NP binding to PP1 prevents NP dimerization and facilitates NP recruitment to the EBOV transcription complex. Addition of 1E7-03 prevents PP1 binding to NP, facilitating NP dimerization and formation of EBOV replication complex. The enhanced association of NP E619K with PP1 prevents NP dimerization and capsid formation.

Our study points to the yet unrecognized role of PP1 in regulating NP dimerization and capsid assembly (summarized in Fig. 9). We propose that NP binds PP1, and this binding is a prerequisite for NP oligomerization and subsequent capsid formation. Treatment with 1E7-03 precludes PP1 binding with WT NP and might affect nucleocapsid formation. Treatment with the PP1-targeting 1E7-03 compound allows partial PP1 dissociation from the NP E619K mutant and might facilitate capsid formation by the NP E619K mutant. It is possible that some PP1 binding is required, but its access is inhibitory for capsid formation. In accord with the previous study (6), we observed strong NP interaction with PP2A, which was not affected by NP E619K mutation. We hypothesize that NP might interact with both PP2A and PP1 and that PP1 and PP2A might work in concert. An example of concert recruitment and fine-tuning of PP1 and PP2A can be seen in RepoMan, a protein encoded by *CDCA2*, which acts as a scaffold for both PP1 and PP2A B56 (30, 31). Phosphorylation of RepoMan by Aurora B on Ser-893 and Thr-394 prevents PP1 binding (31, 32). Conversely, phosphorylation of RepoMan by CDK1 on Ser-591 promotes the recruitment of PP2A B56, which reverses Ser-893 phosphorylation and enables re-recruitment of PP1 and dephosphorylation of M phase proteins (33, 34, 35). In this regard, the NP E619K mutation, which introduces a positive charge instead of a negative charge, may mimic NP dephosphorylation which could facilitate PP1 recruitment. It is possible that the function of NP-bound PP2A may include dephosphorylation of an unknown NP residue(s) to aid PP1 recruitment, prevent capsid formation, and induce transcription. The N-terminal part of NP (the first 450 amino residues) is necessary for NP–NP interaction as well as the following 150 residues are critical for nucleocapsid formation and viral replication (36). Our previous global phosphoproteomic analysis of EBOV virions identified 20 NP phosphorylation sites (37). Fourteen of these sites (Thr-536, Ser-541, Thr-545, Thr-563, Thr-597, Ser-598, Thr-601, Thr-603, Tyr-686, Thr-687, Tyr-688, Ser-691, Tyr-696, and Thr-701) were found within the large unstructured sequence that connects the N-terminal and C-terminal domains of NP (37). Previous studies identified phosphorylation of Thr-563, Ser-581, Ser-587, and Ser-647 in NP expressed in cultured cells (38). As the existing crystal structures of EBOV NP only captures parts of the N-terminal region (29, 39, 40) and the C-terminal domain (41), we previously constructed a full-length model of NP using *de novo* prediction, which showed highly stable globular-like “structured” sections during an equilibrium molecular dynamics simulation in a periodic water box (37). The NP residues 412 to 645 were highly flexible during molecular dynamics simulation (37), suggesting that these residues are accessible for PP1 or PP1 regulatory subunit binding. We also identified 10 phosphorylation sites on VP35 that were detectable in EBOV virions (37). Interestingly, dephosphorylation of Thr-210 blocked EBOV transcription and prevented VP35 binding to NP (37), suggesting that NP-associated phosphatases can also control EBOV transcription by dephosphorylating VP35.

The NP E619K mutation resides near the VP30-binding PPxPxY motif that was also found in the host RBBP6 protein (42). Recently, a mass spectrometry approach identified additional PPxPxY-containing host proteins, including hnRNP L, hnRNPUL1, and PEG10 that all strongly interact with VP30 (43). While we did not observe any changes in VP30 binding to NP E619K, which had low NanoBiT signal, it is possible that the NP E619K mutation affects the VP30 exchange between host proteins and NP. Further analysis is needed to test the binding of NP E619K with host proteins and other factors such as VP35.

Taken together, our results point to a new unique role of host PP1 in EBOV replication in which PP1 might be involved in the interaction with NP and the control of NP dimerization and EBOV capsid formation.

The limitation of our study is the lack of more advanced proximity analysis and detailed electron microscopy that can be used in future to identity PP1 regulatory subunit(s) involved in NP binding and to analyze NP transcription complex by electron microscopy.

## Experimental procedures

### Experimental design and statistical rationale

Comparison of host cells proteins bound to EBOV WT NP protein and NP E619K mutant protein was conducted by tandem LC-FT/MS. FLAG-tagged WT NP and NP E619K were expressed in HEK293T cells for 48 h and immunoprecipitated with a monoclonal FLAG antibody. NP-associated proteins were resolved on 10% SDS PAGE and in gel digested. Peptides were analyzed by LC-FT/MS followed by label-free quantitative analysis on Proteome Discover 2.5. The NP E619K *versus* WT NP-bound proteins were further analyzed by IPA software with cutoff > 1.5 fold and *p*-value < 0.05.

### Chemicals and reagents

1E7-03 (purity above 98%) was synthesized by Enamine as previously described (13). DMSO, acetone, hydrochloric acid, and sodium hydroxide were obtained from Thermo Fisher Scientific. Sodium acetate (pH 5.2) was from Quality Biological. PBS (pH 7.4) was from Life Technologies.

### Cells and media

Vero-E6 and HEK293T cells were purchased from the American Type Culture Collection. Vero-E6 cells were cultured in modified Eagle medium (Life Technologies) with 10% fetal bovine serum (FBS) and 1% gentamicin (Life Technologies). The HEK293T cells were cultured in Dulbecco's modified Eagle's medium (Invitrogen) containing 10% FBS and 1% antibiotic solution (penicillin and streptomycin).

### Experiments with infectious EBOV

All experiments using infectious EBOV were performed under the Biosafety Level 4 containment of the Galveston National Laboratory. Vero-E6 cell monolayers were grown in 12- or 24-well plates and treated for 1 h at 37 °C, 5% CO_2_ with compounds diluted in maintenance MEM medium (Life Technologies) containing 2% FBS (Hyclone), 0.1% gentamicin sulfate (Corning), 1% nonessential amino acids (Sigma), and 1% sodium pyruvate (Sigma). Cell monolayers were infected with the recombinant EBOV that expresses eGFP (EBOV-eGFP) for 1 h at 37 °C (23). After adsorption, monolayers were washed three times with PBS, a fresh compound in the maintenance medium was added to each well, and monolayers were incubated at 37 °C for 4 days (passage 1), 11 days (passages 2 and 3), or 10 days (passage 4) postinfection.

To quantify EBOV-eGFP titers in Vero-E6 cell supernatants, confluent Vero-E6 monolayers were inoculated with 10-fold serially diluted supernatants, adsorbed for 1 h at 37 °C, 5% CO_2_, and subsequently coated with an overlay of the medium containing 0.9% methylcellulose (Sigma). Fluorescent viral plaques were counted after 4 days using a UV microscope.

### EBOV sequencing

Triplicate Vero-E6 cell monolayers were treated with 0.3 μM 1E7-03 for 24 h and then infected with recombinant WT EBOV-expressing eGFP at a MOI 0.01 PFU/cell. The treatment was repeated every 24 h for 4 to 11 days. Supernatants were collected and used for titration and infection during the next round of selection passage. The supernatants from prior passages were used to infect new Vero-E6 cell monolayers. The passages were repeated four times. At the end of each selection cycle, RNA was lysed in 1 ml of TRIzol (Thermo Fisher Scientific) for 10 min at room temperature, and the samples were removed from the Biosafety Level 4 containment. RNA isolated from cell monolayers after the fourth passage was sequenced. Quantification and purity of resuspended total RNA were determined by NanoDrop (Thermo Fisher Scientific). One to four micrograms of RNA was used to prepare libraries using the Ribo-Zero Stranded kit (Illumina) according to the manufacturer's recommendations, and 50 base paired end reads were sequenced on the HiSeq1500 sequencer.

Raw reads (50 bp) were mapped using the EBOV reference sequence (NCBI Reference Sequence: NC_002549.1) using BWA mem (version 0.7.15, http://bio-bwa.sourceforge.net/). Visualization of the reads across this reference was accomplished with the Integrative Genomics Viewer (http://software.broadinstitute.org/software/igv/) (44).

### Minigenome system

Minigenome was assembled as previously described (37) using pCEZ-NP, pCEZ-VP35, pCEZ-VP30, pCEZ-L, and pC-T7 plasmids kindly provided by Dr Yoshihiro Kawaoka. Vero-E6 cells were cotransfected with the minigenome plasmids using Mirus transfection reagent (Mirus Bio). Transcription was measured at 48 h post-transfection by the luciferase assay (Promega) and it was normalized to viable cell number.

### Plasmids

#### PP1 and cdNIPP1 expression plasmids

Plasmids expressing PP1α, PP1β, and PP1γ fused to eGFP, cdNIPP1 (Residues 140–225)-eGFP fusion, and cdNIPP1 RATA (V201A and F203A) mutant fused to eGFP were kindly provided by Mathieu Bollen (KU Leuven).

#### NP expression plasmids

The glutamic acid in position 619 of NP was substituted to lysine by overlap extension PCR using Pfx polymerase (Thermo Fisher Scientific) with two sets of external primers flanking 5′ and 3′ ends of pCEZ-NP sequence and two internal primers containing the mismatched bases. After sequence confirmation, the mutated plasmids were digested with MfeI and NheI enzymes and cloned back into pCEZ-NP vector. WT NP or NP E619K mutated plasmid were transfected in Vero-E6 cells using TransIT-LT1 Transfection Reagent (Mirus) at 3 μl per μg of plasmid DNA, and cell lysates were harvested 48 h post-transfection to confirm expression by Western blotting. Blots were stained with Rabbit anti-EBOV NP Antibody (IBT Bioservices) and GAPDH (14C10) Rabbit mAb (Cell Signaling).

To prepare WT NP-mCherry and NP E619K-mCherry expression vectors, pcDNA3.1(−) plasmid was digested with Not1 and Kpn1 restriction enzymes and purified on the agarose gel. The mCherry fragment was amplified by PCR from pcDNA3.1-mCherry plasmid with forward CATGGCAATCCTGCAACATCATCAGAAGGGCGAGGAGGATAACATGGCCATC and reverse GTTTAAACTTAAGCTTGGTACCTACTTGTACAGCTCGTCCATGCCGCCGGTGG primers. PCR fragments of WT NP and NP E619 K were amplified with forward AACGGGCCCTCTAGACTCGAGCGGCCGCATGGATTCTCGTCCTCAGAAAATCTGG and reverse GATGGCCATGTTATCCTCCTCGCCCTTCTGATGATGTTGCAGGATTGCCATG primers from pCEZ NP and pCEZ NP E619K expression vectors described above. All fragments were combined in one vector with a Gibson assembly kit (New England BioLab; cat# E2611S) according to the manufacturer's protocol.

#### NanoBiT vectors

We evaluated all combinations of expression constructs to determine the best combination and orientation for fusions of tested proteins to LgBiT and SmBiT, as recommended by Promega. To simplify multiple cloning procedures, the entry pF4A CMV vector was designed with SgfII and PmeI cloning sites. To clone the DNA fragments of all tested proteins, their sequences were PCR amplified with primers linked to SgfII and PmeI cloning sites. The WT NP and NP E619K mutant expression plasmids described above were amplified with forward GCGATCGCCATGGATTCTCGTCCTCAGAAAATCTGG and reverse GTTTAAACCTGATGATGTTGCAGGATTGCC primers. PP1 was amplified with GCGATCGCCATGTCCGACAGCGAGAAGCTCAACC and GTTTAAACGAATTCGAGCTCGGTA primers. The cdNIPP1 and cdNIPP1 RATA were amplified with the same GCGATCGCCATGGGTGGAGAGGATGATGAAC and GTTTAAACGAATTCGAGCTCGGTA primers because they have identical 5′ and 3′ ends. EBOV VP30 expression plasmid developed in Dr Bukreyev's lab was amplified with forward ACGACTCACTATAGGGCTAGCGATCGCCATGGAAGCTTCATATGAGAG and reverse GTACCGAGCTCGAATTCGTTTAAACAGGGGTACCCTCATCAGACCATGAGC primers. PP2 B56 clone was obtained from GenScript and amplified with forward GACTCACTATAGGGCTAGCGATCGCCATGTCGTCGTCGTCGCCGCCGGCGG and reverse TAGAGGATCCCCGGGTACCGAGCTCGAATTCGTTTAAACTTCGGCACTTGTATTGCTGAG primers.

Mutagenesis of potential PP1-binding motifs in the NP protein was carried out in pcDNA3.1(−) NP plasmid using the primers listed below. Mutation of sequence ESDMDYHK to EADMAAHA was carried out with forward GAGTCTCACTGAAGCTGACATGGCTGCCCACGCGATCTTGACAGCAG and reverse CTGCTGTCAAGATCGCGTGGGCAGCCATGTCAGCTTCAGTGAGACTC primers. Mutation of sequence GVDF to GADA was carried out with forward CAGGCCTTTGAAGCAGGTGCCGATGCTCAAGAGAGTGCGGAC and reverse GTCCGCACTCTCTTGAGCATCGGCACCTGCTTCAAAGGCCTG primers.

Mutation of sequence FEVKKR to AEVAAA was carried out with forward GAAGGGCACGGGTTCCGTGCTGAAGTCGCGGCGGCTGATGGAGTGAAG and reverse CTTCACTCCATCAGCCGCCGCGACTTCAGCACGGAACCCGTGCCCTTC primers. Mutation of sequence mutated LVLF to LALA was carried out with forward GCACCAGATGACTTGGCCCTAGCCGATCTAGACGAGGAC and reverse GTCCTCGTCTAGATCGGCTAGGGCCAAGTCATCTGGTGC primers. Mutation of sequence of the forward GATGAAGGATGAGCCTGCTGTTGCCAGTACCAGTGATG and reverse CATCACTGGTACTGGCAACAGCAGGCTCATCCTTCATC primers were used to mutate the sequence PVVF to PAVA. Further cloning of mutated NP was conducted exactly as described above. To facilitate exchange NP deletion mutants cloning, we created SgfII and PmeI cloning sites in pcDNA3.1(−) vector. Then PCR NP-amplified deletion products were cloned directly in the pF4A CMV entry vector. NP deletion mutant 134 to 738 amino acids (aa) was produced with forward ACGGGCCCTCTAGACTCGAGCGGCCGCGATCGCCATGGATTCTCGTCC and reverse TTTAAACTTAAGCTTGGTACCGTTTAAACTGTTCTCTTAATGTTTTTTCC primers. The forward ACGGGCCCTCTAGACTCGAGCGGCCGCGATCGCCATGGATTCTCGTCC and reverse TTTAAACTTAAGCTTGGTACCGTTTAAACAGGATGGAGACGAACTCCTCG primers were used to create the NP deletion 267 to 738 aa mutant. NP deletion mutant 402 to 738 aa was generated with forward ACGGGCCCTCTAGACTCGAGCGGCCGCGATCGCCATGGATTCTCGTCC and reverse TTTAAACTTAAGCTTGGTACCGTTTAAACCTCTTTTCTTAGAGTTACC primers. NP deletion mutant 534 to 738 aa was produced with forward ACGGGCCCTCTAGACTCGAGCGGCCGCGATCGCCATGGATTCTCGTCC and reverse TTTAAACTTAAGCTTGGTACCGTTTAAACAGGGCCTGGGACATTTTGAATTGG primers.

The NP deletion mutant 667 to 738 aa was produced with forward ACGGGCCCTCTAGACTCGAGCGGCCGCGATCGCCATGGATTCTCGTCC and reverse TTTAAACTTAAGCTTGGTACCGTTTAAACCAAAACAGCATCAAATGGCCCCTG primers. Subsequently, NP deletion mutant 1 to 133 aa was produced with forward ACGGGCCCTCTAGACTCGAGCGGCCGCGATCGCCATGCTTGCTGCCATGCCGGAAGAGG and reverse TTTAAACTTAAGCTTGGTACCGTTTAAACCTGATGATGTTGC primers.

All described above pF4A CMV entry vectors were linearized with SgfII and PmeI restriction enzymes. Further cloning to LgBiT and SmBiT was done by enzymatic exchange with the entry pF4A CMV Flexi vectors carrying DNA fragments of all tested proteins. As a result, we obtained vectors expressing all tested proteins fused to N and C termini of both LgBit and SmBit. Their eight combinations were tested in NanoBiT experiments to detect the optimal combination pair.

### LC-FT/MS analysis

LC-FT/MS analysis was performed on an UltiMate 3000 RSLCnano System coupled to Orbitrap Exploris 480 Mass Spectrometer (Thermo Fisher Scientific) with the installed Xcalibur software (version 4.4, Thermo Fisher Scientific). Enriched and/or purified peptides were resuspended in 50 μl of water with 0.1% formic acid (v/v). A total of 10 μl of sample was loaded and washed for 5 min on a C_18_-trap column 0.3 × 5 mm, 5 μm, 100 Å, with a solvent of A:B = 96:4 (A, 0.1% 3 formic acid aqueous solution; B, 0.1% formic acid acetonitrile solution) at a constant flow of 0.03 μl/min. Peptides were transferred onward to an in house C_18_-packed analytical column (15 cm × 100 μm, 5 μm, 200 Å) and separated with a linear gradient of 5 to 35 min, 6 to 36% B, 36 to 38 min, 36 to 75% B, 38 to 60 min, 6% B (v/v) at the flow rate of 300 nl/min. The Orbitrap was operated under data-dependent acquisition mode. The spray voltage and capillary temperature were set to 1.8 kV and 325 °C, respectively. Full-scan mass spectra were acquired in Orbitrap over 350 to 1200 *m/z* with a resolution of 120,000, followed by MS^n^ scans by HCD activation mode. Data-dependent MS^2^ scan was carried out with cycle time 3s, isolation window (*m/z*) 1.6, HCD collision energy (%) 35, Orbitrap resolution 15,000, maximum injection time 40 ms, and AGC target (%) 100. Charge state 2 to 6 (charge state 1 was rejected) as well as dynamic exclusion duration 40 s was enabled.

### Proteomic data analysis

LC-FT/MS raw data were searched by Proteome Discoverer 2.5 (PD 2.5, Thermo Fisher Scientific) using SEQUEST search engine (Thermo Fisher Scientific), against the Uniprot Human database (11/26/2019, 140705 sequences) at a false discovery cut off of ≤1%. A maximum of two missed cleavage sites was allowed with trypsin full cleavage. The mass tolerance for the precursor ion was 30 ppm and for the fragment was 0.1 Da. Phosphorylation of serine, threonine, and tyrosine residues were enabled as dynamic modifications, while carbamidomethylation of cysteine was set as fixed modification. Filter settings including minimum peptide length = 6, peptide modification site probability threshold = 75, and peptide-spectrum matches with a delta Cn value = 0.05 were employed.

### Biological function and pathway analysis

NP-bound proteins were uploaded to IPA (Ingenuity Systems) server for a Core analysis to investigate the protein function and biological networks. Canonical pathway analysis was also carried out.

### NanoBiT assay

HEK293T cells were maintained in Dulbecco's modified Eagle's medium supplemented with 10% FBS and 1% penicillin and streptomycin antibiotic solution. Cells were plated in 96-well white/clear bottom culture plates with 40% confluence and transient transfection was performed with the indicated constructs (1:1 ratio of interacting pairs) using Lipofectamine 3000 (Invitrogen) in OPTI-MEM according to the manufacturers' instructions. Twenty-four hours post transfection, cells were treated with serial concentrations (1.3–14 μM) of 1E7-03 for an additional 6 h. Nano-Glo Live Cell Substrate (N2012, Promega) was added, and luminescence was measured using a GloMax-Multi Detection System (Promega). All experiments were performed at least three times.

### Western blotting

To test NanoBiT construct expression, cells were lysed in whole cell lysis buffer (50 mM Tris–HCl, pH 7.5, 0.5 M NaCl, 1% NP-40, and 0.1% SDS) supplemented with protease and phosphatase cocktail and separated on a 10% polyacrylamide gel, transferred to polyvinylidene difluoride membranes (Millipore). Protein bands were detected with indicated NP (IBT Bioservices) and PP1 (Upstate) antibodies using horseradish peroxidase–linked secondary antibodies.

### Co-immunoprecipitation

GFP-tagged NP-WT and NP E619K vectors were cotransfected with FLAG- or V5-tagged PP1α in HEK293T cells. Twenty-four hours post-transfection, the cells were treated with DMSO or 1E7-03 (10 μM) overnight. The protein lysate was prepared using a whole cell lysis buffer supplemented with a protease and phosphatase cocktail. Protein extract (250 μg) was supplemented with 1.5 μg of an anti-FLAG (Sigma) or 2.0 μg of anti-V5 (Invitrogen) antibodies and incubated with pre-blocked protein A/G-agarose beads in 5% bovine serum albumin in TNN buffer (50 mM Tris–HCl, pH 7.5, 0.5% NP-40, 150 mM NaCl) for 4 h with rocking. After washing with TNN buffer, proteins precipitated with the agarose beads were resolved on 10% Bis-Tris SDS-PAGE, transferred to polyvinylidene difluoride membrane, and probed with indicated antibodies.

### DSP cross-linking

HEK293T cells were transfected with the WT NP or NP E619K expressing plasmids. Twenty-four hours post transfection, the cells were treated with DMSO or 1E7-03 (10 μM) for overnight. Then, the cells were incubated with 2 mM DSP cross-linker (Thermo Fisher Scientific) for 45 min at room temperature. The cells were then lysed in the modified whole cell lysis buffer resuspended in a nonreducing sample buffer and subjected to SDS-PAGE and immunoblot analysis.

### Transmission electron microscopy

To analyze the NP-mediated capsid formation, HEK293 cells were transfected with vectors expressing NP-mCherry (WT or E619K mutant) alone or in combination with VP24- and VP35-expressing vectors. The cells were additionally treated with 3 μM 1E7-03 starting at 18 h after the transfection. At 48 h post transfection, the cells were incubated in a warm fixative solution (120 mM sodium cacodylate pH 7.4, 2.5% glutaraldehyde, 1% paraformaldehyde) for 20 min at room temperature and stored at 4 °C overnight. Cells were then osmicated by incubating 120 mM sodium cacodylate pH 7.4 supplemented with 1% OsO_4_ for 1 h and then overnight in water solution of 1% uranyl acetate at 4 °C. The cells were then dehydrated through the series of EtOH dilutions from 30% to 100%, embedded in LX112 epoxy resin and heated at 60 °C for 48 h. Sample blocks were sectioned *on face* and poststained with 1% uranyl acetate in water and Reynold's lead. All imaging was performed at 80 KV in a Talos 200X TEM (Thermo Fisher Scientific). In a typical experiment, 5 to 10 various images were captured for each condition.

### Fluorescent microscopy

To analyze the NP interaction with PP1, HEK293T cells were transfected with vectors expressing NP-mCherry in combination of PP1α-eGFP, PP1β/δ-eGFP, or PP1γ-eGFP (45). At 24 h post transfection, the cells were treated with Hoechst and photographed on Olympus IX73 (Olympus) using filters for DAPI, Texas Red, and FITC fluorescence with 600X magnification. Colocalization was quantified using Manders coefficient which is defined as MOC = ∑i(Ri × Gi)/√(∑iR2i × ∑iG2i) where Ri and Gi are the average level of gray from the red and green fluorescence, respectively (46). Manders coefficient was calculated in ImageJ using the JACoP plug-in.

### Flow cytometry analysis

HEK293 cells were cotransfected with either NP-mCherry, VP24, and VP35 (3:1:1 ratio) plasmids or with NP E619K-mCherry, VP24, and VP35 plasmids using Lipofectamine Plus reagent (Thermo Fisher Scientific). Transfected cells were treated with either 1E7-03 (3 μM) or DMSO for overnight. Cells were treated with trypsin, washed with 1 ml of PBS, resuspended in 0.5 ml of PBS, and analyzed using FACSVerse (Becton Dickinson). Flow cytometry analysis was conducted in triplicates.

### AlphaFold 3 use for NP molecular presentations

AlphaFold 3 (24) (https://alphafoldserver.com) was used to predict complexes of PP1 with NP, NP dimers, and NP–NP–PP1 trimers. Chimera X 1.3 (https://www.cgl.ucsf.edu/chimera) was used to visualize the structures built by AlphaFold 3. The following RNA sequence was obtained from the complete genome of Yambuku-Mayinga strain of Zaire EBOV isolate (NC_002549.1) and used for building NP dimers with RNA models: CGGACACACA AAAAGAAAGA AGAAUUUUUA GGAUCUUUUG UGUGCGAAUA ACUAUGAGGA AGAUUAAUAA UUUUCCUCUC AUUGAAAUUU AUAUCGGAAU UUAAAUUGAA. The following NP sequence Yambuku-Mayinga strain of Zaire EBOV isolate was used for AlphaFold 3 models: MDSRPQKIWMAPSLTESDMDYHKILTAGLSVQQGIVRQRVIPVYQVNNLEEICQLIIQAFEAGVDFQESA DSFLLMLCLH HAYQGDYKLF LESGAVKYLE GHGFRFEVKK RDGVKRLEELLPAVSSGKNI KRTLAAMPEE ETTEANAGQF LSFASLFLPK LVVGEKACLE KVQRQIQVHAEQGLIQYPTA WQSVGHMMVI FRLMRTNFLI KFLLIHQGMH MVAGHDANDA VISNSVAQARFSGLLIVKTV LDHILQKTER GVRLHPLART AKVKNEVNSF KAALSSLAKH GEYAPFARLLNLSGVNNLEH GLFPQLSAIA LGVATAHGST LAGVNVGEQY QQLREAATEA EKQLQQYAESRELDHLGLDD QEKKILMNFH QKKNEISFQQ TNAMVTLRKE RLAKLTEAIT AASLPKTSGHYDDDDDIPFP GPINDDDNPG HQDDDPTDSQ DTTIPDVVVD PDDGSYGEYQ SYSENGMNAPDDLVLFDLDE DDEDTKPVPN RSTKGGQQKN SQKGQHIEGR QTQSRPIQNV PGPHRTIHHASAPLTDNDRR NEPSGSTSPR MLTPINEEAD PLDDADDETS SLPPLESDDE EQDRDGTSNRTPTVAPPAPV YRDHSEKKEL PQDEQQDQDH TQEARNQDSD NTQSEHSFEE MYRHILRSQGPFDAVLYYHM MKDEPVVFST SDGKEYTYPD SLEEEYPPWL TEKEAMNEEN RFVTLDGQQFYWPVMNHKNK FMAILQHHQ. The following PP1 sequence (NP_001003064.2) was used for AlphaFold 3 models:

MSDSEKLNLDSIIGRLLEVQGSRPGKNVQLTENEIRGLCLKSREIFLSQPILLELEAPLKICGDIHGQYY DLLRLFEYGG FPPESNYLFL GDYVDRGKQS LETICLLLAY KIKYPENFFL LRGNHECASI NRIYGFYDEC KRRYNIKLWK TFTDCFNCLP IAAIVDEKIF CCHGGLSPDL QSMEQIRRIM RPTDVPDQGL LCDLLWSDPD KDVQGWGEND RGVSFTFGAE VVAKFLHKHD LDLICRAHQV VEDGYEFFAK RQLVTLFSAP NYCGEFDNAG AMMSVDETLM CSFQILKPAD KNKGKYGQFS GLNPGGRPIT PPRNSAKAKK.

### Meme prediction of PP1-binding sites in NP

We used the MEME program to determine PP1-binding sites within NP sequence (https://meme-suite.org/meme/tools/meme). This computational tool identifies motifs in a group of related DNA or protein sequences. The MEME algorithm uses a probabilistic model called EM to find patterns that are statistically significant across the input sequences. We used Find Individual Motif Occurrences to scan NP sequence for RVxF motif (27) defined as [KRPILG][V]x[FW] and *p*-value set as *p* < 0.01. We also scanned NP sequence for an alternative RVxF motif (28) defined as [F]xx[KR]x[KR] with *p*-value set as *p* < 0.01. These two searches identified four top motifs with q-value <0.4, that included ^63^GVDF^66^, ^106^FEVKKR^111^, ^483^LVLF^486^, and ^675^PVVF^678^.

### Per-residue disorder prediction and functional disorder analysis

The per-residue intrinsic disorder propensity of the Ebola NP was evaluated using a set of commonly used per-residue disorder predictors, such as PONDR VLS2 (47, 48), PONDR VL3 (49), PONDR VLXT (50), PONDR FIT (51), as well as IUPred-Long and IUPred-Short (52). A web-based platform, Rapid Intrinsic Disorder Analysis Online, was used to gather results from these predictors and to generate a mean disorder profile by averaging the outputs of individual predictors (53). In these analyses, the disorder score was assigned to each residue, with a residue with disorder score ≥ 0.5 being considered as disordered and a residue with disorder score < 0.5 being predicted as ordered. Residues/regions with disorder scores between 0.15 and 0.5 were considered as ordered but flexible. Rapid Intrinsic Disorder Analysis Online also can be used to calculate the percentage of predicted intrinsically disordered residues (PPIDR) in a query protein. This measure can be used for classification of proteins as ordered (PPIDR < 10%), moderately disordered (10% ≤ PPIDR < 30%), and highly disordered (PPIDR ≥ 30%) (54). The presence of the molecular recognition features (which are disordered regions that can undergo binding-induced folding at interaction with specific partners) in the Ebola NP was evaluated using the IUPred2A platform (52).

### Statistical analysis

All graphs were prepared using GraphPad Prism 10.4.1 software. The data were presented as mean ± SD or SEM as indicated in the figure legends. Statistical comparison was done with Student's *t* test. Where indicated, nonlinear regression analysis was performed to determine IC_50_ using GraphPad Prism 6 built-in algorithms.

## Data availability

The mass spectrometry raw data and Proteome Discoverer processed files are uploaded to PRIDE database (reference: 1-20240823-063919-3403965). All vectors generated during this study and original TEM images are available upon request from corresponding authors.

## Supporting information

This article contains supporting information.

## Conflicts of interests

The authors declare that they have no conflicts of interests with the contents of this article.

## References

[1] Feldmann H., Sprecher A., Geisbert T.W. (2020). Ebola N. Engl. J. Med..

[2] Agua-Agum J., Allegranzi B., Ariyarajah A., Aylward R., Blake I.M., WHO Ebola Response Team (2016). After ebola in West Africa--unpredictable risks, preventable epidemics. N. Engl. J. Med..

[3] Santoni de Sio F.R., Massacand J., Barde I., Offner S., Corsinotti A., Kapopoulou A. (2012). KAP1 regulates gene networks controlling mouse B-lymphoid cell differentiation and function. Blood.

[4] Kuhn J.H. (2008).

[5] Ilinykh P.A., Tigabu B., Ivanov A., Ammosova T., Obukhov Y., Garron T. (2014). Role of protein phosphatase 1 in dephosphorylation of Ebola virus VP30 protein and its targeting for the inhibition of viral transcription. J. Biol. Chem..

[6] Kruse T., Biedenkopf N., Hertz E.P.T., Dietzel E., Stalmann G., Lopez-Mendez B. (2018). The ebola virus nucleoprotein recruits the host PP2A-B56 phosphatase to activate transcriptional support activity of VP30. Mol. Cell.

[7] Takamatsu Y., Krahling V., Kolesnikova L., Halwe S., Lier C., Baumeister S. (2020). Serine-arginine protein kinase 1 regulates Ebola Virus transcription. mBio.

[8] Bollen M., Peti W., Ragusa M.J., Beullens M. (2010). The extended PP1 toolkit: designed to create specificity. Trends Biochem. Sci..

[9] Peti W., Nairn A.C., Page R. (2013). Structural basis for protein phosphatase 1 regulation and specificity. FEBS J..

[10] Peti W., Page R. (2015). Strategies to make protein serine/threonine (PP1, calcineurin) and tyrosine phosphatases (PTP1B) druggable: achieving specificity by targeting substrate and regulatory protein interaction sites. Bioorg. Med. Chem..

[11] Choy M.S., Hieke M., Kumar G.S., Lewis G.R., Gonzalez-DeWhitt K.R., Kessler R.P. (2014). Understanding the antagonism of retinoblastoma protein dephosphorylation by PNUTS provides insights into the PP1 regulatory code. Proc. Natl. Acad. Sci. U. S. A.

[12] Ammosova T., Platonov M., Yedavalli V.R., Obukhov Y., Gordeuk V.R., Jeang K.T. (2012). Small molecules targeted to a non-catalytic “RVxF” binding site of protein phosphatase-1 inhibit HIV-1. PLoS One.

[13] Ammosova T., Platonov M., Ivanov A., Kont Y.S., Kumari N., Kehn-Hall K. (2014). 1E7-03, a low MW compound targeting host protein phosphatase-1, inhibits HIV-1 transcription. Br. J. Pharmacol..

[14] Lin X., Kumari N., DeMarino C., Kont Y.S., Ammosova T., Kulkarni A. (2017). Inhibition of HIV-1 infection in humanized mice and metabolic stability of protein phosphatase-1-targeting small molecule 1E7-03. Oncotarget.

[15] Jerebtsova M., Ahmad A., Kumari N., Rutagarama O., Nekhai S. (2020). Macrophage HIV-1 gene expression and delay resolution of inflammation in HIV-Tg mice. Viruses.

[16] Jerebtsova M., Ahmad A., Niu X., Rutagarama O., Nekhai S. (2020). HIV-1 transcription inhibitor 1E7-03 Restores LPS-induced alteration of lung leukocytes' Infiltration dynamics and resolves inflammation in HIV transgenic mice. Viruses.

[17] Tigabu B., Ramanathan P., Ivanov A., Lin X., Ilinykh P.A., Parry C.S. (2018). Phosphorylated Vp30 of Marburg virus is a repressor of transcription. J Virol..

[18] Baer A., Shafagati N., Benedict A., Ammosova T., Ivanov A., Hakami R.M. (2016). Protein Phosphatase-1 regulates Rift Valley fever virus replication. Antivir. Res.

[19] Bracci N., Baer A., Flor R., Petraccione K., Stocker T., Zhou W. (2024). CK1 and PP1 regulate Rift Valley fever virus genome replication through L protein phosphorylation. Antivir. Res.

[20] Carey B.D., Ammosova T., Pinkham C., Lin X., Zhou W., Liotta L.A. (2018). Protein phosphatase 1alpha interacts with Venezuelan equine encephalitis virus capsid protein and regulates viral replication through modulation of capsid phosphorylation. J. Virol..

[21] Ammosova T., Pietzsch C.A., Saygideger Y., Ilatovsky A., Lin X., Ivanov A. (2018). Protein phosphatase 1-targeting small-molecule C31 inhibits ebola virus replication. J. Infect. Dis..

[22] Lin X., Ammosova T., Choy M.S., Pietzsch C.A., Ivanov A., Ahmad A. (2019). Targeting the non-catalytic RVxF site of protein phosphatase-1 with small molecules for ebola virus inhibition. Front Microbiol..

[23] Towner J.S., Paragas J., Dover J.E., Gupta M., Goldsmith C.S., Huggins J.W. (2005). Generation of eGFP expressing recombinant Zaire ebolavirus for analysis of early pathogenesis events and high-throughput antiviral drug screening. Virology.

[24] Abramson J., Adler J., Dunger J., Evans R., Green T., Pritzel A. (2024). Accurate structure prediction of biomolecular interactions with AlphaFold 3. Nature.

[25] Kirchdoerfer R.N., Saphire E.O., Ward A.B. (2019). Cryo-EM structure of the Ebola virus nucleoprotein-RNA complex Acta crystallographica Section F. Struct. Biol. Commun..

[26] Reid S.P., Cardenas W.B., Basler C.F. (2005). Homo-oligomerization facilitates the interferon-antagonist activity of the ebolavirus VP35 protein. Virology.

[27] Hendrickx A., Beullens M., Ceulemans H., Den Abt T., Van Eynde A., Nicolaescu E. (2009). Docking motif-guided mapping of the interactome of protein phosphatase-1. Chem. Biol..

[28] Garcia A., Cayla X., Caudron B., Deveaud E., Roncal F., Rebollo A. (2004). New insights in protein phosphorylation: a signature for protein phosphatase 1 interacting proteins. C R Biol..

[29] Kirchdoerfer R.N., Abelson D.M., Li S., Wood M.R., Saphire E.O. (2015). Assembly of the ebola virus nucleoprotein from a chaperoned VP35 Complex. Cell Rep.

[30] Prevost M., Chamousset D., Nasa I., Freele E., Morrice N., Moorhead G. (2013). Quantitative fragmentome mapping reveals novel, domain-specific partners for the modular protein RepoMan (recruits PP1 onto mitotic chromatin at anaphase). Mol. Cell. Proteomics.

[31] Qian J., Beullens M., Huang J., De Munter S., Lesage B., Bollen M. (2015). Cdk1 orders mitotic events through coordination of a chromosome-associated phosphatase switch. Nat. Commun..

[32] Kumar G.S., Gokhan E., De Munter S., Bollen M., Vagnarelli P., Peti W. (2016). The Ki-67 and RepoMan mitotic phosphatases assemble via an identical yet novel mechanism. eLife.

[33] Wurzenberger C., Held M., Lampson M.A., Poser I., Hyman A.A., Gerlich D.W. (2012). Sds22 and Repo-Man stabilize chromosome segregation by counteracting Aurora B on anaphase kinetochores. J. Cell Biol..

[34] Vagnarelli P., Ribeiro S., Sennels L., Sanchez-Pulido L., de Lima Alves F., Verheyen T. (2011). Repo-Man coordinates chromosomal reorganization with nuclear envelope reassembly during mitotic exit. Dev. Cell.

[35] de Castro I.J., Budzak J., Di Giacinto M.L., Ligammari L., Gokhan E., Spanos C. (2017). Repo-Man/PP1 regulates heterochromatin formation in interphase. Nat. Commun..

[36] Watanabe S., Noda T., Kawaoka Y. (2006). Functional mapping of the nucleoprotein of Ebola virus. J. Virol..

[37] Ivanov A., Ramanathan P., Parry C., Ilinykh P.A., Lin X., Petukhov M. (2020). Global phosphoproteomic analysis of Ebola virions reveals a novel role for VP35 phosphorylation-dependent regulation of genome transcription. Cell Mol. Life Sci..

[38] Peyrol J., Thizon C., Gaillard J.C., Marchetti C., Armengaud J., Rollin-Genetet F. (2013). Multiple phosphorylable sites in the Zaire Ebolavirus nucleoprotein evidenced by high resolution tandem mass spectrometry. J. Virol. Methods.

[39] Dong S., Yang P., Li G., Liu B., Wang W., Liu X. (2015). Insight into the Ebola virus nucleocapsid assembly mechanism: crystal structure of Ebola virus nucleoprotein core domain at 1.8 Å resolution. Protein Cell.

[40] Leung D.W., Borek D., Luthra P., Binning J.M., Anantpadma M., Liu G. (2015). An intrinsically disordered peptide from ebola virus VP35 controls viral RNA synthesis by modulating nucleoprotein-RNA interactions. Cell Rep..

[41] Dziubanska P.J., Derewenda U., Ellena J.F., Engel D.A., Derewenda Z.S. (2014). The structure of the C-terminal domain of the Zaire ebolavirus nucleoprotein. Acta Crystallogr. D Biol. Crystallogr..

[42] Batra J., Hultquist J.F., Liu D., Shtanko O., Von Dollen J., Satkamp L. (2018). Protein interaction mapping identifies RBBP6 as a negative regulator of ebola virus replication. Cell.

[43] Batra J., Mori H., Small G.I., Anantpadma M., Shtanko O., Mishra N. (2021). Non-canonical proline-tyrosine interactions with multiple host proteins regulate Ebola virus infection. EMBO J..

[44] Thorvaldsdottir H., Robinson J.T., Mesirov J.P. (2013). Integrative Genomics Viewer (IGV): high-performance genomics data visualization and exploration. Brief. Bioinform..

[45] Ammosova T., Jerebtsova M., Beullens M., Lesage B., Jackson A., Kashanchi F. (2005). Nuclear targeting of protein phosphatase-1 by HIV-1 Tat protein. J. Biol. Chem..

[46] Manders E.M.M., Verbeek F.J., Aten J.A. (1993). Measurement of co-localization of objects in dual-colour confocal images. J. Microsc..

[47] Obradovic Z., Peng K., Vucetic S., Radivojac P., Dunker A.K. (2005). Exploiting heterogeneous sequence properties improves prediction of protein disorder. Proteins.

[48] Peng K., Radivojac P., Vucetic S., Dunker A.K., Obradovic Z. (2006). Length-dependent prediction of protein intrinsic disorder. BMC Bioinform..

[49] Peng K., Vucetic S., Radivojac P., Brown C.J., Dunker A.K., Obradovic Z. (2005). Optimizing long intrinsic disorder predictors with protein evolutionary information. J. Bioinform Comput. Biol..

[50] Romero P., Obradovic Z., Li X., Garner E.C., Brown C.J., Dunker A.K. (2001). Sequence complexity of disordered protein. Proteins.

[51] Xue B., Dunbrack R.L., Williams R.W., Dunker A.K., Uversky V.N. (2010). PONDR-FIT: a meta-predictor of intrinsically disordered amino acids. Biochim. Biophys. Acta.

[52] Meszaros B., Erdos G., Dosztanyi Z. (2018). IUPred2A: context-dependent prediction of protein disorder as a function of redox state and protein binding. Nucleic Acids Res..

[53] Dayhoff G.W., Uversky V.N. (2022). Rapid prediction and analysis of protein intrinsic disorder. Protein Sci..

[54] Rajagopalan K., Mooney S.M., Parekh N., Getzenberg R.H., Kulkarni P. (2011). A majority of the cancer/testis antigens are intrinsically disordered proteins. J. Cell Biochem..

