# Supplementary material for: Ebola virus nucleoprotein interaction with host protein phosphatase-1 regulates its dimerization and capsid formation
Source: J Biol Chem. 2025 Apr 25;301(6):108541. doi: 10.1016/j.jbc.2025.108541 (PMC12152876; doi:10.1016/j.jbc.2025.108541)
Supplement: Supplementary Figures and Tables [file mmc1.zip › jbc_108541_Supplemental Figures 051325_revised.pdf]

Supplemental Fig. S1

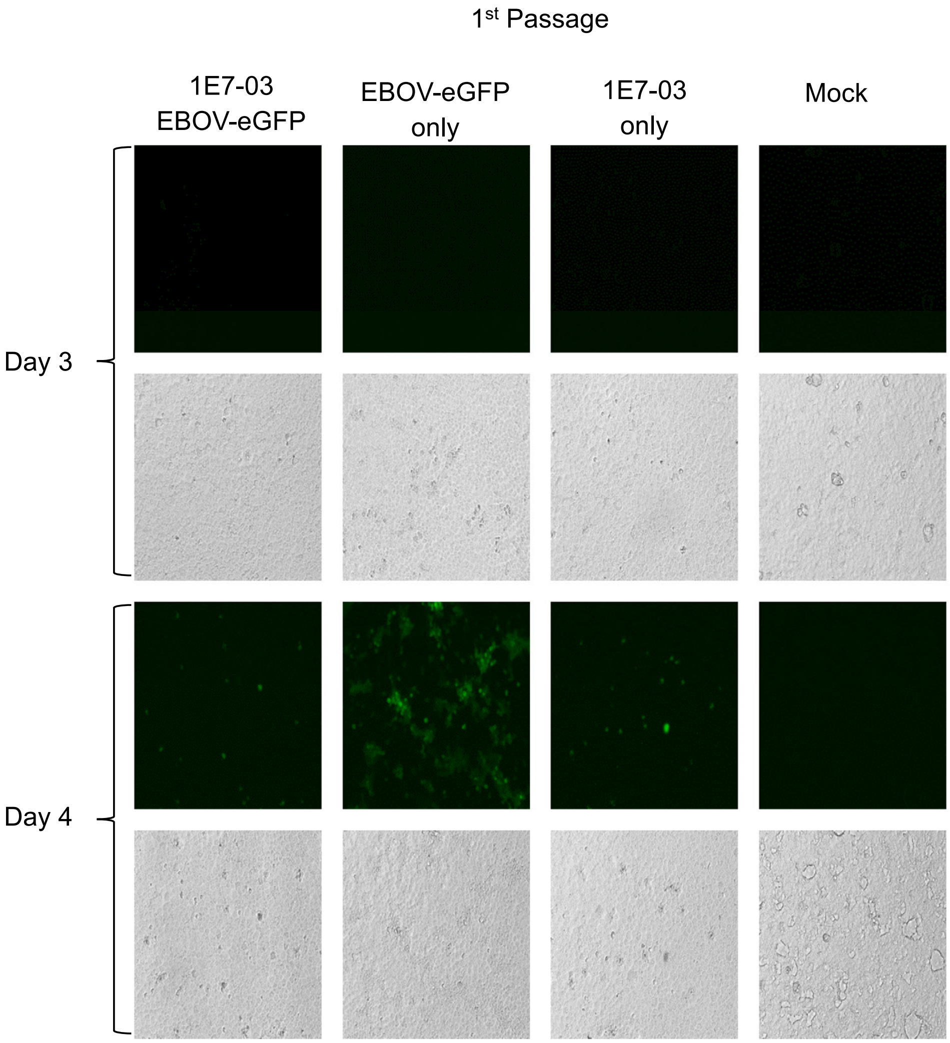


The GFP fluorescence of Vero-E6 cell monolayers at passage 1 on day 3 and day 4 post-infection. Non-infected cells were also treated with 3 μM 1E7-03 and DMSO (as a control).

Supplemental Fig. S2

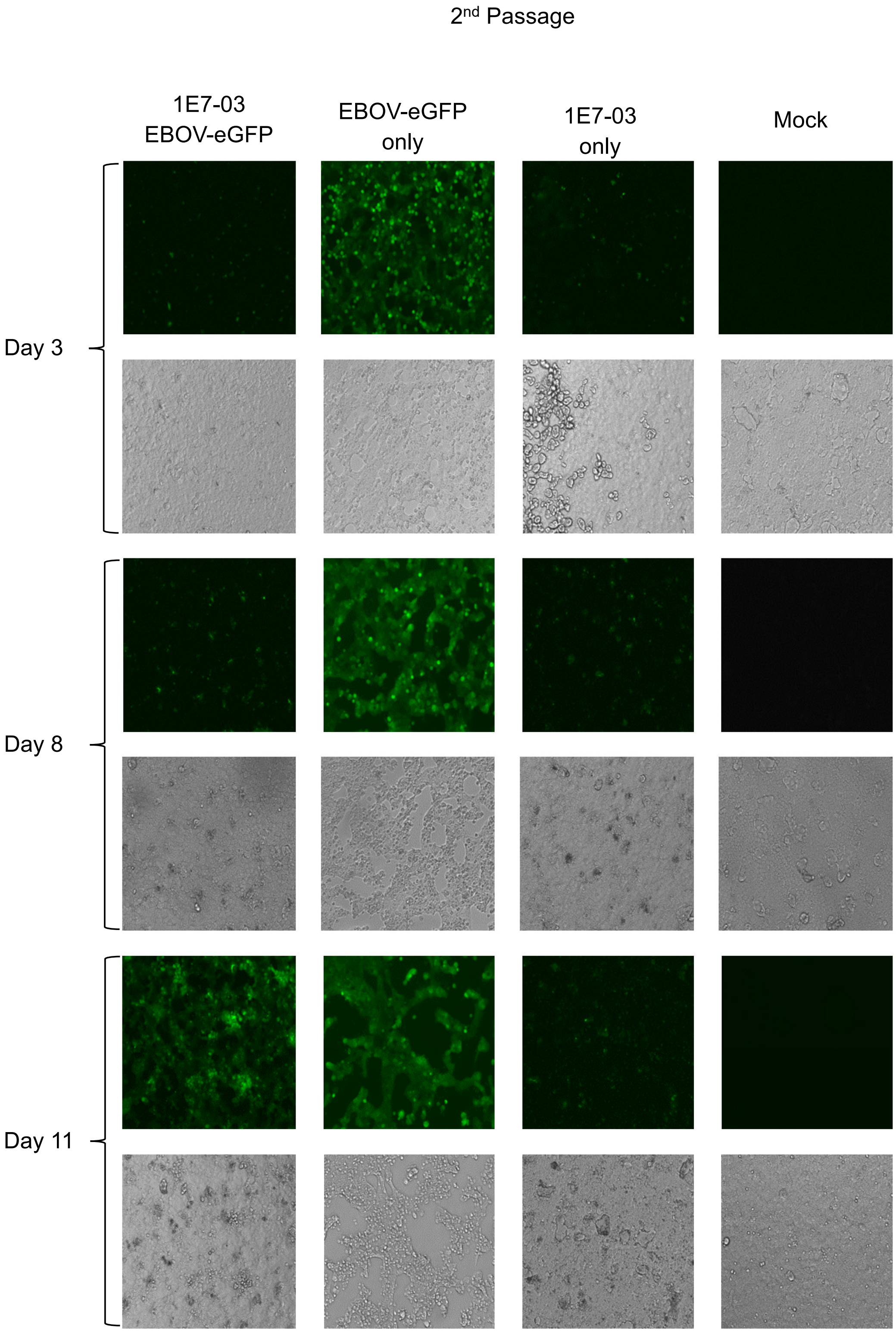


GFP fluorescence in Vero-E6 cell monolayers at passage 2 for day 3, day 8, and day 11 post-infection. Non-infected cells treated with 3 μM 1E7-03 and DMSO (mock) are also shown.

Supplemental Fig. S3

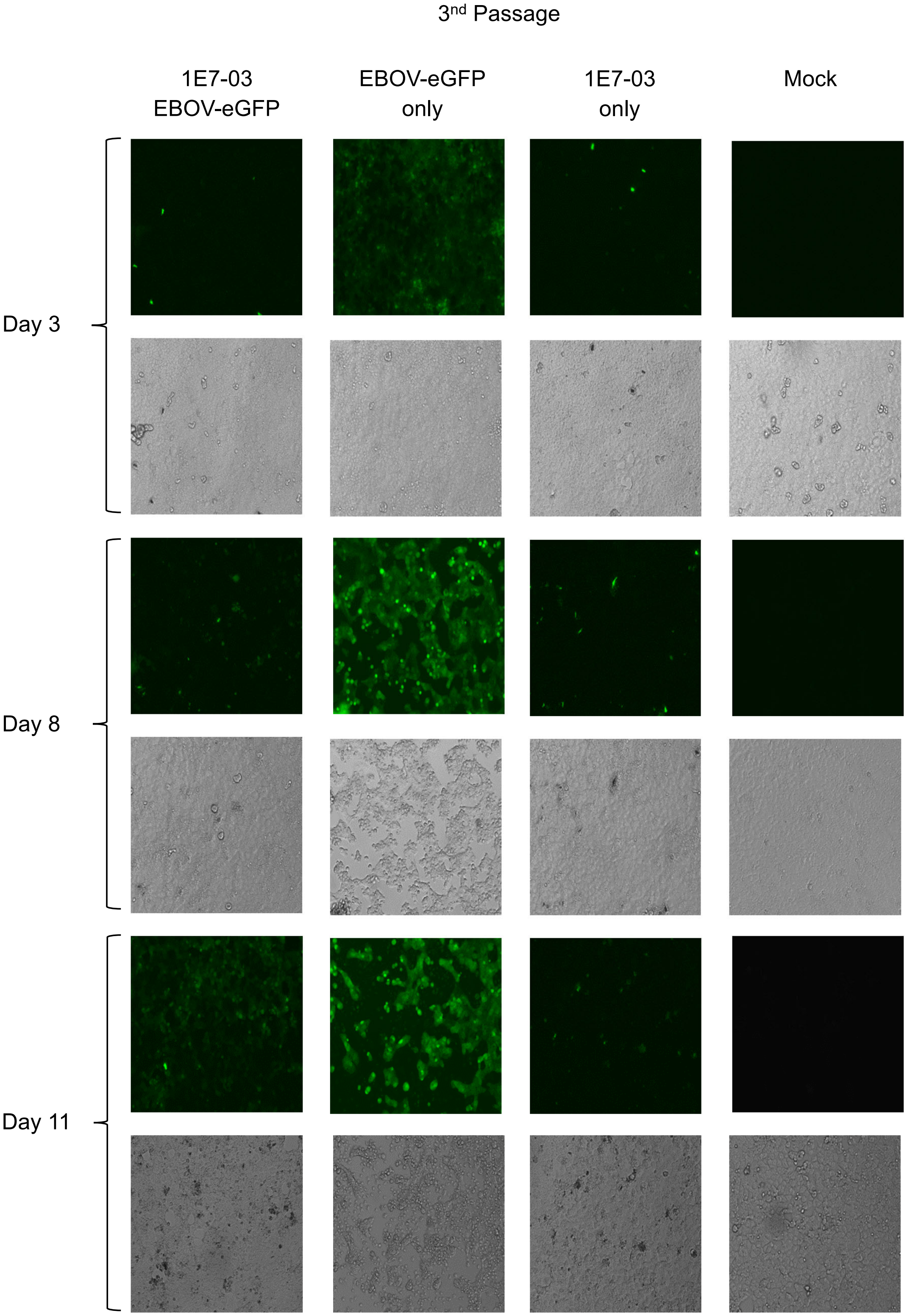


GFP fluorescence in Vero-E6 cell monolayers at passage 3 for day 3, day 8, and day 11 p.i. Non-infected cells treated with 3 μM 1E7-03 and DMSO (mock) are also shown.

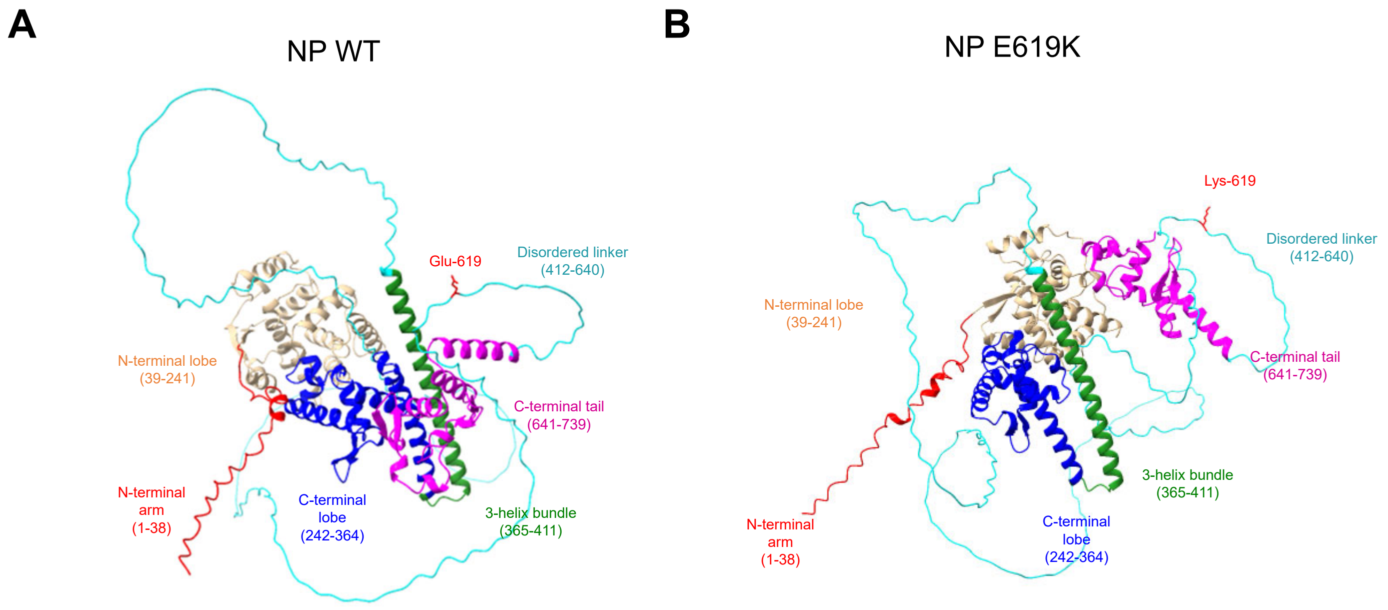


**Structures of NP and NP E619K.** AlphaFold 3 was used to build structures of NP (panel A) and E619K (panel B). NP's regions shown in color are N-terminal arm (red); N-terminal lobe (gold); C-terminal lobe (blue); 3 helix bundle (green); disordered inker (cyan) and C-termina tail (magenta). Position of Glu-619 (panels A) and Lys-619 (panels B) are shown in red with the side chains.

Supplemental Fig. S5

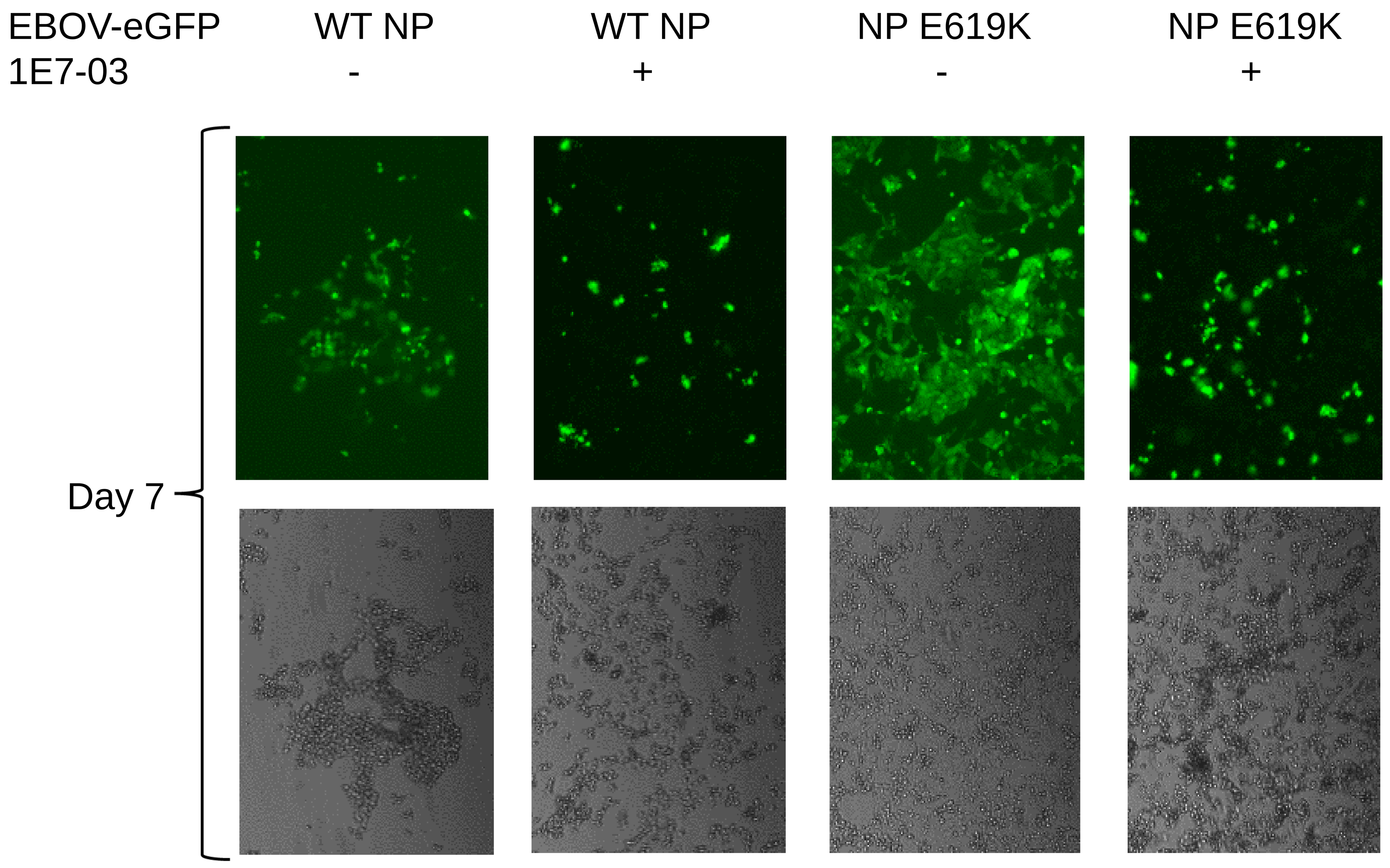


Expression of GFP in Vero-E6 cells infected with EBOV expressing WT NP or NP E619K mutant. GFP fluorescence at day 7 p.i. is shown. The cells treated with 3 µM 1E7-03 and DMSO (mock) are shown.

Supplemental Fig.S6

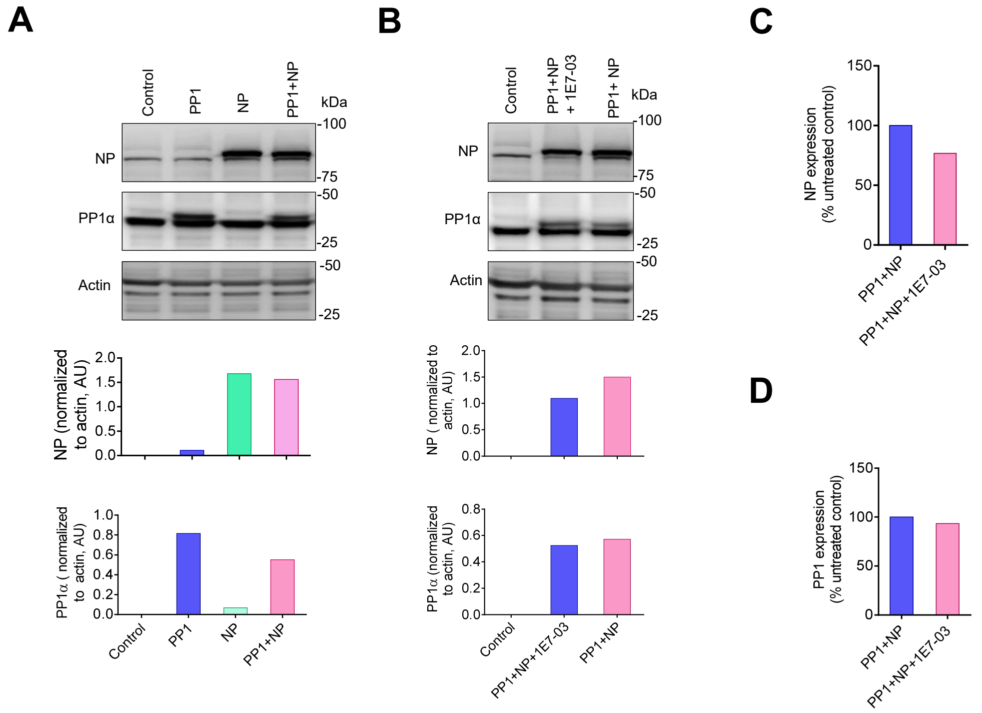


Expression of PP1 and NP in NanoBit complementation experiments. A, HEK293T cells were transfected with PP1-SmBit, NP-LgBit or their combination as indicated. At 30 hrs post-transfection, the cells were lysed, and the lysates were separated on SDS PAGE and immunoblotted with the antibodies against NP, PP1 and actin. B-D, Effect of 1E7-03 on the expression of PP1 and NP in Nanobit complementation assay. Cells were transfected with a combination of PP1SmBit and NPLgBit (1:1). At 24 hrs post transfection, cells were treated with 10 μM 1E7-03 for additional 6 hrs. Expression of NP and PP1 was determined by immunoblotting. In panels C and D, Expression of NP and PP1 are shown as present of DMSO as a control.

Fig. S7.

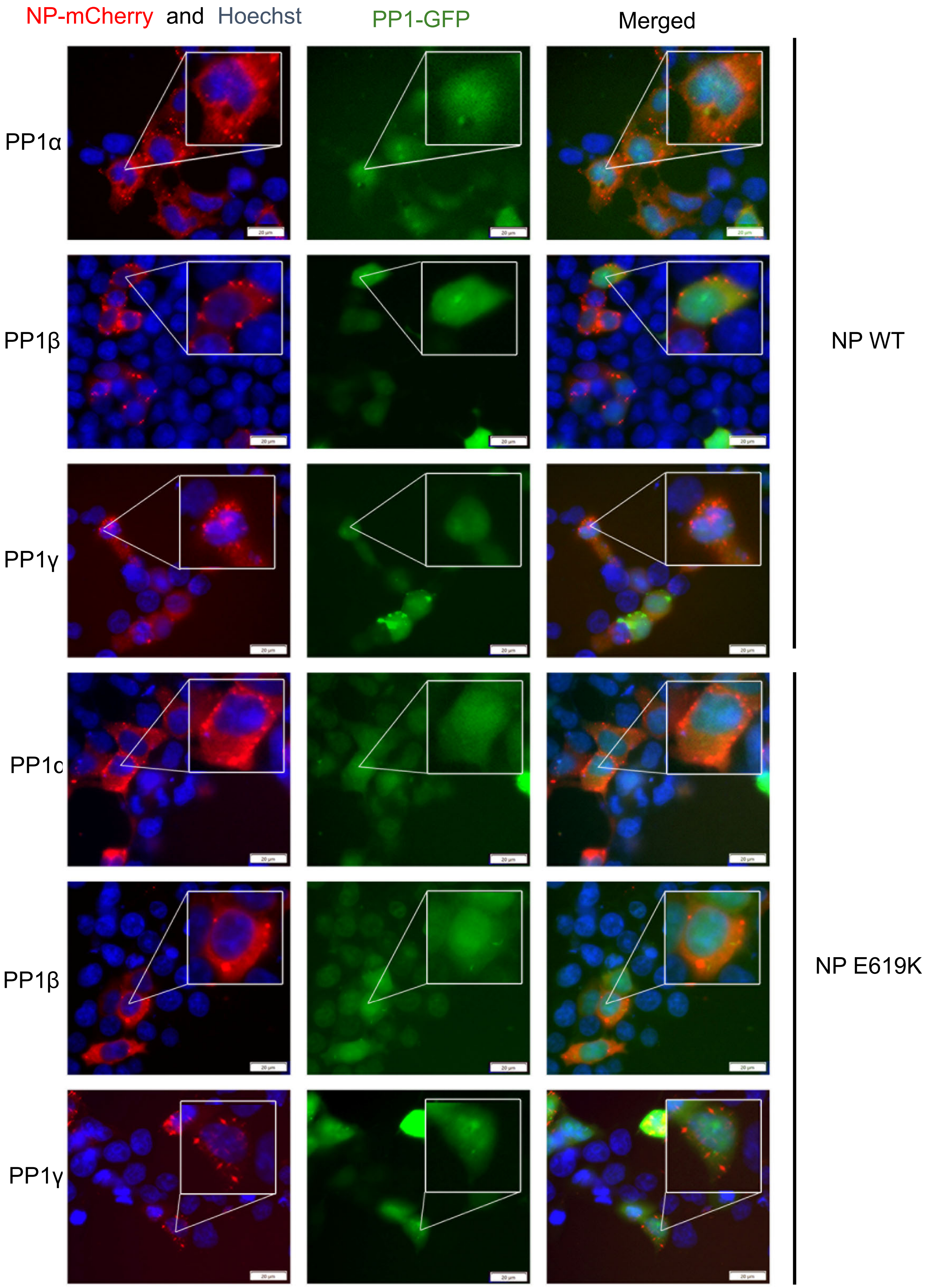


Fluorescent imaging of PP1 and NP co-localization. HEK293 cells were transfected with vectors expressing NP-mCherry and PP1(α, β/δ or γ)-GFP. At 48 h post transfection, the cells were stained with Hoechst 33342 and imaged at 600x magnification using filters for Texas Red, FITC and DAPI on Olympus IX73. The inserts show 2X expanded images of the selected cells. The original image has 20 μm scale.

Fig. S8. .

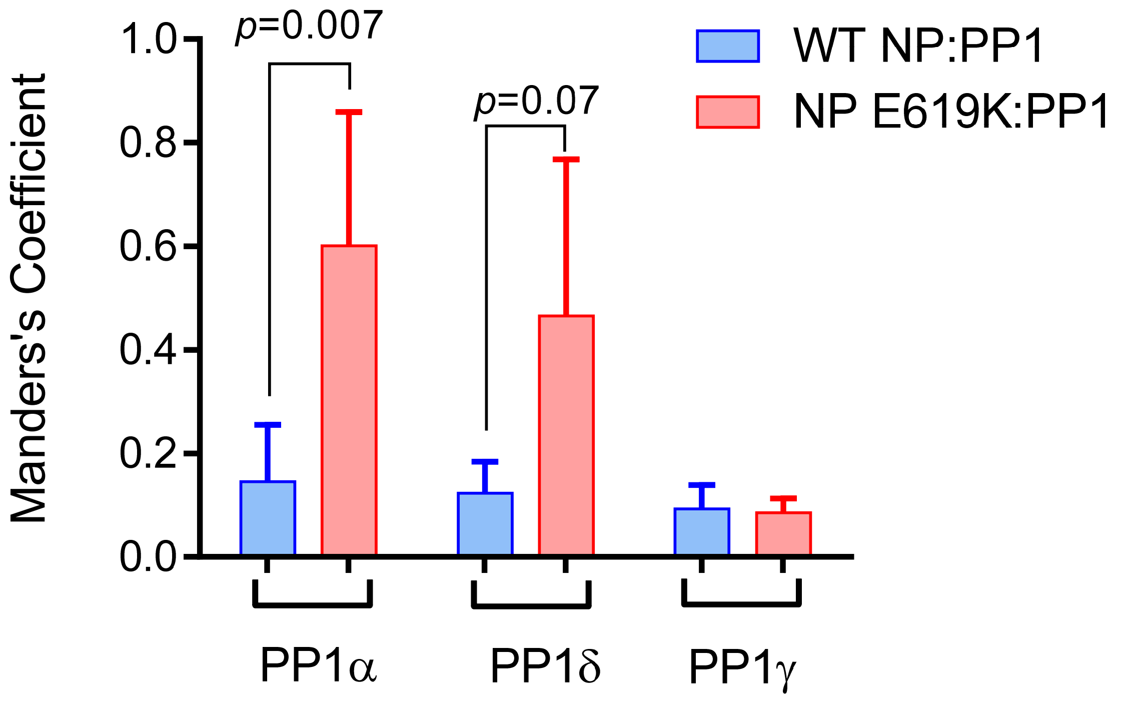


Quantification of PP1 and NP colocalization. The colocalization was assessed in Image J using the JACoP plug-in, which allows Mander's coefficient calculation. Prior to the analysis, the image colors were split, and the threshold parameters were adjusted.

Supplemental Fig. S9

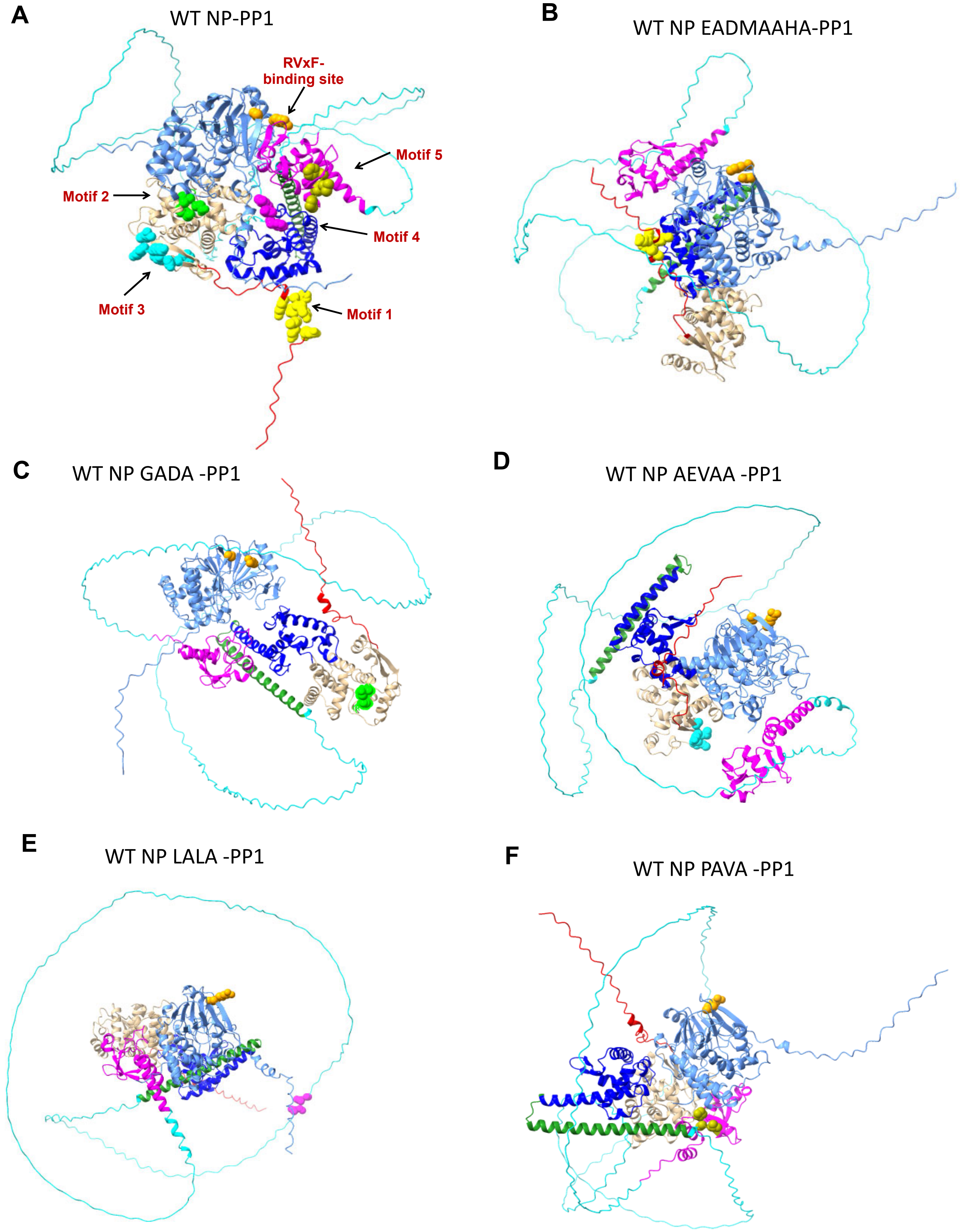


**Structures of PP1 and NP mutants.** AlphaFold 3 was used to build structures of PP1- NP with the indicated mutations. A, shown positions of motif 1 (yellow), Motif 2 (green), motif 3 (magenta), motif 4 (purple) and motif 5 (forest green). Panels B-F, AlphaFold 3 was used to build structures of PP1 with the mutated NP. Positions of mutated motifs are shown. PP1 is shown in cornflower blue. PP1's residues Arg262 and Cys291 are shown in orange as spheres to indicate the position of the RVxF-accommodating groove.

Supplemental Fig. S10

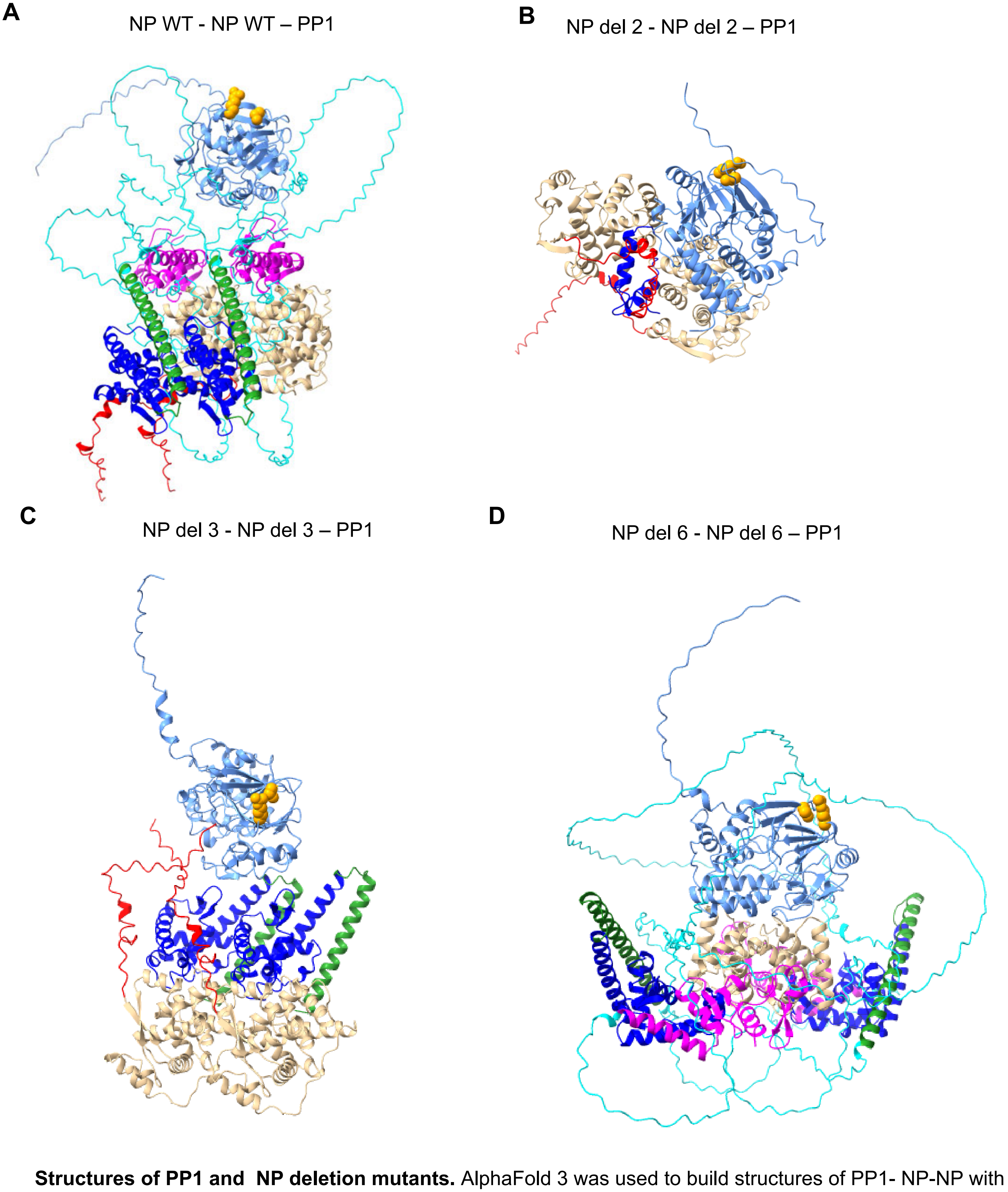


**Structures of PP1 and NP deletion mutants.** AlphaFold 3 was used to build structures of PP1- NP-NP with the indicated deletions. A, PP1-NP-NP structure. B, PP1-NP del2-NP del 2 structure. C, PP1-NP-del3-NP del3 structure. D, PP-1-NP-del 6-NP del 6 structure. NP's regions shown in color are N-terminal arm (red); N-terminal lobe (gold); C-terminal lobe (blue); 3 helix bundle (green); disordered inker (cyan) and C-termina tail (magenta). PP1 is shown in cornflower blue. PP1's residues Arg262 and Cys291 are shown in orange as spheres to indicate the position of the RVxF-accommodating groove.

Supplemental Fig. S11

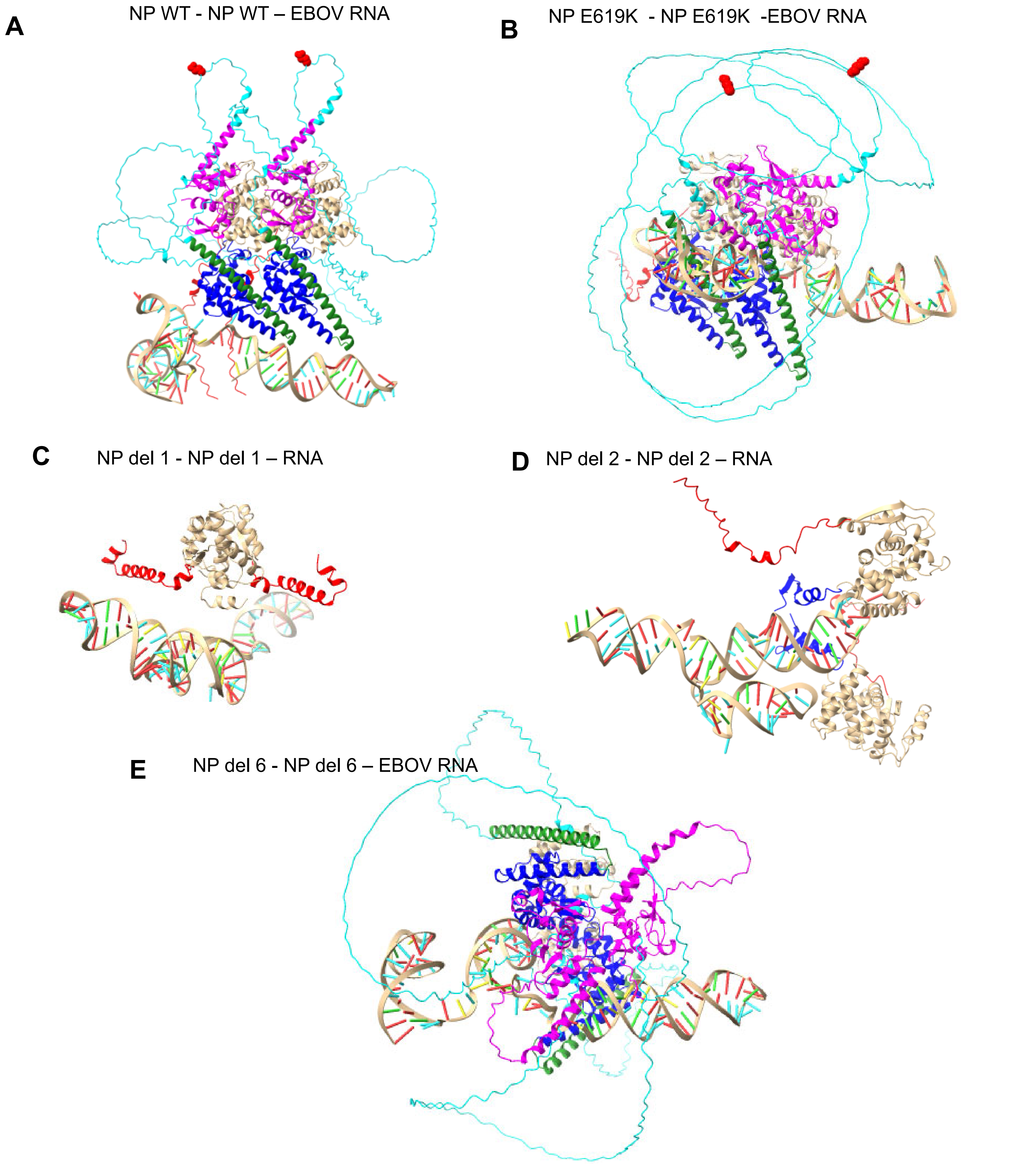


**RNA binding of NP deletion mutants.** AlphaFold 3 was used to build structures of NP dimers with the 110 nt EBOV RNA. A, NP-NP-RNA structure. Glu-619 position is shown as red spheres. B, NP E619K – NP E619K –RNA structure. Lys-619 position is shown as red spheres C, I2-NP del 2 structure. C, NP-del1-NP del1 – RNA structure. D, NP del2 – NP del2-RNA structure. E, NP-del 6-NP del 6 –RNA structure. NP's regions shown in color are N-terminal arm (red); N-terminal lobe (gold); C-terminal lobe (blue); 3 helix bundle (green); disordered inker (cyan) and C-termina tail (magenta). PP1 is shown in cornflower blue. PP1's residues Arg262 and Cys291 are shown in orange as spheres to indicate the position of the RVxF-accommodating groove.

Supplemental Fig. S12

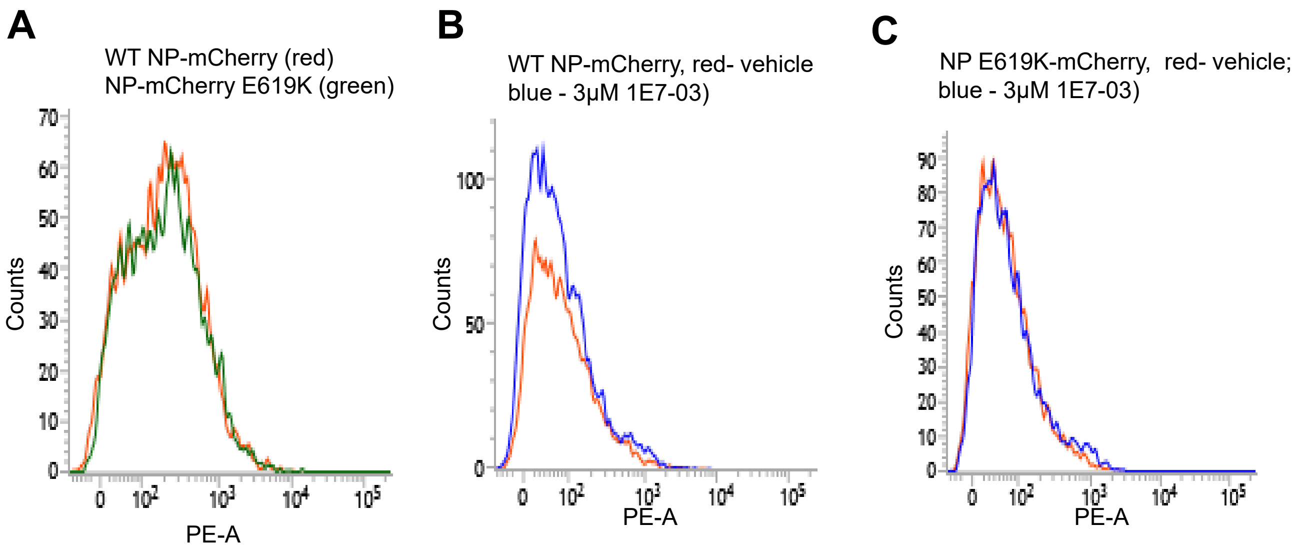


Flow cytometry analysis of NP and NP E619K mutant expression**.** 293 cells were transfected with vectors expressing NP-mCherry (WT or E619K mutant) in combination with VP35 and VP24. At 48 hours after transfection, the cells were analyzed by flow cytometry using FACS Versa (BD). A, expression of WT NP and NP E619K mutant. B and C, expression of WT NP or NP E619K mutant without or with 1E7-03 treatment.
