# Supplementary material for: Ebola virus nucleoprotein interaction with host protein phosphatase-1 regulates its dimerization and capsid formation
Source: J Biol Chem. 2025 Apr 25;301(6):108541. doi: 10.1016/j.jbc.2025.108541 (PMC12152876; doi:10.1016/j.jbc.2025.108541)
Supplement: Supplementary Figures and Tables [file mmc1.zip › jbc_108541_Supplementary Tables_051325_corrected.docx]

**Supplemental Table S1. EBOV genome sequencing from 1E7-03 treated samples.**

| **Sample 1, EBOV treated with 1E7-03** | | | | |
| --- | --- | --- | --- | --- |
| Gene | genome position | AA substitution | Frequency | P-value |
| NP | 614 | L49F | 0.93% | 6.21E-02 |
| NP | 1097 | I210V | 4.11% | 1.48E-02 |
| NP | 1954 | T495T | 1.96% | 5.01E-06 |
| 5’UTR VP40 | 4462 |  | 1.63% | 6.17E-02 |
| VP40 | 5057 | D193D | 7.50% | 3.23E-07 |
| VP40 | 5063 | T195T | 5.05% | 4.29E-04 |
| GP | 6063 | L9V | 21.74% | 3.66E-12 |
| GP | 7015 | T326T | 7.09% | 6.73E-07 |
| GP | 7016 | S327P | 6.34% | 2.89E-06 |
| GP | 7018 | S327P | 6.23% | 2.90E-06 |
| GP | 7582 | L515 F | 41.16% | 1.77E-46 |
| 5’UTR VP30 | 9450 |  | 4.07% | 3.0E-02 |
| VP24 | 10768 | K142E | 100.00% | 2.50E-30 |
| VP24 | 10769 | N142T | 100.00% | 2.50E-30 |
| L | 14703 | G1041G | 40.00% | 6.18E-05 |
| L | 16440 | P1620P | 50.00% | 3.91E-11 |
| L | 18042 | L2154F | 42.86% | 4.50E-06 |
| **Sample 2, EBOV treated with 1E7-03** | | | | |
| Position | genome position | AA substitution | Frequency | P-value |
| NP | 733 | K88K | 3.09% | 3.03E-02 |
| **NP** | **2324** | **E619K** | **8.91%** | **3.47E-22** |
| 5’UTR NP | 2913 |  | 2.49% | 9.23E-04 |
| 5’UTR NP | 2954 |  | 1.09% | 1.91E-03 |
| VP40 | 4893 | G193S | 0.95% | 3.10E-02 |
| VP40 | 5386 | L303P | 5.71% | 5.98E-02 |
| 5’UTR VP40 | 5759 |  | 2.88% | 6.11E-02 |
| GP | 6493 | A152V | 3.48% | 6.09E-02 |
| GP | 7018 | S327P | 4.17% | 3.00E-02 |
| GP | 7225 | L396L | 1.77% | 6.17E-02 |
| GP | 7248 | V404A | 3.44% | 1.82E-03 |
| GP | 7738 | Q567Q | 4.49% | 7.29E-03 |
| VP24 | 10768 | K142E | 100.00% | 1.39E-19 |
| VP24 | 10769 | N142T | 100.00% | 1.39E-19 |
| **Sample 3, EBOV treated with 1E7-03** | | | | |
| Position | genome position | AA substitution | Frequency | P-value |
| **NP** | **2324** | **E619K** | **5.56%** | **8.59E-04** |
| 5’UTR VP30 | 4303 |  | 8.51% | 5.85E-02 |

**Supplemental Table S2. EBOV genome sequencing from untreated cultures.**

| **Sample 1, EBOV untreated** | | | | | |
| --- | --- | --- | --- | --- | --- |
| Gene | genome position | AA substitution | Freq | | P-value |
|  | 2316 |  | 1.15% | | 3.78E-20 |
|  | 6671 |  | 4.67% | | 9.70E-90 |
|  | 6785 |  | 2.32% | | 3.03E-30 |
|  | 7564 |  | 1.62% | | 6.98E-27 |
|  | 8985 |  | 0.94% | | 1.07E-07 |
|  | 9703 |  | 2.07% | | 1.05E-22 |
| VP24 | 10768 | K142E | 99.64% | | 0.00E+00 |
| VP24 | 10769 | N142T | 99.91% | | 0.00E+00 |
|  | 15403 |  | 1.33% | | 5.98E-27 |
| **Sample 2, EBOV untreated** | | | | | |
| Position | genome position | AA substitution | Freq | | P-value |
|  | 2316 |  | 1.14% | | 3.14E-11 |
|  | 6671 |  | 3.94% | | 1.58E-46 |
|  | 6785 |  | 2.67% | | 3.10E-21 |
|  | 7564 |  | 2.22% | | 3.37E-21 |
|  | 8985 |  | 1.28% | | 1.40E-06 |
|  | 9703 |  | 1.73% | | 2.51E-13 |
| VP24 | 10768 | K142E | 99.75% | | 0.00E+00 |
| VP24 | 10769 | N142T | 99.90% | | 0.00E+00 |
|  | 11991 |  | 0.99% | | 1.28E-22 |
|  | 15403 |  | 0.95% | | 3.77E-10 |
| **Sample 3, EBOV untreated** | | | | | |
| Position | genome position | AA substitution | | Freq | P-value |
|  | 2316 |  | | 0.92% | 1.08E-13 |
|  | 6671 |  | | 3.64% | 3.19E-64 |
|  | 6785 |  | | 2.88% | 7.10E-42 |
|  | 7564 |  | | 1.35% | 1.81E-20 |
|  | 8985 |  | | 1.43% | 4.59E-09 |
|  | 9703 |  | | 1.62% | 1.22E-12 |
| VP24 | 10768 | K142E | | 99.95% | 0.00E+00 |
| VP24 | 10769 | N142T | | 99.93% | 0.00E+00 |

**Supplemental Table S3. Proteins bound to NP E619K versus WT NP analyzed by Proteome Discoverer 2.5.**

| Gene Symbol | Description | Normalized Abundance Ratio | Sequest score |
| --- | --- | --- | --- |
| EBOV NP | EBOV Flag-NP | 1 | 7022.09 |
| KRT6A | HCG2039812, isoform CRA_b (Fragment) OS=Homo sapiens OX=9606 GN=KRT6A PE=2 SV=1 | 1.771900826 | 3179.86 |
| H2AC20 | Histone H2A type 2-C OS=Homo sapiens OX=9606 GN=HIST2H2AC PE=1 SV=4 | 7.062809917 | 2813.48 |
| HIST1H2AC | Histone H2A OS=Homo sapiens OX=9606 GN=HIST1H2AC PE=3 SV=1 | 3.201652893 | 2764.11 |
| H2AZ2 | Histone H2A.V OS=Homo sapiens OX=9606 GN=H2AFV PE=1 SV=3 | 3.41322314 | 2668.87 |
| H2AX | Histone H2AX OS=Homo sapiens OX=9606 GN=H2AFX PE=1 SV=2 | 6.775206612 | 2509.5 |
| TOP2A | DNA topoisomerase 2-alpha OS=Homo sapiens OX=9606 GN=TOP2A PE=1 SV=3 | 2.72231405 | 2017.85 |
| HIST1H2BD | Histone H2B OS=Homo sapiens OX=9606 GN=HIST1H2BD PE=3 SV=1 | 3.571900826 | 1907.31 |
| H2BC18 | Histone H2B type 2-F OS=Homo sapiens OX=9606 GN=HIST2H2BF PE=1 SV=3 | 6.92892562 | 1897.66 |
| H2BC21 | Histone H2B type 2-E OS=Homo sapiens OX=9606 GN=HIST2H2BE PE=1 SV=3 | 3.651239669 | 1796.58 |
| H3C13; H3C14; H3C15 | Histone H3.2 OS=Homo sapiens OX=9606 GN=HIST2H3A PE=1 SV=3 | 9.932231405 | 1744.11 |
| H3C1; H3C10; H3C11; H3C12; H3C2; H3C3; H3C4; H3C6; H3C7; H3C8 | Histone H3.1 OS=Homo sapiens OX=9606 GN=HIST1H3A PE=1 SV=2 | 4.479338843 | 1732.51 |
| H2BC3 | Histone H2B type 1-B OS=Homo sapiens OX=9606 GN=HIST1H2BB PE=1 SV=2 | 6.983471074 | 1721.17 |
| H3-3A; H3-3B | Histone H3.3 OS=Homo sapiens OX=9606 GN=H3F3A PE=1 SV=2 | 4.834710744 | 1666.3 |
| MKI67 | Proliferation marker protein Ki-67 OS=Homo sapiens OX=9606 GN=MKI67 PE=1 SV=2 | 5.555371901 | 1665.75 |
| H3-7 | Histone H3 OS=Homo sapiens OX=9606 GN=HIST2H3PS2 PE=1 SV=1 | 3.687603306 | 1631.66 |
| H1-2 | Histone H1.2 OS=Homo sapiens OX=9606 GN=HIST1H1C PE=1 SV=2 | 1.923966942 | 1629.52 |
| H1-3 | Histone H1.3 OS=Homo sapiens OX=9606 GN=HIST1H1D PE=1 SV=2 | 3.517355372 | 1336.4 |
| PARP1 | Poly [ADP-ribose] polymerase OS=Homo sapiens OX=9606 GN=PARP1 PE=4 SV=1 | 3.495867769 | 1300.55 |
| TOP2B | DNA topoisomerase 2-beta OS=Homo sapiens OX=9606 GN=TOP2B PE=1 SV=3 | 2.608264463 | 1252.14 |
| HIST1H4J | Histone H4 OS=Homo sapiens OX=9606 GN=HIST1H4L PE=2 SV=1 | 3.403305785 | 1027.03 |
| HNRNPA2B1 | Heterogeneous nuclear ribonucleoproteins A2/B1 OS=Homo sapiens OX=9606 GN=HNRNPA2B1 PE=1 SV=2 | 2.241322314 | 861.89 |
| RPS27A | Ubiquitin-40S ribosomal protein S27a (Fragment) OS=Homo sapiens OX=9606 GN=RPS27A PE=1 SV=1 | 1.436363636 | 846.01 |
| UBA52 | Ubiquitin-60S ribosomal protein L40 (Fragment) OS=Homo sapiens OX=9606 GN=UBA52 PE=1 SV=1 | 2.310743802 | 617.12 |
| DDX3X | ATP-dependent RNA helicase DDX3X OS=Homo sapiens OX=9606 GN=DDX3X PE=1 SV=3 | 2.371900826 | 615.35 |
| ALB | Serum albumin OS=Homo sapiens OX=9606 GN=ALB PE=1 SV=2 | 1.381818182 | 613.11 |
| H1-6 | Histone H1t OS=Homo sapiens OX=9606 GN=HIST1H1T PE=2 SV=4 | 2.730578512 | 581.82 |
| TOP1 | DNA topoisomerase 1 OS=Homo sapiens OX=9606 GN=TOP1 PE=1 SV=2 | 2.943801653 | 572.04 |
| HEL-S-72p | Epididymis luminal protein 33 OS=Homo sapiens OX=9606 GN=HEL-S-72p PE=2 SV=1 | 1.763636364 | 565.12 |
| H2afy; H2AFY; LOC100466888; LOC101338325; LOC101381952; LOC101564704; LOC102987354; LOC103079324; LOC103663804; LOC105869993; LOC106989149; LOC110591142; LOC111141291; LOC111182462; LOC112404331; LOC112625777; LOC112815157; LOC112861289; LOC112917035; LOC113928700; LOC114891795; LOC115516119; LOC116529369; LOC116750637; MACROH2A1 | Core histone macro-H2A.1 OS=Homo sapiens OX=9606 GN=H2AFY PE=1 SV=4 | 1.161983471 | 548.87 |
| HNRNPU | Heterogeneous nuclear ribonucleoprotein U OS=Homo sapiens OX=9606 GN=HNRNPU PE=1 SV=6 | 1.791735537 | 520.21 |
| SRSF1 | Serine/arginine-rich-splicing factor 1 OS=Homo sapiens OX=9606 GN=SRSF1 PE=1 SV=1 | 1.573553719 | 487.58 |
| HNRNPA1 | Heterogeneous nuclear ribonucleoprotein A1 OS=Homo sapiens OX=9606 GN=HNRNPA1 PE=1 SV=2 | 1.633057851 | 478.02 |
| RPS3 | 40S ribosomal protein S3 OS=Homo sapiens OX=9606 GN=RPS3 PE=1 SV=2 | 0.791735537 | 459.42 |
| HNRNPU | Heterogeneous nuclear ribonucleoprotein U OS=Homo sapiens OX=9606 GN=HNRNPU PE=1 SV=9 | 2.148760331 | 452.87 |
| HNRNPC | Heterogeneous nuclear ribonucleoproteins C1/C2 OS=Homo sapiens OX=9606 GN=HNRNPC PE=1 SV=1 | 3.13553719 | 448.71 |
| SAFB | Scaffold attachment factor B1 OS=Homo sapiens OX=9606 GN=SAFB PE=1 SV=4 | 1.776859504 | 436.68 |
| HSPA1B | Heat shock 70 kDa protein 1B OS=Homo sapiens OX=9606 GN=HSPA1B PE=1 SV=1 | 1.819834711 | 423.43 |
| DHX9 | ATP-dependent RNA helicase A OS=Homo sapiens OX=9606 GN=DHX9 PE=1 SV=4 | 1.459504132 | 419.31 |
| MACROH2A2 | Core histone macro-H2A.2 OS=Homo sapiens OX=9606 GN=H2AFY2 PE=1 SV=3 | 1.201652893 | 408.66 |
| RPS18 | 40S ribosomal protein S18 OS=Homo sapiens OX=9606 GN=RPS18 PE=1 SV=3 | 2.234710744 | 394.86 |
| SUPT16H | FACT complex subunit SPT16 OS=Homo sapiens OX=9606 GN=SUPT16H PE=1 SV=1 | 1.687603306 | 391.53 |
| DDX17 | Probable ATP-dependent RNA helicase DDX17 OS=Homo sapiens OX=9606 GN=DDX17 PE=1 SV=1 | 2.431404959 | 390.32 |
| NPM1 | Nucleophosmin (Nucleolar phosphoprotein B23, numatrin), isoform CRA_f (Fragment) OS=Homo sapiens OX=9606 GN=NPM1 PE=2 SV=1 | 1.315702479 | 388.36 |
| PABPC1 | Polyadenylate-binding protein 1 OS=Homo sapiens OX=9606 GN=PABPC1 PE=1 SV=2 | 4.325619835 | 380.56 |
| RBMX | RNA-binding motif protein, X chromosome OS=Homo sapiens OX=9606 GN=RBMX PE=1 SV=3 | 1.168595041 | 377.28 |
| HNRNPA3 | Heterogeneous nuclear ribonucleoprotein A3 OS=Homo sapiens OX=9606 GN=HNRNPA3 PE=1 SV=2 | 1.046280992 | 371.4 |
| TUBB2B | Tubulin beta-2B chain OS=Homo sapiens OX=9606 GN=TUBB2B PE=1 SV=1 | 0.532231405 | 360.82 |
| SAFB2 | Scaffold attachment factor B2 OS=Homo sapiens OX=9606 GN=SAFB2 PE=1 SV=1 | 2.153719008 | 358.67 |
| PSIP1 | PC4 and SFRS1-interacting protein OS=Homo sapiens OX=9606 GN=PSIP1 PE=1 SV=1 | 2.388429752 | 354.13 |
| DDX21 | Nucleolar RNA helicase 2 OS=Homo sapiens OX=9606 GN=DDX21 PE=1 SV=5 | 1.19338843 | 344.61 |
| TUBA1C | Tubulin alpha chain OS=Homo sapiens OX=9606 GN=TUBA1C PE=1 SV=1 | 0.459504132 | 342.81 |
| HNRNPM | Heterogeneous nuclear ribonucleoprotein M OS=Homo sapiens OX=9606 GN=HNRNPM PE=1 SV=3 | 1.933884298 | 336.08 |
| XRCC6 | X-ray repair cross-complementing protein 6 OS=Homo sapiens OX=9606 GN=XRCC6 PE=1 SV=2 | 3.041322314 | 319.79 |
| RPS4X | RPS4X protein (Fragment) OS=Homo sapiens OX=9606 GN=RPS4X PE=2 SV=2 | 1.033057851 | 311.86 |
| RBM14 | RNA binding motif protein 14 isoform 1 (Fragment) OS=Homo sapiens OX=9606 GN=RBM14 PE=2 SV=1 | 3.381818182 | 308.68 |
| HEL-S-30 | Pyruvate kinase OS=Homo sapiens OX=9606 GN=HEL-S-30 PE=1 SV=1 | 4.029752066 | 302.92 |
| HRNR | Hornerin OS=Homo sapiens OX=9606 GN=HRNR PE=1 SV=2 | 0.679338843 | 301.06 |
| ILF3 | Interleukin enhancer-binding factor 3 OS=Homo sapiens OX=9606 GN=ILF3 PE=1 SV=3 | 1.654545455 | 297.29 |
| DCD | Dermcidin OS=Homo sapiens OX=9606 GN=DCD PE=1 SV=2 | 0.829752066 | 280.7 |
| THRAP3 | Thyroid hormone receptor-associated protein 3 OS=Homo sapiens OX=9606 GN=THRAP3 PE=1 SV=2 | 1.479338843 | 280.66 |
| SSRP1 | FACT complex subunit SSRP1 OS=Homo sapiens OX=9606 GN=SSRP1 PE=1 SV=1 | 2.938842975 | 276.47 |
| HNRNPR | Heterogeneous nuclear ribonucleoprotein R OS=Homo sapiens OX=9606 GN=HNRNPR PE=1 SV=1 | 3.153719008 | 274.72 |
| PHIP | PH-interacting protein OS=Homo sapiens OX=9606 GN=PHIP PE=1 SV=2 | 6.246280992 | 271.39 |
| IGF2BP1 | Insulin-like growth factor 2 mRNA-binding protein 1 OS=Homo sapiens OX=9606 GN=IGF2BP1 PE=1 SV=2 | 2.161983471 | 269.51 |
| HNRNPA0 | Heterogeneous nuclear ribonucleoprotein A0 OS=Homo sapiens OX=9606 GN=HNRNPA0 PE=1 SV=1 | 2.474380165 | 268.34 |
| HNRNPL | Heterogeneous nuclear ribonucleoprotein L (Fragment) OS=Homo sapiens OX=9606 GN=HNRNPL PE=1 SV=1 | 3.155371901 | 266.18 |
| SLC25A5 | ADP/ATP translocase 2 OS=Homo sapiens OX=9606 GN=SLC25A5 PE=1 SV=7 | 0.717355372 | 251.73 |
| SFRS3 | Epididymis secretory sperm binding protein OS=Homo sapiens OX=9606 GN=SFRS3 PE=2 SV=1 | 1.080991736 | 244.26 |
| RAN | GTP-binding nuclear protein Ran OS=Homo sapiens OX=9606 GN=RAN PE=1 SV=1 | 1.909090909 | 237.72 |
| SYNCRIP | Heterogeneous nuclear ribonucleoprotein Q OS=Homo sapiens OX=9606 GN=SYNCRIP PE=1 SV=2 | 3.044628099 | 235.06 |
| RPL26 | 60S ribosomal protein L26 OS=Homo sapiens OX=9606 GN=RPL26 PE=1 SV=1 | 0.841322314 | 234.99 |
| HEL-S-89n | Epididymis secretory sperm binding protein Li 89n OS=Homo sapiens OX=9606 GN=HEL-S-89n PE=2 SV=1 | 1.707438017 | 234.9 |
| FBL | FBL protein (Fragment) OS=Homo sapiens OX=9606 GN=FBL PE=2 SV=2 | 2.042975207 | 231.9 |
| RPS9 | 40S ribosomal protein S9 OS=Homo sapiens OX=9606 GN=RPS9 PE=1 SV=1 | 0.644628099 | 230.98 |
| HSPD1 | Mitochondrial heat shock 60kD protein 1 variant 1 OS=Homo sapiens OX=9606 GN=HSPD1 PE=2 SV=1 | 1.634710744 | 230.68 |
| RPS8 | 40S ribosomal protein S8 OS=Homo sapiens OX=9606 GN=RPS8 PE=2 SV=1 | 1.314049587 | 230.66 |
| RPL7A | 60S ribosomal protein L7a OS=Homo sapiens OX=9606 GN=RPL7A PE=1 SV=2 | 0.694214876 | 226.85 |
| RPS3A | 40S ribosomal protein S3a OS=Homo sapiens OX=9606 GN=RPS3A PE=1 SV=2 | 0.87768595 | 219.2 |
| NUMA1 | Nuclear mitotic apparatus protein 1, isoform CRA_a OS=Homo sapiens OX=9606 GN=NUMA1 PE=4 SV=1 | 3.889256198 | 218.35 |
| CENPV | Centromere protein V OS=Homo sapiens OX=9606 GN=CENPV PE=1 SV=1 | 2.872727273 | 215.79 |
| ACIN1 | Apoptotic chromatin condensation inducer in the nucleus OS=Homo sapiens OX=9606 GN=ACIN1 PE=1 SV=1 | 1.226446281 | 215.62 |
| GTPBP4 | Nucleolar GTP-binding protein 1 OS=Homo sapiens OX=9606 GN=GTPBP4 PE=1 SV=3 | 1.899173554 | 215.58 |
| SRSF9 | Serine/arginine-rich splicing factor 9 OS=Homo sapiens OX=9606 GN=SRSF9 PE=1 SV=1 | 1.955371901 | 215.45 |
| NCL | Nucleolin, isoform CRA_b OS=Homo sapiens OX=9606 GN=NCL PE=4 SV=1 | 1.629752066 | 215.14 |
| RPN1 | Dolichyl-diphosphooligosaccharide--protein glycosyltransferase subunit 1 OS=Homo sapiens OX=9606 GN=RPN1 PE=1 SV=1 | 1.408264463 | 207.24 |
| PRDX1 | Peroxiredoxin-1 OS=Homo sapiens OX=9606 GN=PRDX1 PE=1 SV=1 | 0.912396694 | 201.34 |
| SRSF7 | Serine/arginine-rich splicing factor 7 OS=Homo sapiens OX=9606 GN=SRSF7 PE=1 SV=1 | 1.14214876 | 200.98 |
| ATP5F1A | ATP synthase subunit alpha, mitochondrial OS=Homo sapiens OX=9606 GN=ATP5F1A PE=1 SV=1 | 0.219834711 | 199.13 |
| RPL18 | 60S ribosomal protein L18 (Fragment) OS=Homo sapiens OX=9606 GN=RPL18 PE=1 SV=1 | 0.596694215 | 198.59 |
| HNRNPH1 | Heterogeneous nuclear ribonucleoprotein H OS=Homo sapiens OX=9606 GN=HNRNPH1 PE=1 SV=4 | 1.018181818 | 197.54 |
| RPS13 | 40S ribosomal protein S13 OS=Homo sapiens OX=9606 GN=RPS13 PE=1 SV=2 | 2.208264463 | 194.42 |
| RPL7 | 60S ribosomal protein L7 OS=Homo sapiens OX=9606 GN=RPL7 PE=1 SV=1 | 0.697520661 | 193.47 |
| SLC25A6 | ADP/ATP translocase 3 OS=Homo sapiens OX=9606 GN=SLC25A6 PE=1 SV=4 | 0.649586777 | 192.98 |
| RPL6 | 60S ribosomal protein L6 OS=Homo sapiens OX=9606 GN=RPL6 PE=3 SV=1 | 1.976859504 | 190.3 |
| RCC1 | CHC1 protein OS=Homo sapiens OX=9606 GN=RCC1 PE=2 SV=1 | 0.783471074 | 188.88 |
| BCLAF1 | Bcl-2-associated transcription factor 1 OS=Homo sapiens OX=9606 GN=BCLAF1 PE=1 SV=2 | 1.416528926 | 184.01 |
| NOP56 | Nucleolar protein 56 OS=Homo sapiens OX=9606 GN=NOP56 PE=1 SV=4 | 2.00661157 | 181.85 |
| PTBP1 | Polypyrimidine tract binding protein 1, isoform CRA_b OS=Homo sapiens OX=9606 GN=PTBP1 PE=1 SV=4 | 2.246280992 | 178.36 |
| RPS2 | 40S ribosomal protein S2 OS=Homo sapiens OX=9606 GN=RPS2 PE=1 SV=2 | 0.85785124 | 176.82 |
| PABPC4 | Polyadenylate-binding protein OS=Homo sapiens OX=9606 GN=PABPC4 PE=2 SV=1 | 3.748760331 | 176.54 |
| RSL1D1 | Ribosomal L1 domain-containing protein 1 OS=Homo sapiens OX=9606 GN=RSL1D1 PE=1 SV=3 | 1.760330579 | 176.02 |
| ZNF280C | Zinc finger protein 280C OS=Homo sapiens OX=9606 GN=ZNF280C PE=1 SV=1 | 3.024793388 | 172.82 |
| CBX3 | Chromobox homolog 3 (HP1 gamma homolog, Drosophila) OS=Homo sapiens OX=9606 GN=CBX3 PE=4 SV=1 | 1.140495868 | 170.34 |
| SRSF12 | Serine/arginine-rich splicing factor 12 OS=Homo sapiens OX=9606 GN=SRSF12 PE=2 SV=1 | 2.900826446 | 168 |
| HEL-S-310 | Epididymis secretory protein Li 310 OS=Homo sapiens OX=9606 GN=HEL-S-310 PE=2 SV=1 | 0.732231405 | 167.72 |
| NOP58 | Nucleolar protein 58 OS=Homo sapiens OX=9606 GN=NOP58 PE=1 SV=1 | 2.026446281 | 167.18 |
| EEF1A2 | Elongation factor 1-alpha 2 OS=Homo sapiens OX=9606 GN=EEF1A2 PE=1 SV=1 | 0.687603306 | 160.83 |
| CCDC86 | Coiled-coil domain-containing protein 86 OS=Homo sapiens OX=9606 GN=CCDC86 PE=1 SV=1 | 2.302479339 | 160.07 |
| RPL23A | 60S ribosomal protein L23a (Fragment) OS=Homo sapiens OX=9606 GN=RPL23A PE=1 SV=1 | 0.568595041 | 156.94 |
| RPS14 | 40S ribosomal protein S14 (Fragment) OS=Homo sapiens OX=9606 GN=RPS14 PE=1 SV=1 | 1.761983471 | 151.68 |
| DDX18 | RNA helicase OS=Homo sapiens OX=9606 GN=DDX18 PE=3 SV=1 | 1.616528926 | 150.93 |
| CBX5 | Chromobox protein homolog 5 OS=Homo sapiens OX=9606 GN=CBX5 PE=1 SV=1 | 1.930578512 | 149.02 |
| BAZ1B | Tyrosine-protein kinase BAZ1B OS=Homo sapiens OX=9606 GN=BAZ1B PE=1 SV=2 | 2.452892562 | 147.38 |
| KIF22 | Kinesin-like protein KIF22 OS=Homo sapiens OX=9606 GN=KIF22 PE=1 SV=5 | 2.953719008 | 146.64 |
| RPLP0 | 60S acidic ribosomal protein P0 OS=Homo sapiens OX=9606 GN=RPLP0 PE=1 SV=1 | 1.241322314 | 145.3 |
| RPL8 | 60S ribosomal protein L8 OS=Homo sapiens OX=9606 GN=RPL8 PE=1 SV=2 | 0.714049587 | 145.26 |
| RPS16 | 40S ribosomal protein S16 OS=Homo sapiens OX=9606 GN=RPS16 PE=1 SV=2 | 1.674380165 | 144.89 |
| RPL14 | RPL14 protein OS=Homo sapiens OX=9606 GN=RPL14 PE=1 SV=1 | 1.58677686 | 142.68 |
| HSP90AB1 | Heat shock protein 90kDa alpha (Cytosolic), class B member 1, isoform CRA_a OS=Homo sapiens OX=9606 GN=HSP90AB1 PE=3 SV=1 | 1.796694215 | 142.54 |
| RPL26L1 | 60S ribosomal protein L26-like 1 (Fragment) OS=Homo sapiens OX=9606 GN=RPL26L1 PE=1 SV=1 | 2.150413223 | 140.81 |
| RPL10 | 60S ribosomal protein L10 OS=Homo sapiens OX=9606 GN=RPL10 PE=1 SV=2 | 0.882644628 | 140.12 |
| KHDRBS1 | KH domain-containing, RNA-binding, signal transduction-associated protein 1 OS=Homo sapiens OX=9606 GN=KHDRBS1 PE=1 SV=1 | 2.783471074 | 139.39 |
| MATR3 | Matrin-3 OS=Homo sapiens OX=9606 GN=MATR3 PE=1 SV=1 | 1.576859504 | 139.08 |
| NOP2 | Probable 28S rRNA (cytosine(4447)-C(5))-methyltransferase OS=Homo sapiens OX=9606 GN=NOP2 PE=1 SV=2 | 1.444628099 | 137.08 |
| RPS11 | 40S ribosomal protein S11 OS=Homo sapiens OX=9606 GN=RPS11 PE=1 SV=3 | 0.609917355 | 134.46 |
| SLC25A11 | Mitochondrial 2-oxoglutarate/malate carrier protein OS=Homo sapiens OX=9606 GN=SLC25A11 PE=1 SV=3 | 1.029752066 | 133.95 |
| RCC2 | Epididymis secretory sperm binding protein OS=Homo sapiens OX=9606 GN=RCC2 PE=2 SV=1 | 2.219834711 | 131.2 |
| RPS19 | 40S ribosomal protein S19 OS=Homo sapiens OX=9606 GN=RPS19 PE=1 SV=2 | 1.823140496 | 130.46 |
| RPLP2 | 60S acidic ribosomal protein P2 OS=Homo sapiens OX=9606 GN=RPLP2 PE=1 SV=1 | 1.930578512 | 130.29 |
| RPL4 | 60S ribosomal protein L4 OS=Homo sapiens OX=9606 GN=RPL4 PE=1 SV=5 | 0.662809917 | 130.28 |
| TCP1 | T-complex protein 1 subunit alpha OS=Homo sapiens OX=9606 GN=TCP1 PE=1 SV=1 | 2.195041322 | 130.15 |
| SMARCA5 | SWI/SNF-related matrix-associated actin-dependent regulator of chromatin subfamily A member 5 OS=Homo sapiens OX=9606 GN=SMARCA5 PE=1 SV=1 | 1.709090909 | 130.11 |
| ILF2 | Interleukin enhancer-binding factor 2 OS=Homo sapiens OX=9606 GN=ILF2 PE=1 SV=1 | 0.897520661 | 130.01 |
| DSP | Desmoplakin OS=Homo sapiens OX=9606 GN=DSP PE=1 SV=3 | 0.694214876 | 129.92 |
| TMPO | Thymopoietin, isoform CRA_c OS=Homo sapiens OX=9606 GN=TMPO PE=4 SV=1 | 1.487603306 | 125.89 |
| IGKV2D-24 | Immunoglobulin kappa variable 2D-24 (non-functional) (Fragment) OS=Homo sapiens OX=9606 GN=IGKV2D-24 PE=1 SV=1 | 1.247933884 | 124.85 |
| RPL13 | 60S ribosomal protein L13 OS=Homo sapiens OX=9606 GN=RPL13 PE=1 SV=4 | 1.062809917 | 124.6 |
| DKC1 | H/ACA ribonucleoprotein complex subunit DKC1 OS=Homo sapiens OX=9606 GN=DKC1 PE=1 SV=3 | 2.084297521 | 124.31 |
| PGAM5 | Serine/threonine-protein phosphatase PGAM5, mitochondrial OS=Homo sapiens OX=9606 GN=PGAM5 PE=1 SV=2 | 1.439669421 | 124.21 |
| ATAD2 | ATPase family, AAA domain containing 2, isoform CRA_c OS=Homo sapiens OX=9606 GN=ATAD2 PE=4 SV=1 | 3.161983471 | 124.03 |
| RPL10A | 60S ribosomal protein L10a OS=Homo sapiens OX=9606 GN=RPL10A PE=1 SV=2 | 0.948760331 | 122.89 |
| RPL9; RPL9P7; RPL9P8; RPL9P9 | 60S ribosomal protein L9 OS=Homo sapiens OX=9606 GN=RPL9 PE=1 SV=1 | 0.955371901 | 122.65 |
| HEL-S-39 | Peptidyl-prolyl cis-trans isomerase OS=Homo sapiens OX=9606 GN=HEL-S-39 PE=2 SV=1 | 1.211570248 | 122.32 |
| HMGA1 | High mobility group AT-hook 1, isoform CRA_b OS=Homo sapiens OX=9606 GN=HMGA1 PE=4 SV=1 | 3.719008264 | 121.79 |
| RPS6 | 40S ribosomal protein S6 OS=Homo sapiens OX=9606 GN=RPS6 PE=2 SV=1 | 0.851239669 | 121.27 |
| RPS18 | 40S ribosomal protein S18 OS=Homo sapiens OX=9606 GN=RPS18 PE=1 SV=1 | 1.537190083 | 117.1 |
| HNRNPH2 | Heterogeneous nuclear ribonucleoprotein H2 OS=Homo sapiens OX=9606 GN=HNRNPH2 PE=1 SV=1 | 0.651239669 | 116.6 |
| UBTF | Nucleolar transcription factor 1 OS=Homo sapiens OX=9606 GN=UBTF PE=1 SV=1 | 1.975206612 | 115.72 |
| FUS | Fusion (Involved in t(1216) in malignant liposarcoma) OS=Homo sapiens OX=9606 GN=FUS PE=2 SV=1 | 3.114049587 | 115.52 |
| D10S102 | FBRNP OS=Homo sapiens OX=9606 GN=D10S102 PE=2 SV=1 | 0.016528926 | 115.44 |
| ZNF326 | DBIRD complex subunit ZNF326 OS=Homo sapiens OX=9606 GN=ZNF326 PE=1 SV=2 | 3.907438017 | 113.67 |
| L27a | Ribosomal protein L27a OS=Homo sapiens OX=9606 GN=L27a PE=4 SV=1 | 1.976859504 | 113.34 |
| CENPV | Centromere protein V OS=Homo sapiens OX=9606 GN=CENPV PE=1 SV=1 | 3.520661157 | 113.2 |
| XRCC5 | X-ray repair cross-complementing protein 5 OS=Homo sapiens OX=9606 GN=XRCC5 PE=1 SV=3 | 2.40661157 | 113.15 |
| RPS7 | 40S ribosomal protein S7 OS=Homo sapiens OX=9606 GN=RPS7 PE=1 SV=1 | 0.636363636 | 111.05 |
| RPL15 | Ribosomal protein L15 OS=Homo sapiens OX=9606 GN=RPL15 PE=3 SV=1 | 0.575206612 | 109.35 |
| RPS26 | 40S ribosomal protein S26 OS=Homo sapiens OX=9606 GN=RPS26 PE=3 SV=1 | 1.905785124 | 109.04 |
| DSC1 | Desmocollin 1, isoform CRA_b OS=Homo sapiens OX=9606 GN=DSC1 PE=4 SV=1 | 0.61322314 | 107.62 |
| UQCRC2 | Cytochrome b-c1 complex subunit 2, mitochondrial OS=Homo sapiens OX=9606 GN=UQCRC2 PE=1 SV=1 | 0.414876033 | 105.9 |
| RBM12B | RNA-binding protein 12B OS=Homo sapiens OX=9606 GN=RBM12B PE=1 SV=2 | 2.965289256 | 103.97 |
| DSG1 | Desmoglein-1 OS=Homo sapiens OX=9606 GN=DSG1 PE=1 SV=2 | 0.60661157 | 103.91 |
| SERBP1 | Plasminogen activator inhibitor 1 RNA-binding protein OS=Homo sapiens OX=9606 GN=SERBP1 PE=1 SV=2 | 0.479338843 | 103.45 |
| ELAVL1 | ELAV-like protein 1 OS=Homo sapiens OX=9606 GN=ELAVL1 PE=1 SV=2 | 1.961983471 | 102.97 |
| KAT7 | Histone acetyltransferase KAT7 OS=Homo sapiens OX=9606 GN=KAT7 PE=1 SV=1 | 3.548760331 | 101.14 |
| RPL27 | RPL27/NME2 fusion protein (Fragment) OS=Homo sapiens OX=9606 GN=RPL27 PE=2 SV=1 | 1.621487603 | 99.94 |
| RPL3 | 60S ribosomal protein L3 OS=Homo sapiens OX=9606 GN=RPL3 PE=1 SV=2 | 0.317355372 | 99.3 |
| HNRNPH3 | Heterogeneous nuclear ribonucleoprotein H3 OS=Homo sapiens OX=9606 GN=HNRNPH3 PE=1 SV=2 | 2.862809917 | 97.7 |
| ATP5B | ATP synthase subunit beta (Fragment) OS=Homo sapiens OX=9606 GN=ATP5B PE=2 SV=1 | 0.461157025 | 95.34 |
| TRA2A | Transformer-2 protein homolog alpha OS=Homo sapiens OX=9606 GN=TRA2A PE=1 SV=1 | 2.327272727 | 94.96 |
| HNRNPAB | Heterogeneous nuclear ribonucleoprotein A/B OS=Homo sapiens OX=9606 GN=HNRNPAB PE=1 SV=1 | 2.783471074 | 94.83 |
| CTDSPL2 | CTD (Carboxy-terminal domain, RNA polymerase II, polypeptide A) small phosphatase like 2, isoform CRA_a OS=Homo sapiens OX=9606 GN=CTDSPL2 PE=4 SV=1 | 3.758677686 | 94.69 |
| PRH1 | Salivary acidic proline-rich phosphoprotein 1/2 OS=Homo sapiens OX=9606 GN=PRH1 PE=4 SV=1 | 45.11239669 | 94.36 |
| HEL-S-22 | Epididymis secretory protein Li 22 OS=Homo sapiens OX=9606 GN=HEL-S-22 PE=2 SV=1 | 0.52892562 | 92.83 |
| TUFM | Elongation factor Tu OS=Homo sapiens OX=9606 GN=TUFM PE=2 SV=1 | 0.603305785 | 91.53 |
| GAPDH | Glyceraldehyde-3-phosphate dehydrogenase OS=Homo sapiens OX=9606 GN=GAPDH PE=1 SV=3 | 1.355371901 | 90.86 |
| YBX1 | Nuclease-sensitive element-binding protein 1 OS=Homo sapiens OX=9606 GN=YBX1 PE=1 SV=3 | 0.856198347 | 90.61 |
| EIF4A3 | Eukaryotic initiation factor 4A-III OS=Homo sapiens OX=9606 GN=EIF4A3 PE=1 SV=4 | 0.590082645 | 87.98 |
| TAF15 | TATA-binding protein-associated factor 2N OS=Homo sapiens OX=9606 GN=TAF15 PE=1 SV=1 | 3.700826446 | 87.87 |
| THRAP3 | THRAP3 protein (Fragment) OS=Homo sapiens OX=9606 GN=THRAP3 PE=2 SV=1 | 40.64132231 | 87.72 |
| RACK1 | Receptor of activated protein C kinase 1 OS=Homo sapiens OX=9606 GN=RACK1 PE=1 SV=3 | 1.996694215 | 87.71 |
| H1-0 | Histone H1.0 OS=Homo sapiens OX=9606 GN=H1F0 PE=1 SV=3 | 3.150413223 | 87.5 |
| ALYREF | THO complex subunit 4 OS=Homo sapiens OX=9606 GN=ALYREF PE=1 SV=1 | 0.545454545 | 87.05 |
| RPL12 | 60S ribosomal protein L12 OS=Homo sapiens OX=9606 GN=RPL12 PE=1 SV=1 | 0.85785124 | 86.84 |
| RPS25 | 40S ribosomal protein S25 OS=Homo sapiens OX=9606 GN=RPS25 PE=1 SV=1 | 2.042975207 | 86.82 |
| RPL23 | 60S ribosomal protein L23 OS=Homo sapiens OX=9606 GN=RPL23 PE=1 SV=1 | 2.140495868 | 85.46 |
| PRSS3 | Trypsin-3 OS=Homo sapiens OX=9606 GN=PRSS3 PE=1 SV=2 | 2.505785124 | 83.29 |
| PRPF19 | Pre-mRNA-processing factor 19 OS=Homo sapiens OX=9606 GN=PRPF19 PE=1 SV=1 | 1.633057851 | 81.78 |
| RPL17 | 60S ribosomal protein L17 (Fragment) OS=Homo sapiens OX=9606 GN=RPL17 PE=1 SV=1 | 0.52892562 | 81.16 |
| RPL11 | 60S ribosomal protein L11 OS=Homo sapiens OX=9606 GN=RPL11 PE=1 SV=2 | 0.81322314 | 80.88 |
| GTF2I | General transcription factor IIi isoform B (Fragment) OS=Homo sapiens OX=9606 GN=GTF2I PE=2 SV=1 | 1.07107438 | 80.6 |
| TRAP1 | TRAP1 protein (Fragment) OS=Homo sapiens OX=9606 GN=TRAP1 PE=2 SV=2 | 1.700826446 | 78.77 |
| EMD | Emerin OS=Homo sapiens OX=9606 GN=EMD PE=1 SV=1 | 1.469421488 | 78.38 |
| PPP1CC | Serine/threonine-protein phosphatase PP1-gamma catalytic subunit OS=Homo sapiens OX=9606 GN=PPP1CC PE=1 SV=1 | 1.476033058 | 78.24 |
| RPL22 | 60S ribosomal protein L22 (Fragment) OS=Homo sapiens OX=9606 GN=RPL22 PE=1 SV=1 | 1.985123967 | 78.03 |
| EL52 | Epididymis luminal secretory protein 52 OS=Homo sapiens OX=9606 GN=EL52 PE=2 SV=1 | 2.00661157 | 77.46 |
| IGF2BP3 | Insulin-like growth factor 2 mRNA-binding protein 3 OS=Homo sapiens OX=9606 GN=IGF2BP3 PE=1 SV=2 | 1.92231405 | 76.5 |
| HCTP4 | HCTP4 OS=Homo sapiens OX=9606 GN=HCTP4 PE=1 SV=1 | 2.760330579 | 76.2 |
| RPL31 | 60S ribosomal protein L31 (Fragment) OS=Homo sapiens OX=9606 GN=RPL31 PE=1 SV=1 | 1.803305785 | 76.1 |
| FARSLA | FARSLA protein OS=Homo sapiens OX=9606 GN=FARSLA PE=2 SV=1 | 1.937190083 | 75.93 |
| CBS | Cystathionine beta-synthase-like protein OS=Homo sapiens OX=9606 GN=CBSL PE=1 SV=1 | 1.859504132 | 75.34 |
| KPLCE | Skin-specific protein 32 OS=Homo sapiens OX=9606 GN=XP32 PE=1 SV=1 | 0.687603306 | 75.18 |
| OK/KNS-cl.6 | Ribosomal protein S2 OS=Homo sapiens OX=9606 GN=OK/KNS-cl.6 PE=2 SV=1 | 1.971900826 | 74.84 |
| SRSF5 | Serine/arginine-rich splicing factor 5 OS=Homo sapiens OX=9606 GN=SRSF5 PE=1 SV=1 | 1.472727273 | 73.41 |
| CDCA5 | Sororin OS=Homo sapiens OX=9606 GN=CDCA5 PE=1 SV=1 | 2.687603306 | 72.92 |
| HBB | Beta-globin OS=Homo sapiens OX=9606 GN=HBB PE=3 SV=1 | 8.975206612 | 72.85 |
| U2AF2 | Splicing factor U2AF 65 kDa subunit OS=Homo sapiens OX=9606 GN=U2AF2 PE=1 SV=4 | 2.143801653 | 72.07 |
| TMPO | Lamina-associated polypeptide 2, isoform alpha OS=Homo sapiens OX=9606 GN=TMPO PE=1 SV=2 | 2.872727273 | 71.69 |
| CTPS1 | CTP synthase 1 OS=Homo sapiens OX=9606 GN=CTPS1 PE=1 SV=2 | 1.621487603 | 71.09 |
| SRRM1 | Serine/arginine repetitive matrix 1 isoform 2 (Fragment) OS=Homo sapiens OX=9606 GN=SRRM1 PE=1 SV=1 | 1.818181818 | 70.77 |
| SSBP1 | Single-stranded DNA binding protein 1 OS=Homo sapiens OX=9606 GN=SSBP1 PE=2 SV=1 | 6.682644628 | 69.3 |
| H1-10 | Histone H1x OS=Homo sapiens OX=9606 GN=H1FX PE=1 SV=1 | 1.480991736 | 68.97 |
| IGF2BP2 | Insulin-like growth factor 2 mRNA-binding protein 2 OS=Homo sapiens OX=9606 GN=IGF2BP2 PE=1 SV=2 | 2.433057851 | 68.72 |
| VIM | Vimentin OS=Homo sapiens OX=9606 GN=VIM PE=1 SV=4 | 0.495867769 | 68.36 |
| RPL19 | 60S ribosomal protein L19 OS=Homo sapiens OX=9606 GN=RPL19 PE=1 SV=1 | 0.914049587 | 68.12 |
| SFRS4 | Splicing factor, arginine/serine-rich 4, isoform CRA_b OS=Homo sapiens OX=9606 GN=SFRS4 PE=2 SV=1 | 2.229752066 | 66.62 |
| VDAC2 | Voltage-dependent anion-selective channel protein 2 (Fragment) OS=Homo sapiens OX=9606 GN=VDAC2 PE=1 SV=1 | 1.596694215 | 65.68 |
| GNL3 | Guanine nucleotide-binding protein-like 3 OS=Homo sapiens OX=9606 GN=GNL3 PE=1 SV=2 | 1.581818182 | 65.58 |
| ZNF512 | Zinc finger protein 512 OS=Homo sapiens OX=9606 GN=ZNF512 PE=1 SV=2 | 3.076033058 | 64.37 |
| HBA2 | Alpha-2 globin chain OS=Homo sapiens OX=9606 GN=HBA2 PE=3 SV=1 | 7.695867769 | 64.25 |
| PES1 | Pescadillo homolog OS=Homo sapiens OX=9606 GN=PES1 PE=1 SV=1 | 1.514049587 | 64.16 |
| MKI67 | KI67 Antigen (Fragment) OS=Homo sapiens OX=9606 GN=MKI67 PE=2 SV=1 | 7.074380165 | 61.76 |
| HNRNPF | Heterogeneous nuclear ribonucleoprotein F OS=Homo sapiens OX=9606 GN=HNRNPF PE=1 SV=3 | 0.842975207 | 61.13 |
| LOC101345898; LOC101577117; LOC103788504; LOC111157481; LOC112631343; LOC112825549; LOC116746263; LOC478509; LOC702677; Rpl34; RPL34 | Ribosomal protein L34, isoform CRA_a OS=Homo sapiens OX=9606 GN=RPL34 PE=4 SV=1 | 1.907438017 | 60.66 |
| PPP1CA | Serine/threonine-protein phosphatase PP1-alpha catalytic subunit OS=Homo sapiens OX=9606 GN=PPP1CA PE=1 SV=1 | 2.181818182 | 59.01 |
| ATAD3B | ATPase family AAA domain-containing protein 3B OS=Homo sapiens OX=9606 GN=ATAD3B PE=1 SV=1 | 2.398347107 | 58.67 |
| RPS23 | Ribosomal protein S23, isoform CRA_a OS=Homo sapiens OX=9606 GN=RPS23 PE=2 SV=1 | 2.760330579 | 58.09 |
| LDHA | L-lactate dehydrogenase A chain OS=Homo sapiens OX=9606 GN=LDHA PE=1 SV=2 | 1.667768595 | 57.82 |
| DNAJC9 | DnaJ homolog subfamily C member 9 OS=Homo sapiens OX=9606 GN=DNAJC9 PE=1 SV=1 | 1.571900826 | 57.69 |
| EIF2S1 | Eukaryotic translation initiation factor 2 subunit 1 OS=Homo sapiens OX=9606 GN=EIF2S1 PE=1 SV=3 | 1.436363636 | 57.2 |
| EEF2 | Elongation factor 2 OS=Homo sapiens OX=9606 GN=EEF2 PE=1 SV=4 | 1.540495868 | 56.94 |
| CBX1 | Chromobox protein homolog 1 (Fragment) OS=Homo sapiens OX=9606 GN=CBX1 PE=1 SV=8 | 2.424793388 | 56.81 |
| UTP15 | U3 small nucleolar RNA-associated protein 15 homolog OS=Homo sapiens OX=9606 GN=UTP15 PE=1 SV=3 | 2.185123967 | 55.58 |
| PPP1CB | Serine/threonine-protein phosphatase PP1-beta catalytic subunit OS=Homo sapiens OX=9606 GN=PPP1CB PE=1 SV=3 | 2.041322314 | 54.92 |
| RPL35 | 60S ribosomal protein L35 OS=Homo sapiens OX=9606 GN=RPL35 PE=1 SV=2 | 1.530578512 | 54.32 |
| DDX1 | ATP-dependent RNA helicase DDX1 OS=Homo sapiens OX=9606 GN=DDX1 PE=2 SV=1 | 1.937190083 | 54.09 |
| ARF3 | ADP-ribosylation factor 3 OS=Homo sapiens OX=9606 GN=ARF3 PE=1 SV=2 | 3.352066116 | 54.06 |
| EEF1G | Elongation factor 1-gamma OS=Homo sapiens OX=9606 GN=EEF1G PE=1 SV=3 | 0.395041322 | 53.64 |
| RPL30 | 60S ribosomal protein L30 (Fragment) OS=Homo sapiens OX=9606 GN=RPL30 PE=1 SV=1 | 3.667768595 | 53.47 |
| WDR43 | WD repeat-containing protein 43 OS=Homo sapiens OX=9606 GN=WDR43 PE=1 SV=3 | 1.905785124 | 53.38 |
| CHTOP | Chromatin target of PRMT1 protein OS=Homo sapiens OX=9606 GN=CHTOP PE=1 SV=1 | 1.558677686 | 53.27 |
| AMY1A | Alpha-amylase OS=Homo sapiens OX=9606 GN=AMY1A PE=2 SV=1 | 1.514049587 | 53.17 |
| AURKB | Aurora kinase B OS=Homo sapiens OX=9606 GN=AURKB PE=1 SV=3 | 2.229752066 | 53.17 |
| UTP18 | U3 small nucleolar RNA-associated protein 18 homolog OS=Homo sapiens OX=9606 GN=UTP18 PE=1 SV=3 | 1.985123967 | 53.1 |
| VRK1 | Serine/threonine-protein kinase VRK1 OS=Homo sapiens OX=9606 GN=VRK1 PE=1 SV=1 | 0.705785124 | 52.82 |
| STRBP | Spermatid perinuclear RNA binding protein isoform 2 (Fragment) OS=Homo sapiens OX=9606 GN=STRBP PE=1 SV=1 | 2.699173554 | 51.9 |
| YWHAE/FAM22B fusion | YWHAE/FAM22B fusion protein (Fragment) OS=Homo sapiens OX=9606 GN=YWHAE/FAM22B fusion PE=2 SV=1 | 1.381818182 | 51.9 |
| PABPN1 | Polyadenylate-binding protein 2 OS=Homo sapiens OX=9606 GN=PABPN1 PE=1 SV=3 | 1.876033058 | 51.76 |
| HNRNPD | Heterogeneous nuclear ribonucleoprotein D0 (Fragment) OS=Homo sapiens OX=9606 GN=HNRNPD PE=1 SV=8 | 1.370247934 | 51.46 |
| HNRNPUL2-BSCL2 | HCG2044799 OS=Homo sapiens OX=9606 GN=HNRNPUL2-BSCL2 PE=4 SV=1 | 2.520661157 | 51.22 |
| RPL28 | 60S ribosomal protein L28 OS=Homo sapiens OX=9606 GN=RPL28 PE=1 SV=1 | 1.629752066 | 50.31 |
| SNRNP200 | U5 small nuclear ribonucleoprotein 200 kDa helicase OS=Homo sapiens OX=9606 GN=SNRNP200 PE=1 SV=2 | 3.171900826 | 48.75 |
| RPL36 | 60S ribosomal protein L36 OS=Homo sapiens OX=9606 GN=RPL36 PE=1 SV=3 | 3.517355372 | 46.95 |
| RFC4 | Replication factor C subunit 4 OS=Homo sapiens OX=9606 GN=RFC4 PE=1 SV=2 | 2.897520661 | 46.65 |
| KDM2A | F-box and leucine-rich repeat protein 11, isoform CRA_a OS=Homo sapiens OX=9606 GN=KDM2A PE=2 SV=1 | 2.439669421 | 46.52 |
| YWHAZ | Epididymis luminal protein 4 OS=Homo sapiens OX=9606 GN=YWHAZ PE=2 SV=1 | 1.485950413 | 46.52 |
| PDCD11 | Protein RRP5 homolog OS=Homo sapiens OX=9606 GN=PDCD11 PE=1 SV=3 | 3.026446281 | 45.94 |
| ARF4 | ADP-ribosylation factor 4 OS=Homo sapiens OX=9606 GN=ARF4 PE=1 SV=3 | 2.466115702 | 45.19 |
| KPNA2 | Importin subunit alpha-1 OS=Homo sapiens OX=9606 GN=KPNA2 PE=1 SV=1 | 1.747107438 | 45.1 |
| PCMT1 | Protein-L-isoaspartate(D-aspartate) O-methyltransferase OS=Homo sapiens OX=9606 GN=PCMT1 PE=1 SV=4 | 1.428099174 | 44.01 |
| UHRF1 | E3 ubiquitin-protein ligase UHRF1 OS=Homo sapiens OX=9606 GN=UHRF1 PE=1 SV=1 | 1.995041322 | 43.84 |
| SNRPB | Small nuclear ribonucleoprotein-associated protein OS=Homo sapiens OX=9606 GN=SNRPB PE=2 SV=1 | 1.300826446 | 43.75 |
| RPL32 | 60S ribosomal protein L32 (Fragment) OS=Homo sapiens OX=9606 GN=RPL32 PE=1 SV=1 | 2.080991736 | 43.42 |
| FAM98A | Family with sequence similarity 98, member A OS=Homo sapiens OX=9606 GN=FAM98A PE=2 SV=1 | 2.851239669 | 42.93 |
| RBBP4 | Histone-binding protein RBBP4 OS=Homo sapiens OX=9606 GN=RBBP4 PE=1 SV=3 | 0.609917355 | 42.58 |
| PNN | Pinin OS=Homo sapiens OX=9606 GN=PNN PE=1 SV=5 | 1.401652893 | 42.34 |
| LMNB1 | Lamin-B1 OS=Homo sapiens OX=9606 GN=LMNB1 PE=1 SV=2 | 1.796694215 | 42.27 |
| LYZF1 | Lysozyme OS=Homo sapiens OX=9606 GN=LYZF1 PE=2 SV=1 | 0.310743802 | 41.19 |
| CCT8 | T-complex protein 1 subunit theta OS=Homo sapiens OX=9606 GN=CCT8 PE=1 SV=4 | 2.451239669 | 41.18 |
| RPL22L1 | 60S ribosomal protein L22-like 1 OS=Homo sapiens OX=9606 GN=RPL22L1 PE=1 SV=1 | 2.297520661 | 41.04 |
| PRPS1 | Ribose-phosphate pyrophosphokinase 1 OS=Homo sapiens OX=9606 GN=PRPS1 PE=1 SV=2 | 1.34214876 | 40.98 |
| HP1BP3 | Heterochromatin protein 1-binding protein 3 OS=Homo sapiens OX=9606 GN=HP1BP3 PE=1 SV=1 | 1.692561983 | 40.94 |
| XPC | Xeroderma pigmentosum, complementation group C isoform A (Fragment) OS=Homo sapiens OX=9606 GN=XPC PE=2 SV=1 | 2.914049587 | 40.63 |
| PARP2 | Poly [ADP-ribose] polymerase OS=Homo sapiens OX=9606 GN=PARP2 PE=1 SV=1 | 3.224793388 | 40.55 |
| DDX27 | Probable ATP-dependent RNA helicase DDX27 OS=Homo sapiens OX=9606 GN=DDX27 PE=1 SV=2 | 1.323966942 | 40.38 |
| DDX27 | DEAD box polypeptide 27 OS=Homo sapiens OX=9606 GN=DDX27 PE=2 SV=1 | 1.282644628 | 40.38 |
| AIFM1 | Apoptosis-inducing factor 1, mitochondrial OS=Homo sapiens OX=9606 GN=AIFM1 PE=1 SV=1 | 1.315702479 | 40.34 |
| GSTP1 | Glutathione S-transferase pi (Fragment) OS=Homo sapiens OX=9606 GN=GSTP1 PE=2 SV=1 | 0.376859504 | 40.33 |
| PPAN-P2RY11 | HCG2039996 OS=Homo sapiens OX=9606 GN=PPAN-P2RY11 PE=3 SV=1 | 2.036363636 | 40.28 |
| PRSS1 | Trypsin-1 OS=Homo sapiens OX=9606 GN=PRSS1 PE=1 SV=1 | 0.723966942 | 39.71 |
| YWHAQ | 14-3-3 protein theta OS=Homo sapiens OX=9606 GN=YWHAQ PE=1 SV=1 | 1.616528926 | 39.1 |
| RFC1 | Replication factor C subunit 1 OS=Homo sapiens OX=9606 GN=RFC1 PE=1 SV=4 | 2.692561983 | 37.86 |
| RPS15 | 40S ribosomal protein S15 OS=Homo sapiens OX=9606 GN=RPS15 PE=1 SV=2 | 2.090909091 | 37.81 |
| MYBBP1A | Myb-binding protein 1A OS=Homo sapiens OX=9606 GN=MYBBP1A PE=1 SV=2 | 1.297520661 | 37.57 |
| LOC117799893; RPSA | 40S ribosomal protein SA OS=Homo sapiens OX=9606 GN=RPSA PE=3 SV=1 | 2.702479339 | 37.33 |
| RPN2 | Dolichyl-diphosphooligosaccharide--protein glycosyltransferase subunit 2 OS=Homo sapiens OX=9606 GN=RPN2 PE=1 SV=3 | 1.601652893 | 37.08 |
| EIF2S3 | Eukaryotic translation initiation factor 2 subunit 3 OS=Homo sapiens OX=9606 GN=EIF2S3 PE=1 SV=3 | 0.280991736 | 36.71 |
| H2A/k | Histone H2A OS=Homo sapiens OX=9606 GN=H2A/k PE=3 SV=1 | 2.33553719 | 36.21 |
| CST4 | Cystatin-S OS=Homo sapiens OX=9606 GN=CST4 PE=1 SV=3 | 1.137190083 | 36.14 |
| CMAS | N-acylneuraminate cytidylyltransferase OS=Homo sapiens OX=9606 GN=CMAS PE=1 SV=2 | 0.839669421 | 35.63 |
| SMARCA1 | Probable global transcription activator SNF2L1 OS=Homo sapiens OX=9606 GN=SMARCA1 PE=1 SV=2 | 1.943801653 | 35.59 |
| FXR1 | Fragile X mental retardation autosomal homolog variant p5FK OS=Homo sapiens OX=9606 GN=FXR1 PE=2 SV=1 | 1.52892562 | 35.47 |
| MECP2 | Methyl-CpG-binding protein 2 OS=Homo sapiens OX=9606 GN=MECP2 PE=2 SV=1 | 2.408264463 | 35.3 |
| PHB2 | Prohibitin-2 OS=Homo sapiens OX=9606 GN=PHB2 PE=1 SV=1 | 2.241322314 | 35.15 |
| RPS20 | 40S ribosomal protein S20 OS=Homo sapiens OX=9606 GN=RPS20 PE=1 SV=1 | 1.74214876 | 35.04 |
| PIP | Prolactin-inducible protein OS=Homo sapiens OX=9606 GN=PIP PE=1 SV=1 | 1.842975207 | 35.01 |
| SNRP70 | Small nuclear ribonucleoprotein 70kDa polypeptide (RNP antigen), isoform CRA_b OS=Homo sapiens OX=9606 GN=SNRP70 PE=4 SV=1 | 3.123966942 | 34.86 |
| HEL103 | Signal recognition particle subunit SRP72 OS=Homo sapiens OX=9606 GN=HEL103 PE=2 SV=1 | 1.317355372 | 34.76 |
| RPL29 | 60S ribosomal protein L29 OS=Homo sapiens OX=9606 GN=RPL29 PE=1 SV=1 | 0.725619835 | 34.69 |
| YWHAB | 14-3-3 protein beta/alpha OS=Homo sapiens OX=9606 GN=YWHAB PE=1 SV=3 | 1.788429752 | 34.11 |
| DDB1 | DNA damage-binding protein 1 OS=Homo sapiens OX=9606 GN=DDB1 PE=1 SV=1 | 1.674380165 | 34.07 |
| ATP5PO | ATP synthase subunit O, mitochondrial OS=Homo sapiens OX=9606 GN=ATP5PO PE=1 SV=1 | 0.631404959 | 33.78 |
| HEL-S-125m | Epididymis luminal protein 35 OS=Homo sapiens OX=9606 GN=HEL-S-125m PE=2 SV=1 | 1.596694215 | 33.77 |
| JUP | Junction plakoglobin isoform 1 (Fragment) OS=Homo sapiens OX=9606 GN=JUP PE=2 SV=1 | 0.732231405 | 33.65 |
| YBX3 | Y-box-binding protein 3 OS=Homo sapiens OX=9606 GN=YBX3 PE=1 SV=4 | 0.525619835 | 33.13 |
| FLJ10292 | Mago nashi protein OS=Homo sapiens OX=9606 GN=FLJ10292 PE=2 SV=1 | 4.102479339 | 32.99 |
| BRD1 | Bromodomain containing 1, isoform CRA_a OS=Homo sapiens OX=9606 GN=BRD1 PE=4 SV=1 | 2.284297521 | 32.97 |
| EBNA1BP2 | Probable rRNA-processing protein EBP2 OS=Homo sapiens OX=9606 GN=EBNA1BP2 PE=1 SV=2 | 0.310743802 | 32.55 |
| RPS5 | 40S ribosomal protein S5 OS=Homo sapiens OX=9606 GN=RPS5 PE=1 SV=1 | 0.704132231 | 31.76 |
| SFPQ | SFPQ protein (Fragment) OS=Homo sapiens OX=9606 GN=SFPQ PE=2 SV=2 | 1.715702479 | 31.53 |
| PRPF8 | Pre-mRNA-processing-splicing factor 8 OS=Homo sapiens OX=9606 GN=PRPF8 PE=1 SV=2 | 3.550413223 | 31.51 |
| ACIN1 | Apoptotic chromatin condensation inducer in the nucleus (Fragment) OS=Homo sapiens OX=9606 GN=ACIN1 PE=1 SV=1 | 1.423140496 | 31.34 |
| RFC5 | RFC5 protein (Fragment) OS=Homo sapiens OX=9606 GN=RFC5 PE=2 SV=1 | 2.990082645 | 30.99 |
| DNTTIP2 | Deoxynucleotidyltransferase terminal-interacting protein 2 OS=Homo sapiens OX=9606 GN=DNTTIP2 PE=1 SV=2 | 2.485950413 | 30.66 |
| RRP1B | Ribosomal RNA processing protein 1 homolog B OS=Homo sapiens OX=9606 GN=RRP1B PE=1 SV=3 | 1.20661157 | 30.61 |
| PRDX6 | Peroxiredoxin-6 OS=Homo sapiens OX=9606 GN=PRDX6 PE=1 SV=3 | 2.221487603 | 30.22 |
| CDCA8 | Borealin OS=Homo sapiens OX=9606 GN=CDCA8 PE=1 SV=2 | 2.87768595 | 30.19 |
| CCDC124 | Coiled-coil domain-containing protein 124 OS=Homo sapiens OX=9606 GN=CCDC124 PE=1 SV=1 | 0.879338843 | 30.12 |
| CCT4 | T-complex protein 1 subunit delta OS=Homo sapiens OX=9606 GN=CCT4 PE=1 SV=4 | 1.836363636 | 30.1 |
| H1-5 | Histone H1.5 OS=Homo sapiens OX=9606 GN=HIST1H1B PE=1 SV=3 | 2.319008264 | 29.99 |
| SRP68 | Signal recognition particle subunit SRP68 OS=Homo sapiens OX=9606 GN=SRP68 PE=1 SV=2 | 1.472727273 | 29.67 |
| UTP4 | U3 small nucleolar RNA-associated protein 4 homolog OS=Homo sapiens OX=9606 GN=UTP4 PE=1 SV=1 | 1.641322314 | 29.59 |
| RFC2 | Replication factor C subunit 2 OS=Homo sapiens OX=9606 GN=RFC2 PE=1 SV=3 | 2.771900826 | 29.54 |
| PRPS2 | Ribose-phosphate pyrophosphokinase 2 OS=Homo sapiens OX=9606 GN=PRPS2 PE=1 SV=2 | 1.47768595 | 29.39 |
| LDHB | L-lactate dehydrogenase B chain OS=Homo sapiens OX=9606 GN=LDHB PE=1 SV=2 | 1.702479339 | 29.37 |
| SLTM | SAFB-like transcription modulator OS=Homo sapiens OX=9606 GN=SLTM PE=1 SV=2 | 2.466115702 | 28.63 |
| NTHL1 | Endonuclease III-like protein 1 OS=Homo sapiens OX=9606 GN=NTHL1 PE=1 SV=2 | 3.371900826 | 28.62 |
| RUVBL1 | RuvB-like helicase (Fragment) OS=Homo sapiens OX=9606 GN=RUVBL1 PE=2 SV=1 | 0.26446281 | 28.51 |
| SNW1 | SNW1 protein OS=Homo sapiens OX=9606 GN=SNW1 PE=2 SV=1 | 1.52231405 | 28.49 |
| HMGA1 | High mobility group AT-hook 1 OS=Homo sapiens OX=9606 GN=HMGA1 PE=2 SV=1 | 5.272727273 | 28.38 |
| HMGB2 | High mobility group protein B2 OS=Homo sapiens OX=9606 GN=HMGB2 PE=1 SV=2 | 1.854545455 | 27.83 |
| CHAF1B | Chromatin assembly factor 1 subunit B OS=Homo sapiens OX=9606 GN=CHAF1B PE=1 SV=1 | 2.092561983 | 27.26 |
| YWHAH | Tyrosine 3-monooxygenase/tryptophan 5-monooxygenase activation protein, eta polypeptide, isoform CRA_b OS=Homo sapiens OX=9606 GN=YWHAH PE=3 SV=1 | 2.132231405 | 27.07 |
| HSP90AB4P | Putative heat shock protein HSP 90-beta 4 OS=Homo sapiens OX=9606 GN=HSP90AB4P PE=5 SV=1 | 2.887603306 | 26.73 |
| C6orf11 | Chromosome 6 open reading frame 11 OS=Homo sapiens OX=9606 GN=WDR46 PE=2 SV=1 | 2.044628099 | 26.15 |
| CASP14 | Caspase 14, apoptosis-related cysteine peptidase OS=Homo sapiens OX=9606 GN=CASP14 PE=2 SV=1 | 0.717355372 | 25.96 |
| SNC73 | SNC73 protein OS=Homo sapiens OX=9606 GN=SNC73 PE=2 SV=1 | 1.689256198 | 25.45 |
| HACD3 | Very-long-chain (3R)-3-hydroxyacyl-CoA dehydratase 3 OS=Homo sapiens OX=9606 GN=HACD3 PE=1 SV=2 | 1.525619835 | 25.33 |
| NAT10 | RNA cytidine acetyltransferase OS=Homo sapiens OX=9606 GN=NAT10 PE=1 SV=2 | 1.158677686 | 25.3 |
| TOMM22 | Mitochondrial import receptor subunit TOM22 homolog OS=Homo sapiens OX=9606 GN=TOMM22 PE=1 SV=3 | 3.814876033 | 24.88 |
| NOL11 | Nucleolar protein 11 OS=Homo sapiens OX=9606 GN=NOL11 PE=1 SV=1 | 2.016528926 | 24.86 |
| PWP1 | PWP1 homolog (S. cerevisiae) OS=Homo sapiens OX=9606 GN=PWP1 PE=2 SV=1 | 1.287603306 | 24.63 |
| C14orf166 | CLE7 OS=Homo sapiens OX=9606 GN=C14orf166 PE=2 SV=1 | 1.474380165 | 24.59 |
| EIF5AL1 | Eukaryotic translation initiation factor 5A-1-like OS=Homo sapiens OX=9606 GN=EIF5AL1 PE=2 SV=2 | 4.090909091 | 24.52 |
| RPL5 | 60S ribosomal protein L5 OS=Homo sapiens OX=9606 GN=RPL5 PE=1 SV=3 | 1.076033058 | 24.32 |
| PHGDH | D-3-phosphoglycerate dehydrogenase OS=Homo sapiens OX=9606 GN=PHGDH PE=1 SV=4 | 0.80661157 | 24.01 |
| HMGN1 | High-mobility group nucleosome binding domain 1 OS=Homo sapiens OX=9606 GN=HMGN1 PE=2 SV=1 | 1.950413223 | 23.66 |
| DARS1 | Aspartate--tRNA ligase, cytoplasmic OS=Homo sapiens OX=9606 GN=DARS PE=1 SV=2 | 1.682644628 | 23.58 |
| RTCB | tRNA-splicing ligase RtcB homolog OS=Homo sapiens OX=9606 GN=RTCB PE=1 SV=1 | 1.537190083 | 23.27 |
| PCNA | Proliferating cell nuclear antigen (Fragment) OS=Homo sapiens OX=9606 GN=PCNA PE=2 SV=1 | 0.85785124 | 23.1 |
| COX2 | Cytochrome c oxidase subunit 2 OS=Homo sapiens OX=9606 GN=COX2 PE=3 SV=1 | 0.851239669 | 22.7 |
| FLG2 | Filaggrin-2 OS=Homo sapiens OX=9606 GN=FLG2 PE=1 SV=1 | 0.882644628 | 22.61 |
| PCBP2 | Poly(rC)-binding protein 2 OS=Homo sapiens OX=9606 GN=PCBP2 PE=1 SV=1 | 1.902479339 | 22.44 |
| INCENP | Inner centromere protein OS=Homo sapiens OX=9606 GN=INCENP PE=1 SV=3 | 1.953719008 | 22.41 |
| S100A8 | Protein S100-A8 OS=Homo sapiens OX=9606 GN=S100A8 PE=1 SV=1 | 0.890909091 | 22.41 |
| C8orf33 | UPF0488 protein C8orf33 OS=Homo sapiens OX=9606 GN=C8orf33 PE=1 SV=1 | 1.391735537 | 22.38 |
| RNPS1 | RNA-binding protein with serine-rich domain 1 (Fragment) OS=Homo sapiens OX=9606 GN=RNPS1 PE=1 SV=1 | 0.492561983 | 22.35 |
| HEL-S-15 | Cofilin 1 (Non-muscle), isoform CRA_b OS=Homo sapiens OX=9606 GN=HEL-S-15 PE=2 SV=1 | 1.566942149 | 22.27 |
| NIFK | MKI67 FHA domain-interacting nucleolar phosphoprotein OS=Homo sapiens OX=9606 GN=NIFK PE=1 SV=1 | 1.395041322 | 22.14 |
| PFKL | ATP-dependent 6-phosphofructokinase, liver type OS=Homo sapiens OX=9606 GN=PFKL PE=1 SV=6 | 1.780165289 | 22.11 |
| PCBP1 | Poly(rC)-binding protein 1 OS=Homo sapiens OX=9606 GN=PCBP1 PE=1 SV=2 | 1.880991736 | 21.93 |
| ZCCHC10 | Zinc finger CCHC domain-containing protein 10 OS=Homo sapiens OX=9606 GN=ZCCHC10 PE=1 SV=1 | 3.196694215 | 21.92 |
| ABCF1 | ABC50 protein OS=Homo sapiens OX=9606 GN=ABCF1 PE=4 SV=1 | 1.299173554 | 21.8 |
| AHCTF1 | Protein ELYS OS=Homo sapiens OX=9606 GN=AHCTF1 PE=1 SV=3 | 5.702479339 | 21.51 |
| MCM7 | DNA replication licensing factor MCM7 OS=Homo sapiens OX=9606 GN=MCM7 PE=1 SV=4 | 1.062809917 | 21.22 |
| NME2 | Nucleoside diphosphate kinase B OS=Homo sapiens OX=9606 GN=NME2 PE=1 SV=1 | 4.957024793 | 21.01 |
| U2AF1 | Splicing factor U2AF 35 kDa subunit OS=Homo sapiens OX=9606 GN=U2AF1 PE=1 SV=3 | 0.98677686 | 20.83 |
| DEK | DEK oncogene (DNA binding), isoform CRA_b OS=Homo sapiens OX=9606 GN=DEK PE=1 SV=2 | 0.694214876 | 20.79 |
| EIF2S2 | Eukaryotic translation initiation factor 2 beta OS=Homo sapiens OX=9606 GN=EIF2S2 PE=2 SV=1 | 0.619834711 | 20.7 |
| SLX9 | Protein FAM207A OS=Homo sapiens OX=9606 GN=FAM207A PE=1 SV=2 | 1.444628099 | 20.32 |
| PLRG1 | Pleiotropic regulator 1 OS=Homo sapiens OX=9606 GN=PLRG1 PE=1 SV=1 | 1.788429752 | 20.21 |
| G3BP2 | Ras-GTPase activating protein SH3 domain-binding protein 2, isoform CRA_b OS=Homo sapiens OX=9606 GN=G3BP2 PE=4 SV=1 | 2.034710744 | 19.98 |
| SNRPD3 | Small nuclear ribonucleoprotein Sm D3 OS=Homo sapiens OX=9606 GN=SNRPD3 PE=1 SV=1 | 3.337190083 | 19.82 |
| NCOA5 | Nuclear receptor coactivator 5 OS=Homo sapiens OX=9606 GN=NCOA5 PE=1 SV=2 | 2.981818182 | 19.14 |
| FLJ00385 | FLJ00385 protein (Fragment) OS=Homo sapiens OX=9606 GN=FLJ00385 PE=1 SV=1 | 1.224793388 | 19.04 |
| GAR1 | H/ACA ribonucleoprotein complex subunit 1 OS=Homo sapiens OX=9606 GN=GAR1 PE=1 SV=1 | 1.246280992 | 19 |
| EIF6 | Eukaryotic translation initiation factor 6 OS=Homo sapiens OX=9606 GN=EIF6 PE=1 SV=1 | 1.267768595 | 18.8 |
| LBR | Lamin B receptor, isoform CRA_a OS=Homo sapiens OX=9606 GN=LBR PE=4 SV=1 | 2.033057851 | 18.3 |
| UPF3B | Regulator of nonsense transcripts 3B OS=Homo sapiens OX=9606 GN=UPF3B PE=1 SV=1 | 1.700826446 | 18.13 |
| ABCF2 | ATP-binding cassette, sub-family F (GCN20), member 2 OS=Homo sapiens OX=9606 GN=ABCF2 PE=4 SV=1 | 1.482644628 | 18.11 |
| RFC3 | Replication factor C (Activator 1) 3, 38kDa, isoform CRA_a OS=Homo sapiens OX=9606 GN=RFC3 PE=4 SV=1 | 2.155371901 | 18.05 |
| BOP1 | Ribosome biogenesis protein BOP1 OS=Homo sapiens OX=9606 GN=BOP1 PE=2 SV=1 | 1.180165289 | 18 |
| FMR1 | Fragile X mental retardation 1 OS=Homo sapiens OX=9606 GN=FMR1 PE=2 SV=1 | 1.378512397 | 17.94 |
| TMA16 | Translation machinery-associated protein 16 (Fragment) OS=Homo sapiens OX=9606 GN=TMA16 PE=1 SV=1 | 1.639669421 | 17.77 |
| RPLP1 | Ribosomal protein, large, P1, isoform CRA_a OS=Homo sapiens OX=9606 GN=RPLP1 PE=3 SV=1 | 0.727272727 | 17.67 |
| RUVBL2 | RuvB-like 2 OS=Homo sapiens OX=9606 GN=RUVBL2 PE=1 SV=3 | 0.58677686 | 17.59 |
| PRDX4 | Peroxiredoxin-4 OS=Homo sapiens OX=9606 GN=PRDX4 PE=1 SV=1 | 0.996694215 | 17.58 |
| G3BP | G3BP protein OS=Homo sapiens OX=9606 GN=G3BP PE=2 SV=1 | 1.874380165 | 17.33 |
| FABP5 | Fatty acid-binding protein 5 OS=Homo sapiens OX=9606 GN=FABP5 PE=1 SV=3 | 1.067768595 | 16.91 |
| TIMM44 | Mitochondrial import inner membrane translocase subunit TIM44 OS=Homo sapiens OX=9606 GN=TIMM44 PE=1 SV=2 | 0.345454545 | 16.75 |
| ENO1 | Alpha-enolase OS=Homo sapiens OX=9606 GN=ENO1 PE=1 SV=2 | 1.152066116 | 16.5 |
| RPLP1 | RPLP1 protein OS=Homo sapiens OX=9606 GN=RPLP1 PE=2 SV=1 | 1.204958678 | 16.5 |
| RAB5C | RAB5C, member RAS oncogene family, isoform CRA_a OS=Homo sapiens OX=9606 GN=RAB5C PE=4 SV=1 | 1.616528926 | 16.41 |
| KHDRBS3 | KH domain-containing, RNA-binding, signal transduction-associated protein 3 OS=Homo sapiens OX=9606 GN=KHDRBS3 PE=1 SV=1 | 2.114049587 | 16.13 |
| STAU1 | Double-stranded RNA-binding protein Staufen homolog 1 OS=Homo sapiens OX=9606 GN=STAU1 PE=1 SV=2 | 1.514049587 | 16.13 |
| POLDIP3 | Polymerase delta-interacting protein 3 OS=Homo sapiens OX=9606 GN=POLDIP3 PE=1 SV=2 | 0.352066116 | 16.09 |
| SERPINH1 | Serpin H1 OS=Homo sapiens OX=9606 GN=SERPINH1 PE=1 SV=2 | 0.254545455 | 15.89 |
| TXN | Thioredoxin OS=Homo sapiens OX=9606 GN=TXN PE=1 SV=3 | 0.684297521 | 15.64 |
| PTMAP7 | Prothymosin, alpha OS=Homo sapiens OX=9606 GN=PTMAP7 PE=2 SV=1 | 8.647933884 | 15.58 |
| NSA2 | Ribosome biogenesis protein NSA2 homolog OS=Homo sapiens OX=9606 GN=NSA2 PE=1 SV=1 | 1.34214876 | 15.57 |
| UTP6 | U3 small nucleolar RNA-associated protein 6 homolog OS=Homo sapiens OX=9606 GN=UTP6 PE=2 SV=2 | 1.958677686 | 15.18 |
| PCDHB2 | Protocadherin beta-2 OS=Homo sapiens OX=9606 GN=PCDHB2 PE=1 SV=1 | 4.12231405 | 15.17 |
| WDR12 | Ribosome biogenesis protein WDR12 OS=Homo sapiens OX=9606 GN=WDR12 PE=2 SV=1 | 0.525619835 | 15.16 |
| ATP2A2 | ATPase Ca++ transporting cardiac muscle slow twitch 2 isoform 1 (Fragment) OS=Homo sapiens OX=9606 GN=ATP2A2 PE=2 SV=1 | 1.231404959 | 15.1 |
| SNRPB2 | Small nuclear ribonucleoprotein polypeptide B'' OS=Homo sapiens OX=9606 GN=SNRPB2 PE=2 SV=1 | 1.104132231 | 14.89 |
| SFXN1 | Sideroflexin-1 OS=Homo sapiens OX=9606 GN=SFXN1 PE=1 SV=4 | 0.869421488 | 14.59 |
| KPNA6 | Importin subunit alpha-7 OS=Homo sapiens OX=9606 GN=KPNA6 PE=1 SV=1 | 2.272727273 | 14.4 |
| RBBP7 | Histone-binding protein RBBP7 OS=Homo sapiens OX=9606 GN=RBBP7 PE=1 SV=1 | 0.714049587 | 14.36 |
| BRIX1 | Ribosome biogenesis protein BRX1 homolog OS=Homo sapiens OX=9606 GN=BRIX1 PE=1 SV=2 | 0.312396694 | 13.98 |
| RBM15 | RNA-binding protein 15 OS=Homo sapiens OX=9606 GN=RBM15 PE=4 SV=1 | 1.538842975 | 13.71 |
| EIF3D | Eukaryotic translation initiation factor 3 subunit D OS=Homo sapiens OX=9606 GN=EIF3D PE=1 SV=1 | 2.638016529 | 13.66 |
| RPS24 | 40S ribosomal protein S24 OS=Homo sapiens OX=9606 GN=RPS24 PE=1 SV=1 | 2.380165289 | 13.65 |
| CDK1 | Cyclin-dependent kinase 1 OS=Homo sapiens OX=9606 GN=CDK1 PE=1 SV=3 | 1.444628099 | 13.63 |
| NSUN2 | tRNA (cytosine(34)-C(5))-methyltransferase OS=Homo sapiens OX=9606 GN=NSUN2 PE=1 SV=2 | 1.314049587 | 13.54 |
| RPS19BP1 | Ribosomal protein S19 binding protein 1, isoform CRA_a OS=Homo sapiens OX=9606 GN=RPS19BP1 PE=4 SV=1 | 3.132231405 | 13.51 |
| RPL7L1 | 60S ribosomal protein L7-like 1 OS=Homo sapiens OX=9606 GN=RPL7L1 PE=1 SV=2 | 1.894214876 | 13.44 |
| PAK1IP1 | p21-activated protein kinase-interacting protein 1 OS=Homo sapiens OX=9606 GN=PAK1IP1 PE=1 SV=2 | 0.639669421 | 13.25 |
| PGRMC1 | PGRMC1 protein OS=Homo sapiens OX=9606 GN=PGRMC1 PE=2 SV=1 | 0.798347107 | 13.15 |
| ANXA2 | Annexin A2 OS=Homo sapiens OX=9606 GN=ANXA2 PE=1 SV=2 | 1.117355372 | 13.03 |
| PBEF1 | Nicotinamide phosphoribosyltransferase OS=Homo sapiens OX=9606 GN=PBEF1 PE=3 SV=1 | 0.226446281 | 13.03 |
| TMEM70 | Alternative protein TMEM70 OS=Homo sapiens OX=9606 GN=TMEM70 PE=4 SV=1 | 3.330578512 | 13 |
| TUBAL3 | Tubulin alpha chain-like 3 OS=Homo sapiens OX=9606 GN=TUBAL3 PE=1 SV=2 | 0.646280992 | 13 |
| SERPINB12 | Serpin B12 OS=Homo sapiens OX=9606 GN=SERPINB12 PE=1 SV=1 | 0.272727273 | 12.5 |
| MGST1 | Microsomal glutathione S-transferase 1 (Fragment) OS=Homo sapiens OX=9606 GN=MGST1 PE=1 SV=1 | 2.251239669 | 12.12 |
| HDGFRP3 | Hepatoma-derived growth factor, related protein 3, isoform CRA_a OS=Homo sapiens OX=9606 GN=HDGFRP3 PE=1 SV=1 | 2.550413223 | 12.02 |
| ANKRD11 | Alternative protein ANKRD11 OS=Homo sapiens OX=9606 GN=ANKRD11 PE=4 SV=1 | 1.47107438 | 11.96 |
| VDAC3 | Voltage-dependent anion-selective channel protein 3 OS=Homo sapiens OX=9606 GN=VDAC3 PE=1 SV=1 | 1.441322314 | 11.88 |
| RPA1 | Replication protein A 70 kDa DNA-binding subunit OS=Homo sapiens OX=9606 GN=RPA1 PE=1 SV=2 | 4.038016529 | 11.76 |
| CACYBP | Calcyclin-binding protein OS=Homo sapiens OX=9606 GN=CACYBP PE=1 SV=2 | 1.869421488 | 11.73 |
| CST1 | Cystatin-SN OS=Homo sapiens OX=9606 GN=CST1 PE=1 SV=3 | 0.016528926 | 11.68 |
| TBL3 | Transducin beta-like protein 3 (Fragment) OS=Homo sapiens OX=9606 GN=TBL3 PE=1 SV=1 | 1.292561983 | 11.51 |
| RAB5A | RAB5A, member RAS oncogene family, isoform CRA_a OS=Homo sapiens OX=9606 GN=RAB5A PE=4 SV=1 | 1.495867769 | 11.46 |
| SHINC3 | SHINC3 OS=Homo sapiens OX=9606 GN=SHINC3 PE=2 SV=1 | 0.573553719 | 11.31 |
| ARG1 | Arginase-1 OS=Homo sapiens OX=9606 GN=ARG1 PE=1 SV=2 | 0.995041322 | 11.14 |
| CHD1 | Chromodomain-helicase-DNA-binding protein 1 OS=Homo sapiens OX=9606 GN=CHD1 PE=1 SV=2 | 4.826446281 | 11.08 |
| MTF2 | Metal-response element-binding transcription factor 2 OS=Homo sapiens OX=9606 GN=MTF2 PE=1 SV=3 | 2.85785124 | 11.08 |
| RRP1 | RRP1 protein (Fragment) OS=Homo sapiens OX=9606 GN=RRP1 PE=2 SV=2 | 0.447933884 | 11.08 |
| UTP3 | Something about silencing protein 10 OS=Homo sapiens OX=9606 GN=UTP3 PE=1 SV=1 | 1.975206612 | 11.05 |
| GPATCH4 | G patch domain-containing protein 4 OS=Homo sapiens OX=9606 GN=GPATCH4 PE=1 SV=2 | 1.469421488 | 10.99 |
| IMP3 | U3 small nucleolar ribonucleoprotein protein IMP3 OS=Homo sapiens OX=9606 GN=IMP3 PE=1 SV=1 | 0.692561983 | 10.92 |
| HMGB3 | High mobility group protein B3 OS=Homo sapiens OX=9606 GN=HMGB3 PE=1 SV=4 | 1.442975207 | 10.89 |
| WDR3 | WD repeat-containing protein 3 OS=Homo sapiens OX=9606 GN=WDR3 PE=1 SV=1 | 1.580165289 | 10.88 |
| NOC3L | Nucleolar complex protein 3 homolog OS=Homo sapiens OX=9606 GN=NOC3L PE=1 SV=1 | 1.325619835 | 10.87 |
| SKP1 | S-phase kinase-associated protein 1 OS=Homo sapiens OX=9606 GN=SKP1 PE=1 SV=1 | 0.919008264 | 10.78 |
| DDX54 | ATP-dependent RNA helicase DDX54 OS=Homo sapiens OX=9606 GN=DDX54 PE=1 SV=2 | 1.366942149 | 10.73 |
| KPNA1 | Importin subunit alpha OS=Homo sapiens OX=9606 GN=KPNA1 PE=2 SV=1 | 2.304132231 | 10.63 |
| CAT | Catalase OS=Homo sapiens OX=9606 GN=CAT PE=1 SV=3 | 0.803305785 | 10.61 |
| NUCKS1 | Nuclear ubiquitous casein and cyclin-dependent kinase substrate 1 OS=Homo sapiens OX=9606 GN=NUCKS1 PE=1 SV=1 | 1.633057851 | 10.5 |
| PUM3 | Pumilio homolog 3 OS=Homo sapiens OX=9606 GN=PUM3 PE=1 SV=3 | 2.190082645 | 10.4 |
| SNRPD1 | Small nuclear ribonucleoprotein Sm D1 OS=Homo sapiens OX=9606 GN=SNRPD1 PE=1 SV=1 | 3.608264463 | 10.37 |
| MTDH | Metadherin, isoform CRA_a OS=Homo sapiens OX=9606 GN=MTDH PE=4 SV=1 | 1.236363636 | 10.15 |
| SLC1A5 | Neutral amino acid transporter B(0) OS=Homo sapiens OX=9606 GN=SLC1A5 PE=1 SV=2 | 1.353719008 | 10.09 |
| RARS1 | Arginine--tRNA ligase, cytoplasmic OS=Homo sapiens OX=9606 GN=RARS PE=1 SV=2 | 1.783471074 | 10.02 |
| DDOST | Dolichyl-diphosphooligosaccharide--protein glycosyltransferase 48 kDa subunit OS=Homo sapiens OX=9606 GN=DDOST PE=1 SV=4 | 0.476033058 | 9.99 |
| APOD | Apolipoprotein D (Fragment) OS=Homo sapiens OX=9606 GN=APOD PE=1 SV=1 | 165.2892562 | 9.82 |
| NOL7 | Nucleolar protein 7, 27kDa, isoform CRA_a OS=Homo sapiens OX=9606 GN=NOL7 PE=4 SV=1 | 1.910743802 | 9.72 |
| BRWD1 | Bromodomain and WD repeat-containing protein 1 OS=Homo sapiens OX=9606 GN=BRWD1 PE=1 SV=4 | 3.487603306 | 9.71 |
| LARP7 | La-related protein 7 OS=Homo sapiens OX=9606 GN=LARP7 PE=1 SV=1 | 1.46446281 | 9.7 |
| CHAF1A | Chromatin assembly factor 1 subunit A OS=Homo sapiens OX=9606 GN=CHAF1A PE=1 SV=3 | 1.652892562 | 9.6 |
| RRP9 | U3 small nucleolar RNA-interacting protein 2 OS=Homo sapiens OX=9606 GN=RRP9 PE=1 SV=1 | 0.525619835 | 9.53 |
| TRIM21 | E3 ubiquitin-protein ligase TRIM21 OS=Homo sapiens OX=9606 GN=TRIM21 PE=1 SV=1 | 0.338842975 | 9.49 |
| MAGT1 | Magnesium transporter protein 1 OS=Homo sapiens OX=9606 GN=MAGT1 PE=1 SV=1 | 1.715702479 | 9.36 |
| MRPS22 | 28S ribosomal protein S22, mitochondrial OS=Homo sapiens OX=9606 GN=MRPS22 PE=1 SV=1 | 2.234710744 | 9.3 |
| MRPS23 | 28S ribosomal protein S23, mitochondrial OS=Homo sapiens OX=9606 GN=MRPS23 PE=1 SV=1 | 1.385123967 | 9.27 |
| USP39 | U4/U6.U5 tri-snRNP-associated protein 2 OS=Homo sapiens OX=9606 GN=USP39 PE=1 SV=2 | 1.760330579 | 9.25 |
| MRTO4 | mRNA turnover protein 4 homolog OS=Homo sapiens OX=9606 GN=MRTO4 PE=1 SV=2 | 0.988429752 | 9.23 |
| XRCC1 | X-ray repair complementing defective repair in Chinese hamster cells 1, isoform CRA_b OS=Homo sapiens OX=9606 GN=XRCC1 PE=4 SV=1 | 4.340495868 | 9.02 |
| GRWD1 | Glutamate-rich WD repeat-containing protein 1 OS=Homo sapiens OX=9606 GN=GRWD1 PE=1 SV=1 | 0.404958678 | 9 |
| SF3B2 | Splicing factor 3B subunit 2 OS=Homo sapiens OX=9606 GN=SF3B2 PE=1 SV=2 | 1.150413223 | 8.86 |
| WDR76 | WD repeat-containing protein 76 OS=Homo sapiens OX=9606 GN=WDR76 PE=1 SV=2 | 4.565289256 | 8.85 |
| SRM | Spermidine synthase OS=Homo sapiens OX=9606 GN=SRM PE=1 SV=1 | 1.980165289 | 8.82 |
| CIT | Citron Rho-interacting kinase OS=Homo sapiens OX=9606 GN=CIT PE=1 SV=2 | 0.67768595 | 8.81 |
| TRMT10C | tRNA methyltransferase 10 homolog C OS=Homo sapiens OX=9606 GN=TRMT10C PE=1 SV=2 | 0.229752066 | 8.75 |
| SRP14 | Signal recognition particle 14 kDa protein OS=Homo sapiens OX=9606 GN=SRP14 PE=1 SV=2 | 2.510743802 | 8.66 |
| RPF2 | Ribosome production factor 2 homolog OS=Homo sapiens OX=9606 GN=RPF2 PE=1 SV=2 | 1.532231405 | 8.42 |
| STT3A | Dolichyl-diphosphooligosaccharide--protein glycosyltransferase subunit STT3A OS=Homo sapiens OX=9606 GN=STT3A PE=1 SV=2 | 1.621487603 | 8.39 |
| HMGN2 | Non-histone chromosomal protein HMG-17 OS=Homo sapiens OX=9606 GN=HMGN2 PE=1 SV=3 | 1.765289256 | 8.06 |
| PLXNA2 | Plexin-A2 OS=Homo sapiens OX=9606 GN=PLXNA2 PE=1 SV=4 | 1.487603306 | 8.02 |
| VDAC1 | Voltage-dependent anion-selective channel protein 1 OS=Homo sapiens OX=9606 GN=VDAC1 PE=1 SV=2 | 2.13553719 | 8.01 |
| PWP2 | Periodic tryptophan protein 2 homolog OS=Homo sapiens OX=9606 GN=PWP2 PE=2 SV=2 | 1.487603306 | 7.78 |
| KRR1 | KRR1 small subunit processome component homolog OS=Homo sapiens OX=9606 GN=KRR1 PE=1 SV=4 | 0.694214876 | 7.73 |
| HDAC1 | Histone deacetylase OS=Homo sapiens OX=9606 GN=HDAC1 PE=2 SV=1 | 2.054545455 | 7.71 |
| HDAC2 | Histone deacetylase 2 OS=Homo sapiens OX=9606 GN=HDAC2 PE=1 SV=2 | 2.27107438 | 7.71 |
| NOL10 | Nucleolar protein 10 OS=Homo sapiens OX=9606 GN=NOL10 PE=1 SV=1 | 1.690909091 | 7.7 |
| STON2 | Stonin-2 OS=Homo sapiens OX=9606 GN=STON2 PE=1 SV=1 | 3.462809917 | 7.6 |
| EPRS1 | Bifunctional glutamate/proline--tRNA ligase OS=Homo sapiens OX=9606 GN=EPRS PE=1 SV=5 | 1.254545455 | 7.56 |
| GATAD2B | Transcriptional repressor p66-beta OS=Homo sapiens OX=9606 GN=GATAD2B PE=1 SV=1 | 2.241322314 | 7.56 |
| FLG | Truncated profilaggrin OS=Homo sapiens OX=9606 GN=FLG PE=4 SV=1 | 0.499173554 | 7.48 |
| CST2 | Cystatin-SA OS=Homo sapiens OX=9606 GN=CST2 PE=1 SV=1 | 0.016528926 | 7.44 |
| MYL6 | Myosin light polypeptide 6 OS=Homo sapiens OX=9606 GN=MYL6 PE=1 SV=1 | 5.094214876 | 7.27 |
| AHSA1 | Activator of 90 kDa heat shock protein ATPase homolog 1 OS=Homo sapiens OX=9606 GN=AHSA1 PE=1 SV=1 | 0.401652893 | 7.21 |
| RBM28 | RNA binding motif protein 28 isoform 1 OS=Homo sapiens OX=9606 GN=RBM28 PE=2 SV=1 | 1.099173554 | 7.2 |
| MTA2 | Metastasis associated 1 family, member 2, isoform CRA_a OS=Homo sapiens OX=9606 GN=MTA2 PE=4 SV=1 | 2.403305785 | 6.98 |
| KHSRP | Far upstream element-binding protein 2 OS=Homo sapiens OX=9606 GN=KHSRP PE=1 SV=4 | 1.500826446 | 6.96 |
| COPA | Coatomer subunit alpha OS=Homo sapiens OX=9606 GN=COPA PE=1 SV=2 | 1.195041322 | 6.94 |
| EMG1 | Ribosomal RNA small subunit methyltransferase NEP1 OS=Homo sapiens OX=9606 GN=EMG1 PE=1 SV=4 | 1.27768595 | 6.9 |
| DHX30 | ATP-dependent RNA helicase DHX30 OS=Homo sapiens OX=9606 GN=DHX30 PE=1 SV=1 | 1.196694215 | 6.87 |
| CSF2RB | Alternative protein CSF2RB OS=Homo sapiens OX=9606 GN=CSF2RB PE=4 SV=1 | 1.429752066 | 6.79 |
| SEC11A | Signal peptidase complex catalytic subunit SEC11 (Fragment) OS=Homo sapiens OX=9606 GN=SEC11A PE=1 SV=1 | 3.656198347 | 6.79 |
| SRPK1 | SFRS protein kinase 1, isoform CRA_e OS=Homo sapiens OX=9606 GN=SRPK1 PE=4 SV=1 | 0.484297521 | 6.78 |
| TGM3 | Protein-glutamine gamma-glutamyltransferase E OS=Homo sapiens OX=9606 GN=TGM3 PE=1 SV=4 | 0.689256198 | 6.75 |
| WDR57 | CDW11/WDR57 OS=Homo sapiens OX=9606 GN=WDR57 PE=2 SV=1 | 1.646280992 | 6.64 |
| RPS10 | 40S ribosomal protein S10 OS=Homo sapiens OX=9606 GN=RPS10 PE=1 SV=1 | 1.6 | 6.27 |
| MARS1 | Methionine--tRNA ligase, cytoplasmic OS=Homo sapiens OX=9606 GN=MARS PE=1 SV=2 | 1.276033058 | 6.08 |
| RBM8A | RNA-binding protein 8A OS=Homo sapiens OX=9606 GN=RBM8A PE=1 SV=1 | 0.649586777 | 6.05 |
| NOL6 | Nucleolar protein 6 OS=Homo sapiens OX=9606 GN=NOL6 PE=1 SV=2 | 1.221487603 | 6.02 |
| SNRPA | U1 small nuclear ribonucleoprotein A OS=Homo sapiens OX=9606 GN=SNRPA PE=1 SV=3 | 2.176859504 | 5.93 |
| ABCB7 | ATP-binding cassette sub-family B member 7 isoform 1 (Fragment) OS=Homo sapiens OX=9606 GN=ABCB7 PE=2 SV=1 | 1.709090909 | 5.86 |
| FASN | Fatty acid synthase OS=Homo sapiens OX=9606 GN=FASN PE=1 SV=3 | 4.229752066 | 5.86 |
| DNAJA3 | DnaJ homolog subfamily A member 3, mitochondrial OS=Homo sapiens OX=9606 GN=DNAJA3 PE=1 SV=2 | 1.246280992 | 5.85 |
| WDR5 | WD repeat-containing protein 5 OS=Homo sapiens OX=9606 GN=WDR5 PE=1 SV=1 | 2.550413223 | 5.85 |
| HEATR1 | HEAT repeat containing 1 OS=Homo sapiens OX=9606 GN=HEATR1 PE=2 SV=1 | 2.089256198 | 5.83 |
| NIP7 | 60S ribosome subunit biogenesis protein NIP7 homolog OS=Homo sapiens OX=9606 GN=NIP7 PE=1 SV=1 | 0.451239669 | 5.82 |
| LUC7L2 | Putative RNA-binding protein Luc7-like 2 OS=Homo sapiens OX=9606 GN=LUC7L2 PE=1 SV=2 | 0.446280992 | 5.77 |
| RECQL | ATP-dependent DNA helicase Q1 OS=Homo sapiens OX=9606 GN=RECQL PE=1 SV=3 | 2.540495868 | 5.77 |
| IMP4 | U3 small nucleolar ribonucleoprotein protein IMP4 (Fragment) OS=Homo sapiens OX=9606 GN=IMP4 PE=1 SV=1 | 1.261157025 | 5.76 |
| SAP18 | Histone deacetylase complex subunit SAP18 OS=Homo sapiens OX=9606 GN=SAP18 PE=1 SV=1 | 0.884297521 | 5.75 |
| GFPT1 | Glutamine--fructose-6-phosphate aminotransferase [isomerizing] 1 OS=Homo sapiens OX=9606 GN=GFPT1 PE=1 SV=3 | 1.925619835 | 5.68 |
| SMCHD1 | Structural maintenance of chromosomes flexible hinge domain-containing protein 1 OS=Homo sapiens OX=9606 GN=SMCHD1 PE=1 SV=2 | 3.305785124 | 5.66 |
| PSIP1 | PC4 and SFRS1-interacting protein (Fragment) OS=Homo sapiens OX=9606 GN=PSIP1 PE=1 SV=1 | 0.566942149 | 5.63 |
| CAD | CAD protein OS=Homo sapiens OX=9606 GN=CAD PE=1 SV=3 | 3.758677686 | 5.52 |
| LRRC40 | Leucine-rich repeat-containing protein 40 OS=Homo sapiens OX=9606 GN=LRRC40 PE=1 SV=1 | 1.753719008 | 5.42 |
| CBX8 | Chromobox protein homolog 8 OS=Homo sapiens OX=9606 GN=CBX8 PE=1 SV=3 | 1.024793388 | 5.4 |
| PLG | Plasminogen OS=Homo sapiens OX=9606 GN=PLG PE=2 SV=1 | 2.861157025 | 5.39 |
| SNRPD2 | Small nuclear ribonucleoprotein Sm D2 OS=Homo sapiens OX=9606 GN=SNRPD2 PE=1 SV=1 | 5.07107438 | 5.38 |
| DNAJA1 | DnaJ homolog subfamily A member 1 OS=Homo sapiens OX=9606 GN=DNAJA1 PE=1 SV=2 | 0.707438017 | 5.34 |
| WDR36 | WD repeat-containing protein 36 OS=Homo sapiens OX=9606 GN=WDR36 PE=1 SV=1 | 1.097520661 | 5.32 |
| ZC3H13 | Zinc finger CCCH domain-containing protein 13 OS=Homo sapiens OX=9606 GN=ZC3H13 PE=1 SV=1 | 4.732231405 | 5.29 |
| RRP8 | Ribosomal RNA-processing protein 8 OS=Homo sapiens OX=9606 GN=RRP8 PE=1 SV=2 | 2.158677686 | 5.27 |
| UQCRFS1P1 | Putative cytochrome b-c1 complex subunit Rieske-like protein 1 OS=Homo sapiens OX=9606 GN=UQCRFS1P1 PE=5 SV=1 | 1.046280992 | 5.27 |
| YTHDC1 | YTH domain-containing protein 1 OS=Homo sapiens OX=9606 GN=YTHDC1 PE=1 SV=1 | 2.085950413 | 5.27 |
| PRKDC | DNA-dependent protein kinase catalytic subunit OS=Homo sapiens OX=9606 GN=PRKDC PE=1 SV=3 | 2.424793388 | 5.26 |
| DCAF13 | DDB1- and CUL4-associated factor 13 OS=Homo sapiens OX=9606 GN=DCAF13 PE=1 SV=1 | 0.614876033 | 5.23 |
| CHCHD3 | MICOS complex subunit OS=Homo sapiens OX=9606 GN=CHCHD3 PE=1 SV=2 | 1.244628099 | 5.19 |
| NRF | NF-kappaB repressing factor OS=Homo sapiens OX=9606 GN=NRF PE=2 SV=1 | 1.880991736 | 5.16 |
| RAB1A | H.sapiens ras-related Hrab1A protein OS=Homo sapiens OX=9606 GN=RAB1A PE=2 SV=1 | 0.80661157 | 5.14 |
| LMNA | Lamin A/C OS=Homo sapiens OX=9606 GN=LMNA PE=3 SV=1 | 1.909090909 | 5.11 |
| LMNB2 | Lamin-B2 OS=Homo sapiens OX=9606 GN=LMNB2 PE=1 SV=4 | 1 | 5.11 |
| GNAI1 | Guanine nucleotide-binding protein G(i) subunit alpha-1 OS=Homo sapiens OX=9606 GN=GNAI1 PE=1 SV=1 | 1.58677686 | 5.1 |
| LTF | Lactoferrin OS=Homo sapiens OX=9606 GN=LTF PE=2 SV=1 | 0.312396694 | 5.03 |
| COIL | Coilin OS=Homo sapiens OX=9606 GN=COIL PE=1 SV=1 | 1.467768595 | 5.01 |
| EXOSC3 | Exosome complex component RRP40 OS=Homo sapiens OX=9606 GN=EXOSC3 PE=1 SV=3 | 1.709090909 | 5 |
| NDUFA13 | NADH dehydrogenase [ubiquinone] 1 alpha subcomplex subunit 13 OS=Homo sapiens OX=9606 GN=NDUFA13 PE=1 SV=3 | 0.978512397 | 4.69 |
| HEL-S-100n | Chaperonin containing TCP1, subunit 2 (Beta), isoform CRA_b OS=Homo sapiens OX=9606 GN=HEL-S-100n PE=2 SV=1 | 2.963636364 | 4.68 |
| DMAP1 | DNA methyltransferase 1-associated protein 1 OS=Homo sapiens OX=9606 GN=DMAP1 PE=1 SV=1 | 2.861157025 | 4.53 |
| SPOUT1 | Putative methyltransferase C9orf114 OS=Homo sapiens OX=9606 GN=SPOUT1 PE=1 SV=3 | 0.737190083 | 4.52 |
| MRPL16 | 39S ribosomal protein L16, mitochondrial OS=Homo sapiens OX=9606 GN=MRPL16 PE=1 SV=1 | 0.771900826 | 4.39 |
| NHP2 | H/ACA ribonucleoprotein complex subunit 2 OS=Homo sapiens OX=9606 GN=NHP2 PE=1 SV=1 | 2.555371901 | 4.39 |
| CHD4 | Chromodomain-helicase-DNA-binding protein 4 OS=Homo sapiens OX=9606 GN=CHD4 PE=1 SV=1 | 3.27768595 | 4.38 |
| HMGB1 | High mobility group protein B1 OS=Homo sapiens OX=9606 GN=HMGB1 PE=1 SV=1 | 1.828099174 | 4.37 |
| HSD17B10 | Hydroxysteroid dehydrogenase 10 isoform 2 OS=Homo sapiens OX=9606 GN=HSD17B10 PE=2 SV=1 | 2.085950413 | 4.34 |
| PPHLN1 | Periphilin-1 OS=Homo sapiens OX=9606 GN=PPHLN1 PE=1 SV=1 | 4.107438017 | 4.27 |
| RNF2 | E3 ubiquitin-protein ligase RING2 OS=Homo sapiens OX=9606 GN=RNF2 PE=1 SV=1 | 3.433057851 | 4.13 |
| RPP30 | Ribonuclease P protein subunit p30 (Fragment) OS=Homo sapiens OX=9606 GN=RPP30 PE=1 SV=1 | 1.40661157 | 4.12 |
| LTV1 | Protein LTV1 homolog OS=Homo sapiens OX=9606 GN=LTV1 PE=1 SV=1 | 2.127272727 | 4.1 |
| C5orf24 | UPF0461 protein C5orf24 OS=Homo sapiens OX=9606 GN=C5orf24 PE=1 SV=1 | 1.626446281 | 4.08 |
| ING5 | Inhibitor of growth protein 5 OS=Homo sapiens OX=9606 GN=ING5 PE=1 SV=1 | 1.267768595 | 4.07 |
| KPNA3 | Importin subunit alpha-4 OS=Homo sapiens OX=9606 GN=KPNA3 PE=1 SV=2 | 1.515702479 | 4.05 |
| N-PAC | Cytokine-like nuclear factor n-pac, isoform CRA_c OS=Homo sapiens OX=9606 GN=N-PAC PE=4 SV=1 | 1.874380165 | 4.02 |
| LARP1 | La-related protein 1 OS=Homo sapiens OX=9606 GN=LARP1 PE=1 SV=2 | 1.948760331 | 3.96 |
| BMS1 | Ribosome biogenesis protein BMS1 homolog OS=Homo sapiens OX=9606 GN=BMS1 PE=1 SV=1 | 1.444628099 | 3.93 |
| JCHAIN | Immunoglobulin J chain (Fragment) OS=Homo sapiens OX=9606 GN=JCHAIN PE=1 SV=1 | 0.816528926 | 3.87 |
| CDC73 | Parafibromin OS=Homo sapiens OX=9606 GN=CDC73 PE=1 SV=1 | 2.114049587 | 3.85 |
| MRI1 | Methylthioribose-1-phosphate isomerase OS=Homo sapiens OX=9606 GN=MRI1 PE=1 SV=1 | 0.558677686 | 3.81 |
| HSPB7 | Heat shock 27kDa protein family, member 7 (Cardiovascular), isoform CRA_c OS=Homo sapiens OX=9606 GN=HSPB7 PE=1 SV=1 | 0.568595041 | 3.8 |
| DSTN | Destrin OS=Homo sapiens OX=9606 GN=DSTN PE=1 SV=1 | 4.585123967 | 3.79 |
| WDR74 | WD repeat-containing protein 74 (Fragment) OS=Homo sapiens OX=9606 GN=WDR74 PE=1 SV=1 | 0.6 | 3.77 |
| AAAS | Aladin OS=Homo sapiens OX=9606 GN=AAAS PE=1 SV=1 | 3.153719008 | 3.73 |
| AFG3L2 | AFG3-like protein 2 OS=Homo sapiens OX=9606 GN=AFG3L2 PE=1 SV=2 | 1.791735537 | 3.67 |
| RCL1 | RNA 3'-terminal phosphate cyclase-like protein OS=Homo sapiens OX=9606 GN=RCL1 PE=1 SV=3 | 2.238016529 | 3.67 |
| BCAS2 | Breast carcinoma amplified sequence 2 OS=Homo sapiens OX=9606 GN=BCAS2 PE=2 SV=1 | 1.026446281 | 3.66 |
| RACGAP1 | Rac GTPase activating protein 1, isoform CRA_a OS=Homo sapiens OX=9606 GN=RACGAP1 PE=4 SV=1 | 2.370247934 | 3.64 |
| DNAJA2 | DnaJ homolog subfamily A member 2 OS=Homo sapiens OX=9606 GN=DNAJA2 PE=1 SV=1 | 0.771900826 | 3.6 |
| CHD1L | Chromodomain-helicase-DNA-binding protein 1-like OS=Homo sapiens OX=9606 GN=CHD1L PE=1 SV=2 | 3.19338843 | 3.57 |
| MTA1 | Metastasis-associated protein MTA1 OS=Homo sapiens OX=9606 GN=MTA1 PE=1 SV=1 | 2.085950413 | 3.57 |
| TRIM27 | Zinc finger protein RFP OS=Homo sapiens OX=9606 GN=TRIM27 PE=1 SV=1 | 2.945454545 | 3.57 |
| PSMA3 | Proteasome subunit alpha type-3 OS=Homo sapiens OX=9606 GN=PSMA3 PE=1 SV=2 | 1.039669421 | 3.55 |
| PAICS | Multifunctional protein ADE2 (Fragment) OS=Homo sapiens OX=9606 GN=PAICS PE=1 SV=1 | 1.280991736 | 3.54 |
| CLTC | Clathrin heavy chain OS=Homo sapiens OX=9606 GN=CLTC PE=1 SV=1 | 1.570247934 | 3.53 |
| RAB14 | Ras-related protein Rab-14 OS=Homo sapiens OX=9606 GN=RAB14 PE=1 SV=4 | 1.12892562 | 3.53 |
| CBR1 | Carbonyl reductase [NADPH] 1 OS=Homo sapiens OX=9606 GN=CBR1 PE=1 SV=3 | 1.320661157 | 3.51 |
| NGDN | Neuroguidin OS=Homo sapiens OX=9606 GN=NGDN PE=1 SV=1 | 1.355371901 | 3.51 |
| PHF6 | PHD finger protein 6 OS=Homo sapiens OX=9606 GN=PHF6 PE=1 SV=1 | 0.190082645 | 3.47 |
| CENPB | Major centromere autoantigen B OS=Homo sapiens OX=9606 GN=CENPB PE=1 SV=2 | 3.414876033 | 3.44 |
| HADHB | Trifunctional enzyme subunit beta, mitochondrial OS=Homo sapiens OX=9606 GN=HADHB PE=1 SV=3 | 0.66446281 | 3.43 |
| IARS1 | Isoleucine--tRNA ligase, cytoplasmic OS=Homo sapiens OX=9606 GN=IARS PE=1 SV=1 | 1.514049587 | 3.42 |
| KNOP1 | Lysine-rich nucleolar protein 1 OS=Homo sapiens OX=9606 GN=KNOP1 PE=1 SV=1 | 1.66446281 | 3.42 |
| MDH2 | Malate dehydrogenase OS=Homo sapiens OX=9606 GN=MDH2 PE=2 SV=1 | 3.411570248 | 3.38 |
| MRPS15 | 28S ribosomal protein S15, mitochondrial OS=Homo sapiens OX=9606 GN=MRPS15 PE=1 SV=1 | 0.991735537 | 3.35 |
| MRPS2 | Mitochondrial ribosomal protein S2, isoform CRA_a OS=Homo sapiens OX=9606 GN=MRPS2 PE=3 SV=1 | 0.783471074 | 3.35 |
| KLHL22 | Kelch-like protein 22 OS=Homo sapiens OX=9606 GN=KLHL22 PE=1 SV=2 | 2.052892562 | 3.33 |
| SLC25A1 | Plasma membrane citrate carrier OS=Homo sapiens OX=9606 GN=SLC25A1 PE=2 SV=1 | 0.656198347 | 3.33 |
| MRPL9 | 39S ribosomal protein L9, mitochondrial OS=Homo sapiens OX=9606 GN=MRPL9 PE=1 SV=1 | 1.219834711 | 3.32 |
| NCBP1 | Nuclear cap-binding protein subunit 1 OS=Homo sapiens OX=9606 GN=NCBP1 PE=1 SV=1 | 1.540495868 | 3.32 |
| SUV39H1 | Histone-lysine N-methyltransferase SUV39H1 OS=Homo sapiens OX=9606 GN=SUV39H1 PE=1 SV=1 | 0.866115702 | 3.31 |
| CAPRIN1 | Caprin-1 OS=Homo sapiens OX=9606 GN=CAPRIN1 PE=1 SV=2 | 1.363636364 | 3.29 |
| HDGFL2 | Hepatoma-derived growth factor-related protein 2 OS=Homo sapiens OX=9606 GN=HDGFL2 PE=1 SV=1 | 2.527272727 | 3.26 |
| MGST3 | Microsomal glutathione S-transferase 3 OS=Homo sapiens OX=9606 GN=MGST3 PE=1 SV=1 | 1.157024793 | 3.21 |
| NSD2 | Histone-lysine N-methyltransferase NSD2 (Fragment) OS=Homo sapiens OX=9606 GN=NSD2 PE=1 SV=1 | 165.2892562 | 3.09 |
| MRPS17 | 28S ribosomal protein S17, mitochondrial (Fragment) OS=Homo sapiens OX=9606 GN=MRPS17 PE=1 SV=1 | 3.419834711 | 2.68 |
| NOC4L | Nucleolar complex protein 4 homolog OS=Homo sapiens OX=9606 GN=NOC4L PE=1 SV=1 | 2.059504132 | 2.66 |
| CSNK2B | Casein kinase II subunit beta OS=Homo sapiens OX=9606 GN=CSNK2B PE=2 SV=1 | 1.138842975 | 2.58 |
| PRB1 | Basic salivary proline-rich protein 1 OS=Homo sapiens OX=9606 GN=PRB1 PE=1 SV=1 | 165.2892562 | 2.54 |
| MRPL48 | 39S ribosomal protein L48, mitochondrial OS=Homo sapiens OX=9606 GN=MRPL48 PE=1 SV=2 | 0.649586777 | 2.43 |
| UTP14A | U3 small nucleolar RNA-associated protein 14 homolog A OS=Homo sapiens OX=9606 GN=UTP14A PE=1 SV=1 | 1.791735537 | 2.42 |
| MEAF6 | Chromatin modification-related protein MEAF6 OS=Homo sapiens OX=9606 GN=MEAF6 PE=1 SV=1 | 1.895867769 | 2.39 |
| IRS4 | Insulin receptor substrate 4 OS=Homo sapiens OX=9606 GN=IRS4 PE=1 SV=1 | 1.019834711 | 2.31 |
| NUDT21 | Cleavage and polyadenylation-specificity factor subunit 5 (Fragment) OS=Homo sapiens OX=9606 GN=NUDT21 PE=1 SV=8 | 1.249586777 | 2.3 |
| NUBPL | Iron-sulfur protein NUBPL (Fragment) OS=Homo sapiens OX=9606 GN=NUBPL PE=1 SV=1 | 1.614876033 | 2.28 |
| CLK2 | Dual-specificity protein kinase CLK2 OS=Homo sapiens OX=9606 GN=CLK2 PE=1 SV=1 | 2.13553719 | 2.25 |
| DGKK | Alternative protein DGKK OS=Homo sapiens OX=9606 GN=DGKK PE=4 SV=1 | 0.609917355 | 2.13 |
| BOD1L1 | Biorientation of chromosomes in cell division protein 1-like 1 OS=Homo sapiens OX=9606 GN=BOD1L1 PE=1 SV=2 | 0.016528926 | 2.11 |
| HMGA2 | HMGA2e' OS=Homo sapiens OX=9606 GN=HMGA2 PE=2 SV=1 | 3.778512397 | 2.1 |
| ZMYND11 | Zinc finger MYND domain-containing protein 11 OS=Homo sapiens OX=9606 GN=ZMYND11 PE=1 SV=2 | 3.016528926 | 2.1 |
| DDX10 | RNA helicase (Fragment) OS=Homo sapiens OX=9606 GN=DDX10 PE=2 SV=1 | 1.421487603 | 2.08 |
| EXOSC9 | Exosome complex component RRP45 OS=Homo sapiens OX=9606 GN=EXOSC9 PE=1 SV=1 | 1.623140496 | 2.06 |
| EXOSC2 | EXOSC2 protein (Fragment) OS=Homo sapiens OX=9606 GN=EXOSC2 PE=2 SV=1 | 0.80661157 | 2.05 |
| FIP1L1 | Pre-mRNA 3'-end-processing factor FIP1 OS=Homo sapiens OX=9606 GN=FIP1L1 PE=1 SV=1 | 2.421487603 | 2.05 |
| EIF3F | Eukaryotic translation initiation factor 3 subunit F OS=Homo sapiens OX=9606 GN=EIF3F PE=2 SV=1 | 0.669421488 | 2.02 |
| MOV10 | Helicase MOV-10 OS=Homo sapiens OX=9606 GN=MOV10 PE=1 SV=1 | 1.502479339 | 2.01 |
| MRPS35 | 28S ribosomal protein S35, mitochondrial OS=Homo sapiens OX=9606 GN=MRPS35 PE=1 SV=1 | 2.543801653 | 2 |
| PUF60 | Poly(U)-binding-splicing factor PUF60 (Fragment) OS=Homo sapiens OX=9606 GN=PUF60 PE=1 SV=1 | 1.973553719 | 2 |
| BUB3 | Mitotic checkpoint protein BUB3 OS=Homo sapiens OX=9606 GN=BUB3 PE=1 SV=1 | 1.317355372 | 1.96 |
| MCM3 | DNA helicase (Fragment) OS=Homo sapiens OX=9606 GN=MCM3 PE=2 SV=1 | 1.545454545 | 1.95 |
| NPM3 | Nucleoplasmin-3 OS=Homo sapiens OX=9606 GN=NPM3 PE=1 SV=3 | 0.117355372 | 1.93 |
| PDCD2 | Programmed cell death protein 2 OS=Homo sapiens OX=9606 GN=PDCD2 PE=1 SV=2 | 0.016528926 | 1.91 |
| GMPS | GMP synthase [glutamine-hydrolyzing] OS=Homo sapiens OX=9606 GN=GMPS PE=1 SV=1 | 1.952066116 | 1.9 |
| MMTAG2 | Multiple myeloma tumor-associated protein 2 OS=Homo sapiens OX=9606 GN=MMTAG2 PE=1 SV=1 | 2.266115702 | 1.9 |
| POP1 | Processing of 1, ribonuclease P/MRP subunit (S. cerevisiae) OS=Homo sapiens OX=9606 GN=POP1 PE=2 SV=1 | 0.899173554 | 1.88 |
| MPG | DNA-3-methyladenine glycosylase (Fragment) OS=Homo sapiens OX=9606 GN=MPG PE=1 SV=1 | 2.404958678 | 1.87 |
| ZNF346 | Zinc finger protein 346 OS=Homo sapiens OX=9606 GN=ZNF346 PE=1 SV=1 | 1.662809917 | 1.87 |
| DCLK2 | Serine/threonine-protein kinase DCLK2 OS=Homo sapiens OX=9606 GN=DCLK2 PE=1 SV=1 | 0.016528926 | 1.84 |
| EIF2AK2 | eIF2AK2 protein OS=Homo sapiens OX=9606 GN=EIF2AK2 PE=2 SV=1 | 1.436363636 | 1.84 |
| ARL1 | ADP-ribosylation factor-like protein 1 OS=Homo sapiens OX=9606 GN=ARL1 PE=1 SV=1 | 0.550413223 | 1.83 |
| EHD4 | EH domain-containing protein 4 OS=Homo sapiens OX=9606 GN=EHD4 PE=1 SV=1 | 1.442975207 | 1.83 |
| GALK1 | Galactokinase OS=Homo sapiens OX=9606 GN=GALK1 PE=1 SV=1 | 0.246280992 | 1.83 |
| MRPL22 | 39S ribosomal protein L22, mitochondrial OS=Homo sapiens OX=9606 GN=MRPL22 PE=1 SV=1 | 0.548760331 | 1.83 |
| DNAJC7 | DnaJ homolog subfamily C member 7 OS=Homo sapiens OX=9606 GN=DNAJC7 PE=1 SV=2 | 2.018181818 | 1.82 |
| MTCH2 | Mitochondrial carrier homolog 2 (Fragment) OS=Homo sapiens OX=9606 GN=MTCH2 PE=1 SV=8 | 1.112396694 | 1.82 |
| PPIL1 | Peptidyl-prolyl cis-trans isomerase-like 1 OS=Homo sapiens OX=9606 GN=PPIL1 PE=1 SV=1 | 4.375206612 | 1.82 |
| CROCC | CROCC protein (Fragment) OS=Homo sapiens OX=9606 GN=CROCC PE=2 SV=1 | 4.014876033 | 1.81 |
| SPIN4 | Spindlin-4 OS=Homo sapiens OX=9606 GN=SPIN4 PE=1 SV=1 | 165.2892562 | 1.81 |
| PRDX3 | Thioredoxin-dependent peroxide reductase, mitochondrial OS=Homo sapiens OX=9606 GN=PRDX3 PE=1 SV=3 | 0.61322314 | 1.8 |
| THYN1 | Thymocyte nuclear protein 1, isoform CRA_a OS=Homo sapiens OX=9606 GN=THYN1 PE=4 SV=1 | 2.720661157 | 1.8 |
| NCBP2 | Nuclear cap-binding protein subunit 2 OS=Homo sapiens OX=9606 GN=NCBP2 PE=1 SV=1 | 1.433057851 | 1.79 |
| SLC27A4 | Long-chain fatty acid transport protein 4 OS=Homo sapiens OX=9606 GN=SLC27A4 PE=1 SV=1 | 1.203305785 | 1.79 |
| CETN2 | Centrin-2 OS=Homo sapiens OX=9606 GN=CETN2 PE=1 SV=1 | 6.229752066 | 1.78 |
| EWSR1 | RNA-binding protein EWS OS=Homo sapiens OX=9606 GN=EWSR1 PE=1 SV=1 | 3.963636364 | 1.78 |
| NTPCR | Cancer-related nucleoside-triphosphatase OS=Homo sapiens OX=9606 GN=NTPCR PE=1 SV=1 | 0.639669421 | 1.78 |
| NUP155 | Nucleoporin 155kDa, isoform CRA_a OS=Homo sapiens OX=9606 GN=NUP155 PE=4 SV=1 | 1.40661157 | 1.78 |
| CIZ1 | Cip1-interacting zinc finger protein OS=Homo sapiens OX=9606 GN=CIZ1 PE=1 SV=2 | 2.689256198 | 1.77 |
| GLTSCR2 | GLTSCR2 protein (Fragment) OS=Homo sapiens OX=9606 GN=GLTSCR2 PE=2 SV=2 | 1.41322314 | 1.77 |
| RSF1 | Remodeling and spacing factor 1 OS=Homo sapiens OX=9606 GN=RSF1 PE=1 SV=2 | 3.178512397 | 1.77 |
| TTN | Titin OS=Homo sapiens OX=9606 GN=TTN PE=4 SV=1 | 1.360330579 | 1.77 |
| CCT7 | T-complex protein 1 subunit eta OS=Homo sapiens OX=9606 GN=CCT7 PE=1 SV=2 | 2.219834711 | 1.76 |
| DDX31 | Probable ATP-dependent RNA helicase DDX31 OS=Homo sapiens OX=9606 GN=DDX31 PE=1 SV=2 | 0.016528926 | 1.76 |
| FARSB | Phenylalanine--tRNA ligase beta subunit OS=Homo sapiens OX=9606 GN=FARSB PE=1 SV=3 | 1.826446281 | 1.76 |
| ARHGEF17 | Rho guanine nucleotide exchange factor 17 OS=Homo sapiens OX=9606 GN=ARHGEF17 PE=1 SV=1 | 1.079338843 | 1.75 |
| CEBPZ | CCAAT/enhancer-binding protein zeta OS=Homo sapiens OX=9606 GN=CEBPZ PE=1 SV=3 | 1.241322314 | 1.75 |
| OXA1L | Mitochondrial inner membrane protein OXA1L OS=Homo sapiens OX=9606 GN=OXA1L PE=1 SV=1 | 1.07107438 | 1.75 |
| HEL-S-282 | Epididymis secretory protein Li 282 OS=Homo sapiens OX=9606 GN=HEL-S-282 PE=2 SV=1 | 3.143801653 | 1.74 |
| SRPRB | Signal recognition particle receptor subunit beta OS=Homo sapiens OX=9606 GN=SRPRB PE=1 SV=3 | 1.596694215 | 1.74 |
| CHD2 | Chromodomain-helicase-DNA-binding protein 2 OS=Homo sapiens OX=9606 GN=CHD2 PE=1 SV=2 | 165.2892562 | 1.73 |
| HAX1 | HCLS1-associated protein X-1 OS=Homo sapiens OX=9606 GN=HAX1 PE=1 SV=2 | 1.490909091 | 1.73 |
| PFKP | ATP-dependent 6-phosphofructokinase, platelet type (Fragment) OS=Homo sapiens OX=9606 GN=PFKP PE=1 SV=1 | 0.818181818 | 1.73 |
| LCN1 | Lipocalin 1 (Tear prealbumin), isoform CRA_a OS=Homo sapiens OX=9606 GN=LCN1 PE=3 SV=1 | 0.616528926 | 1.72 |
| NUP205 | Nuclear pore complex protein Nup205 OS=Homo sapiens OX=9606 GN=NUP205 PE=1 SV=3 | 3.019834711 | 1.7 |
| TUBG1 | Tubulin gamma-1 chain OS=Homo sapiens OX=9606 GN=TUBG1 PE=1 SV=2 | 0.441322314 | 1.7 |
| PGRMC2 | Membrane-associated progesterone receptor component 2 OS=Homo sapiens OX=9606 GN=PGRMC2 PE=1 SV=1 | 0.824793388 | 1.69 |
| QPCTL | Glutaminyl-peptide cyclotransferase-like protein OS=Homo sapiens OX=9606 GN=QPCTL PE=1 SV=2 | 2.259504132 | 1.69 |
| COQ8A | Chaperone, ABC1 activity of bc1 complex like (S. pombe), isoform CRA_b OS=Homo sapiens OX=9606 GN=CABC1 PE=4 SV=1 | 0.097520661 | 1.68 |
| MARCKSL1 | MARCKS-related protein OS=Homo sapiens OX=9606 GN=MARCKSL1 PE=1 SV=2 | 1.294214876 | 1.67 |
| MKRN2 | Probable E3 ubiquitin-protein ligase makorin-2 OS=Homo sapiens OX=9606 GN=MKRN2 PE=1 SV=2 | 0.851239669 | 1.67 |
| MRPS24 | 28S ribosomal protein S24, mitochondrial OS=Homo sapiens OX=9606 GN=MRPS24 PE=1 SV=1 | 2.360330579 | 1.67 |
| MRPS9 | 28S ribosomal protein S9, mitochondrial OS=Homo sapiens OX=9606 GN=MRPS9 PE=1 SV=2 | 0.996694215 | 1.67 |
| RRS1 | Ribosome biogenesis regulatory protein homolog OS=Homo sapiens OX=9606 GN=RRS1 PE=1 SV=2 | 0.578512397 | 1.67 |
| BLMH | Bleomycin hydrolase OS=Homo sapiens OX=9606 GN=BLMH PE=1 SV=1 | 0.662809917 | 1.66 |
| CCDC59 | Thyroid transcription factor 1-associated protein 26 (Fragment) OS=Homo sapiens OX=9606 GN=CCDC59 PE=1 SV=1 | 1.851239669 | 1.66 |
| CSTA | Cystatin-A OS=Homo sapiens OX=9606 GN=CSTA PE=1 SV=1 | 1.966942149 | 1.66 |
| DHX33 | ATP-dependent RNA helicase DHX33 OS=Homo sapiens OX=9606 GN=DHX33 PE=1 SV=2 | 2.289256198 | 1.66 |
| GNB3 | Guanine nucleotide-binding protein G(I)/G(S)/G(T) subunit beta-3 (Fragment) OS=Homo sapiens OX=9606 GN=GNB3 PE=1 SV=8 | 14.54710744 | 1.66 |
| HPRT1 | Hypoxanthine phosphoribosyltransferase OS=Homo sapiens OX=9606 PE=2 SV=1 | 2.01322314 | 1.66 |
| RRBP1 | RRBP1 protein OS=Homo sapiens OX=9606 GN=RRBP1 PE=2 SV=1 | 0.016528926 | 1.66 |
| SEC61A1 | Protein transport protein Sec61 subunit alpha isoform 1 OS=Homo sapiens OX=9606 GN=SEC61A1 PE=1 SV=1 | 1.639669421 | 1.66 |
| RRP12 | RRP12-like protein OS=Homo sapiens OX=9606 GN=RRP12 PE=1 SV=2 | 1.327272727 | 1.65 |
| PRPF6 | Pre-mRNA-processing factor 6 OS=Homo sapiens OX=9606 GN=PRPF6 PE=1 SV=1 | 2.062809917 | 1.64 |
| PTTG | Pituitary tumor transforming gene protein OS=Homo sapiens OX=9606 GN=PTTG PE=2 SV=1 | 1.525619835 | 1.64 |
| CKB | Creatine kinase brain isoform 2 (Fragment) OS=Homo sapiens OX=9606 GN=CKB PE=2 SV=1 | 0.816528926 | 1.63 |
| KPNB1 | Importin subunit beta-1 OS=Homo sapiens OX=9606 GN=KPNB1 PE=1 SV=2 | 1.423140496 | 1.63 |
| PLK1 | Polo-like kinase 1 (Drosophila), isoform CRA_b OS=Homo sapiens OX=9606 GN=PLK1 PE=4 SV=1 | 3.775206612 | 1.63 |
| CUL4B | Cullin-4B OS=Homo sapiens OX=9606 GN=CUL4B PE=1 SV=4 | 1.634710744 | 1.62 |
| NDUFA10 | NADH dehydrogenase [ubiquinone] 1 alpha subcomplex subunit 10, mitochondrial OS=Homo sapiens OX=9606 GN=NDUFA10 PE=1 SV=1 | 1.890909091 | 1.62 |
| RO60 | 60 kDa SS-A/Ro ribonucleoprotein OS=Homo sapiens OX=9606 GN=RO60 PE=1 SV=2 | 1.507438017 | 1.62 |
| SDHA | Succinate dehydrogenase [ubiquinone] flavoprotein subunit, mitochondrial OS=Homo sapiens OX=9606 GN=SDHA PE=1 SV=2 | 3.080991736 | 1.62 |
| ELOB | Elongin-B OS=Homo sapiens OX=9606 GN=ELOB PE=1 SV=1 | 1.274380165 | 1.61 |
| EXOSC6 | Exosome complex component MTR3 OS=Homo sapiens OX=9606 GN=EXOSC6 PE=1 SV=1 | 0.682644628 | 1.61 |
| LOC340312 | Similar to Importin alpha-2 subunit (Karyopherin alpha-2 subunit) (SRP1-alpha) (RAG cohort protein 1) OS=Homo sapiens OX=9606 GN=LOC340312 PE=4 SV=1 | 1.547107438 | 1.61 |
| PTCD3 | Pentatricopeptide repeat domain-containing protein 3, mitochondrial (Fragment) OS=Homo sapiens OX=9606 GN=PTCD3 PE=1 SV=3 | 1.107438017 | 1.61 |
| SPTLC1 | Serine palmitoyltransferase, long chain base subunit 1, isoform CRA_a OS=Homo sapiens OX=9606 GN=SPTLC1 PE=4 SV=1 | 1.079338843 | 1.61 |
| DDX51 | ATP-dependent RNA helicase DDX51 OS=Homo sapiens OX=9606 GN=DDX51 PE=2 SV=1 | 1.056198347 | 1.6 |
| DHX37 | Probable ATP-dependent RNA helicase DHX37 OS=Homo sapiens OX=9606 GN=DHX37 PE=1 SV=1 | 0.016528926 | 1.6 |
| DNMT3A | DNA (cytosine-5)-methyltransferase 3A OS=Homo sapiens OX=9606 GN=DNMT3A PE=1 SV=4 | 2.499173554 | 1.6 |
| SAMHD1 | Deoxynucleoside triphosphate triphosphohydrolase SAMHD1 OS=Homo sapiens OX=9606 GN=SAMHD1 PE=1 SV=2 | 1.054545455 | 1.6 |

**Supplemental Table S4. MEME analysis of [KRPILG][V]x[FW] motif occurrence in Ebola NP protein (p<0.01).**

| **Sequence Name** | **Start** | **End** | **p-value** | **q-value** | **Matched Sequence** |
| --- | --- | --- | --- | --- | --- |
| NP | 675 | 678 | 0.000694 | 0.305 | PVVF |
| NP | 63 | 66 | 0.00083 | 0.305 | GVDF |
| NP | 483 | 486 | 0.00131 | 0.32 | LVLF |
| NP | 198 | 201 | 0.003 | 0.551 | MVIF |
| NP | 723 | 726 | 0.0057 | 0.804 | PVMN |
| NP | 188 | 191 | 0.0074 | 0.804 | PTAW |
| NP | 42 | 45 | 0.00875 | 0.804 | PVYQ |
| NP | 609 | 612 | 0.00875 | 0.804 | PVYR |

**Supplemental Table S5. MEME analysis of [F]xx[KR]x[KR] motif occurrence in Ebola NP protein (p<0.01).**

| **Sequence Name** | **Start** | **End** | **p-value** | **q-value** | **Matched Sequence** |
| --- | --- | --- | --- | --- | --- |
| NP | 106 | 111 | 0.000331 | 0.243 | FEVKKR |
| NP | 379 | 384 | 0.00119 | 0.438 | FHQKKN |
| NP | 648 | 653 | 0.00309 | 0.672 | FEEMYR |
| NP | 104 | 109 | 0.00405 | 0.672 | FRFEVK |
| NP | 725 | 730 | 0.00459 | 0.672 | MNHKNK |
| NP | 208 | 213 | 0.00701 | 0.856 | FLIKFL |
